# Meta- and cross-species analyses of insulin resistance based on gene expression datasets in human white adipose tissues

**DOI:** 10.1038/s41598-017-18082-7

**Published:** 2018-02-27

**Authors:** Junghyun Jung, Go Woon Kim, Woosuk Lee, Changsoo Mok, Sung Hyun Chung, Wonhee Jang

**Affiliations:** 1 Department of Life Science, Dongguk University, 30 Pildong ro 1-gil, 04620 Seoul, Korea; 2 Department of Pharmacology, College of Pharmacy, Kyung Hee University, 26 Kyungheedae-ro, 02447 Seoul, Korea

## Abstract

Ample evidence indicates that insulin resistance (IR) is closely related to white adipose tissue (WAT), but the underlying mechanisms of IR pathogenesis are still unclear. Using 352 microarray datasets from seven independent studies, we identified a meta-signature which comprised of 1,413 genes. Our meta-signature was also enriched in overall WAT in *in vitro* and *in vivo* IR models. Only 12 core enrichment genes were consistently enriched across all IR models. Among the meta-signature, we identified a drug signature made up of 211 genes with expression levels that were co-regulated by thiazolidinediones and metformin using cross-species analysis. To confirm the clinical relevance of our drug signature, we found that the expression levels of 195 genes in the drug signature were significantly correlated with both homeostasis model assessment 2-IR score and body mass index. Finally, 18 genes from the drug signature were identified by protein-protein interaction network cluster. Four core enrichment genes were included in 18 genes and the expression levels of selected 8 genes were validated by quantitative PCR. These findings suggest that our signatures provide a robust set of genetic markers which can be used to provide a starting point for developing potential therapeutic targets in improving IR in WAT.

## Introduction

The concomitant global epidemics of obesity and type 2 diabetes mellitus (T2DM) suggest that there is a strong correlation between the two disease conditions. Insulin resistance (IR) has been suggested as a probable linkage between obesity and T2DM and there has been an upsurge of studies in the pathophysiology of IR in an effort to develop rational treatment options for T2DM^1,2^. IR is observed in nearly all obese subjects^3^. Paradoxically, an adipose tissue deficiency condition known as lipodystrophy is also accompanied by IR^4^. Adipose tissue dysfunction could be a unifying hypothesis that explains these contradictory medical conditions that have decreased insulin sensitivity in common. It is therefore valuable to carry out an in-depth investigation on the molecular biology of adipose tissue to fully understand the progression of IR to T2DM.

Since the 2000 s, researchers have utilized gene expression profiling via high-throughput studies to understand disease pathogenesis and/or progression^5^. Although these datasets are available in public repositories and are effectively used to identify novel biomarkers and potential druggable targets, the risk of making a mismatch across different studies cannot be completely avoided owing to small sample sizes, non-biological batch effects, and heterogeneity in individual studies. To address these shortcomings and elucidate the underlying mechanism of IR development, it is necessary to integrate several studies using meta-analysis, which combines the results of multiple studies with the same hypotheses to increase statistical power^6^. High-throughput studies using microarrays have been performed in animal models to identify the possible reasons for human diseases and hence to find the potential therapeutic options^7,8^. The rationale behind these studies is that if different species are evolutionarily homologous, both biological processes and transcriptional changes will be similar in the same disease status^9^. Thus, cross-species comparison studies are valuable in that they allow for application of pharmacogenomics animal model studies to humans.

Here, we present the first meta-analysis study using microarray expression datasets of subcutaneous adipose tissues in human IR subjects. Using the results from human IR meta-analysis, so-called a meta-signature, we confirmed the robustness of the study via cross-species analyses and pharmacogenomics studies. Finally, we propose potential drug target genes, a drug signature, using network analysis. Our results may provide information that reveals the pathology of IR and aids in generating novel therapeutic targets for IR in white adipose tissue (WAT).

## Results

### Identifying a meta-signature in human IR datasets

**Figure 1:**
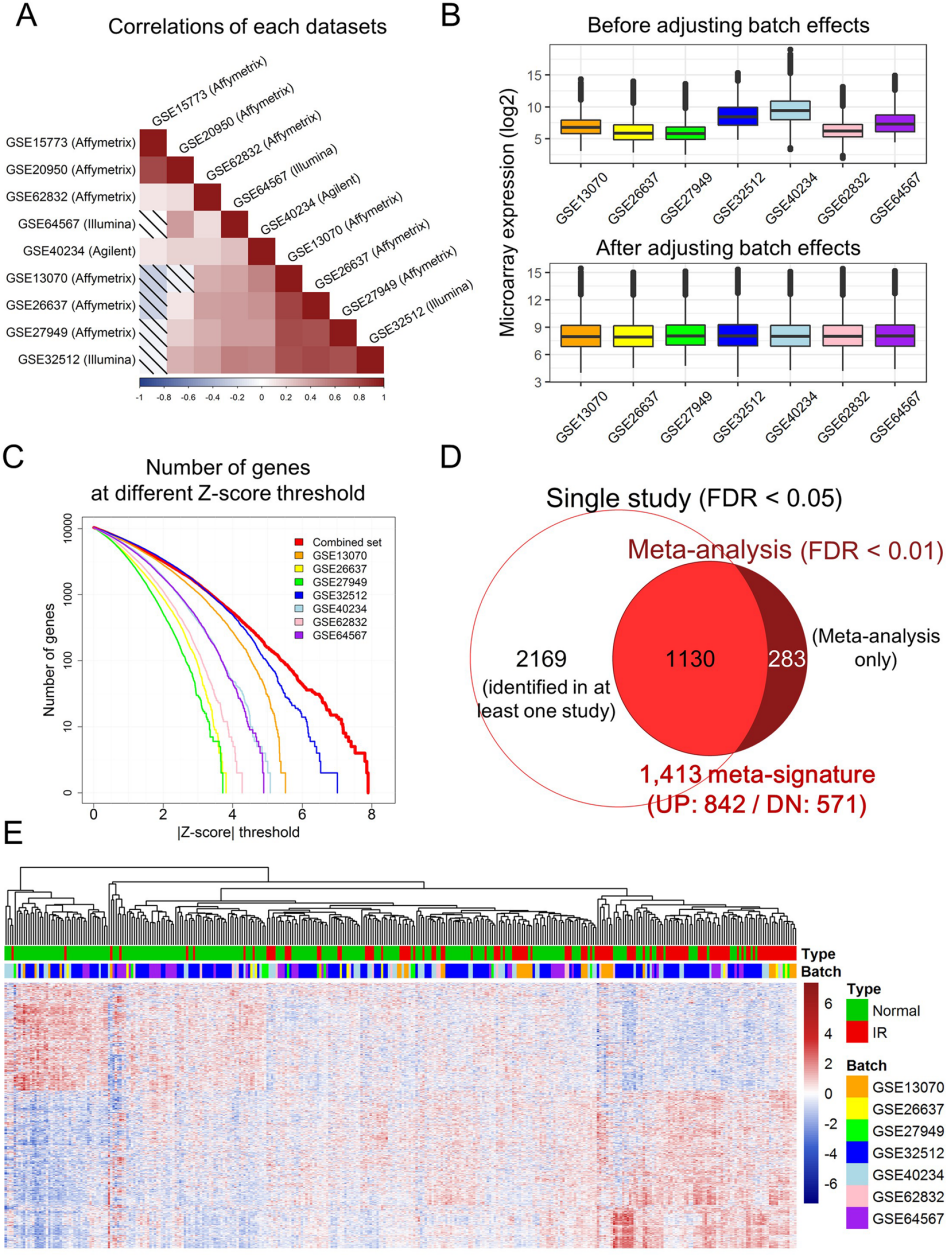
Identification of human IR meta-signatures. (**A**) Pair-wise Pearson correlations in the microarray datasets using meta-analysis Z-scores derived from the GeneMeta R package. The shades indicate negative correlation and the scale bar shows the Pearson correlation coefficient (r). (**B**) The distribution of the 7 selected datasets before and after adjusting for batch effects. (**C**) The number of genes identified using different Z-score thresholds using the 7 datasets and combined meta-analysis datasets. (**D**) A venn diagram showing the overlap between genes identified by at least one study (FDR < 0.05) and the meta-analysis (FDR < 0.01). (**E**) A heatmap showing 1,413 meta-signatures after adjusting for 7 batch effects between 233 IR and 119 normal samples. The two columns on the right indicate a summary of all IR and normal samples. DN, down; NES, normalized enrichment score; FDR, false discovery rate.

In order to identify robust genetic markers related to development and/or progression of IR using meta-analyses in humans, a total of 352 microarray datasets in seven independent studies were used, consisting of 233 IR samples and 119 normal samples (Table 1 and Supplementary Figure S1). Even though the datasets used in our meta-analysis came from different microarray platforms, including 9 datasets from Illumina, Affymetrix, and Agilent (Table 1), the datasets showed positive correlations across microarray platforms except for two Affymetrix datasets (Fig. 1A). Those two datasets (GSE15773 and GSE20950) were excluded from this study. The two data were obtained from morbidly obese patients with BMIs of ≥50 who required gastric bypass surgery^10^. Some of these patients were insulin sensitive and some were IR. Their gene expression patterns were completely different from those seen in patients with BMIs <40, so the datasets were eliminated. In the case of GSE62832, which was included in our meta-analysis, extremely obese subjects (BMI ≥ 40) were also excluded to minimize other health risks induced by weight gain^11^. The selected dataset had a heterogeneous distribution due to batch effects which arose from non-biological variations. When batch effects were adjusted for using the ComBat function in the SVA R package, the distribution became homogeneous (Fig. 1B). Next, a meta-analysis was performed to identify a meta-signature using the GeneMeta R package. The power of the meta-analysis showed that the number of significant genes was increased compared with that seen in each individual study with different Z-score cutoffs (Fig. 1C). Some specific genes were identified only in the meta-analysis despite the use of more stringent statistical criteria (FDR < 0.01) than were used in each single study (FDR < 0.05) (Fig. 1D). We found 1,413 genes that were either identified previously or newly in the meta-analysis and the genes made up a meta-signature (FDR < 0.01). Among the meta-signature, 842 gene were up-regulated (Z-score > 0) and 571 gene were down-regulated (Z-score < 0) (Fig. 1E and Supplementary Table S1).

**Table 1:** Characteristics of the NCBI GEO datasets used for the meta-analysis.

	GSE	Human tissue type	Array number (IR:Normal)	Platform	PMID
1	GSE13070	abdominal subcutaneous adipose	28:6	Affymetrix Human Genome U133 Plus 2.0 Array	PMID:19841271^24^
2	GSE26637	abdominal subcutaneous adipose	5:5	Affymetrix Human Genome U133 Plus 2.0 Array	PMID:22471940^66^
3	GSE27949	abdominal subcutaneous adipose	8:13	Affymetrix Human Genome U133 Plus 2.0 Array	PMID:21426570^67^
4	GSE32512	abdominal subcutaneous adipose	30:129	Illumina HumanHT-12 V3.0 expression beadchip	PMID:23557707^68^
5	GSE40234	abdominal subcutaneous adipose	34:28	Agilent-014850 Whole Human Genome Microarray 4 × 44 K G4112F	PMID:22958899^69^
6	GSE62832	abdominal subcutaneous adipose	7:11	Affymetrix Human Gene 1.0 ST Array	PMID:25555214^11^
7	GSE64567	abdominal subcutaneous adipose	7:41	Illumina HumanHT-12 V4.0 expression beadchip	PMID:25830378^70^

### Functional annotation of IR using gene set enrichment analysis

**Figure 2:**
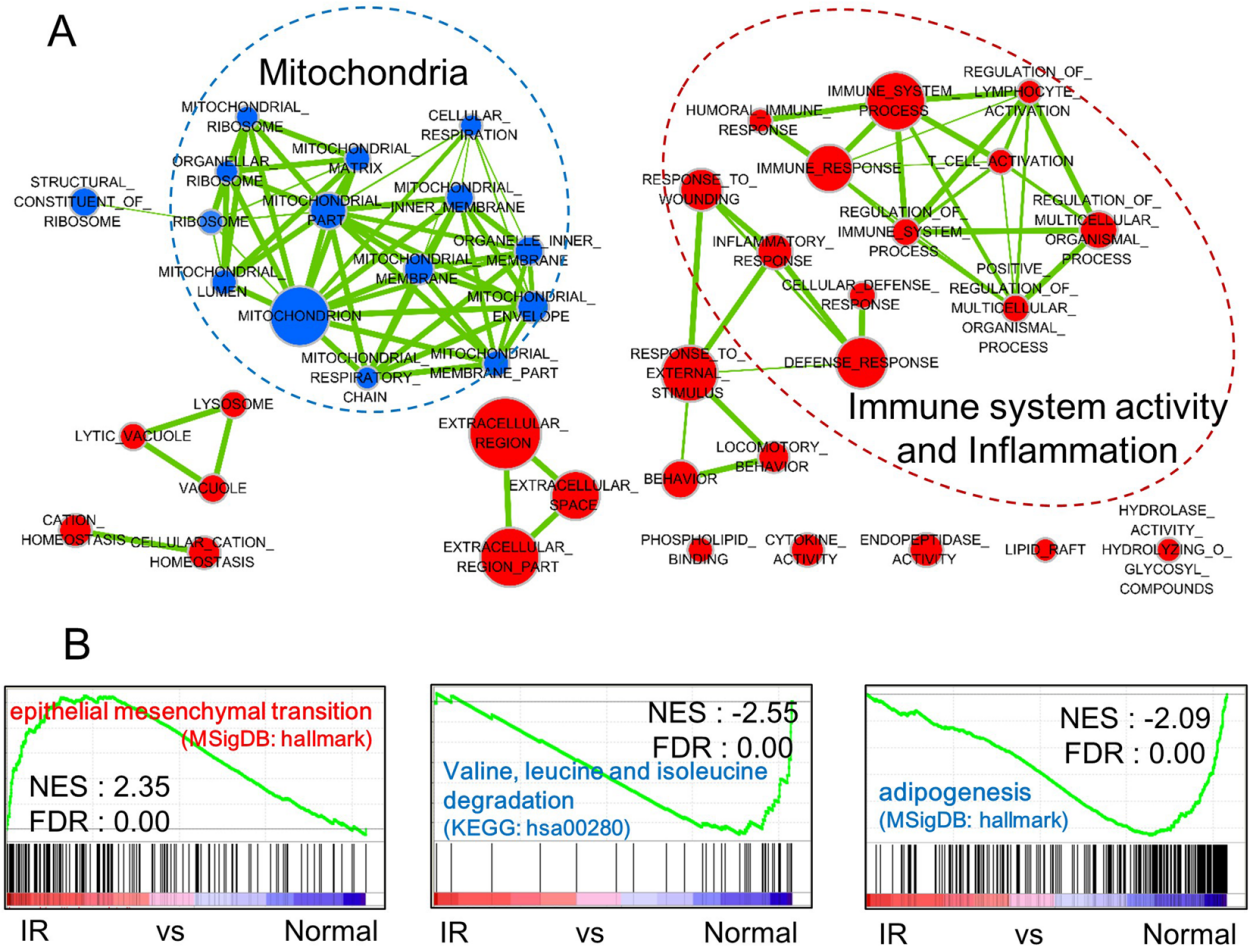
Functional annotation of IR using GSEA. (**A**) GSEA analysis using Z-scores of the meta-analysis and gene ontology (GO) gene sets. The results were visualized with an enrichment map. The node size corresponds to the number of genes in each GO term and the edge thickness corresponds to the overlap between the GO terms. The red and blue nodes correspond to significant up- and down-regulated GO terms, respectively (FDR < 0.01). (**B**) GSEA analysis using KEGG and hallmark gene sets in the Molecular Signatures Database (MSigDB). DN, down; NES, normalized enrichment score; FDR, false discovery rate.

To determine the biological functions related to the results of the meta-analysis in human IR datasets, we performed Gene Ontology (GO) analysis using gene set enrichment analysis (GSEA) based on Z-scores from the meta-analysis. GO analysis provides functional annotation and categorization into 3 domains: cellular components, biological processes, and molecular function. The results showed that most immune system activity, inflammation, and some lysosome- and extracellular region-related terms were positively enriched, while almost all mitochondrial terms were negatively enriched (Fig. 2A). In addition, we found that epithelial mesenchymal transition (EMT) (MSigDB: hallmark) was positively enriched, while valine, leucine and isoleucine degradation (KEGG: hsa00280) was negatively enriched (Fig. 2B and Supplementary Figure S2) using the KEGG and Hallmark gene sets in MSigDB.

### Confirmation of the robustness of the meta-signature using cross-species analysis

**Figure 3:**
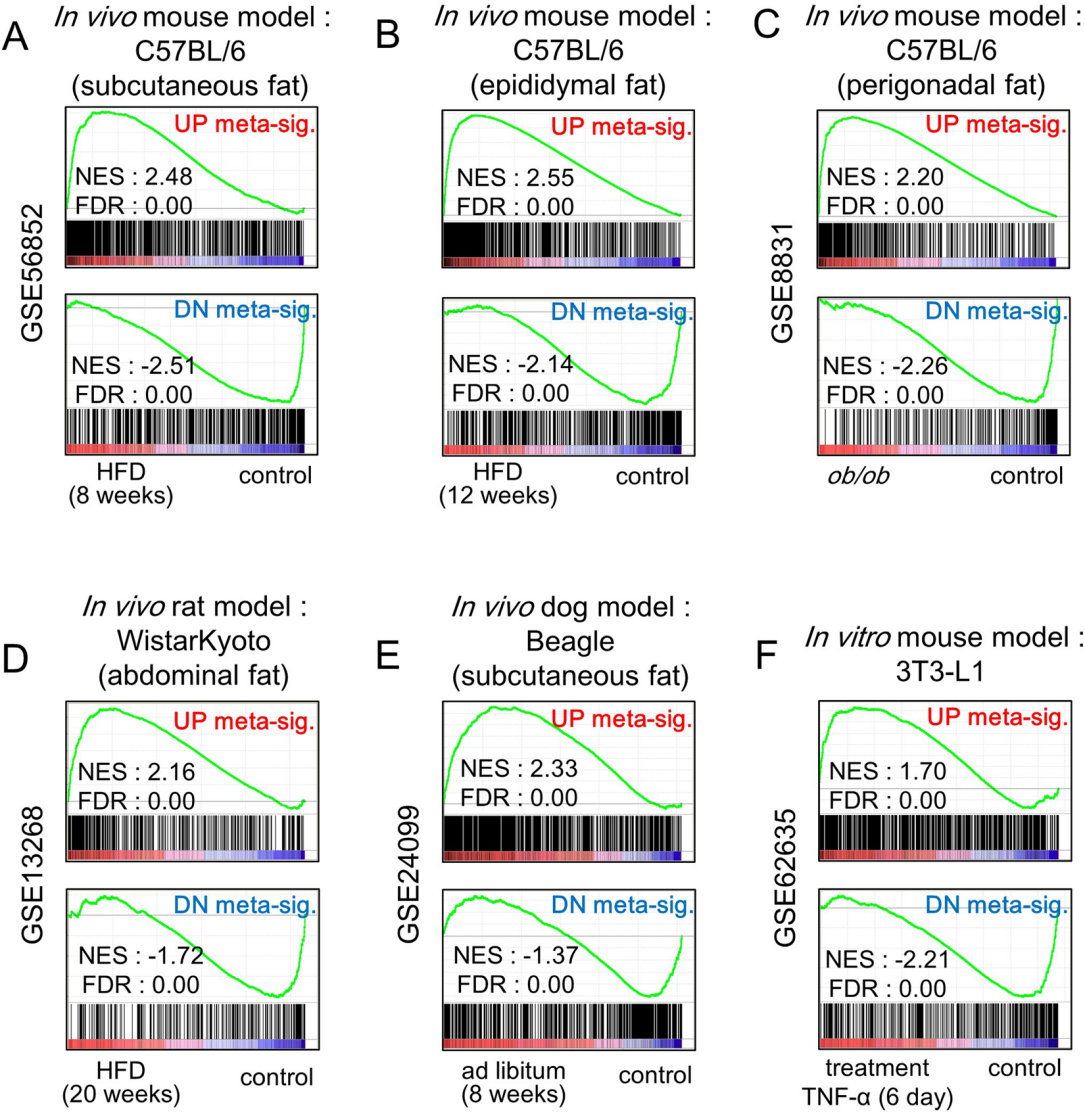
Human IR meta-signatures as robust genetic markers of IR in overall WAT from other organisms. GSEA plots showing that the human IR meta-signatures were significantly enriched in (**A**) subcutaneous, (**B**) epididymal, and (**C**) perigonadal adipose tissue derived from the IR model or *ob/ob* C57BL/6 mouse in C57BL/6 strain. GSEA plots showing that the human IR meta-signatures were also significantly enriched in (**D**) abdominal fat derived from a rat IR model in Wistar Kyoto strain, (**E**) subcutaneous fat derived from the dog (beagle) IR model, and (**F**) the *in vitro* IR model (3T3-L1) treated with tumor necrosis factor α. The GEO accession number of each dataset is on the left of the GSEA plot. DN, down; NES, normalized enrichment score; FDR, false discovery rate.

To address whether the meta-signature is a set of robust genetic markers of IR, cross-species analysis was conducted using GSEA. Because GSEA allows mapping from various platforms, obtained from other animal models to human gene symbols, our human IR meta-signature gene sets, consisting of 824 up-regulated genes and 571 down-regulated genes, were applied to microarray studies on other model organisms, such as mice, rats, dogs, and swine (Fig. 3). The results of cross-species analysis showed that the meta-signature was significantly enriched in microarray studies on subcutaneous adipose tissue in a mouse model of IR induced by feeding a high fat diet (HFD) (Fig. 3A). Notably, the result was consistent in microarray studies using other types of WAT, epididymal and perigonadal fat tissues, from a HFD-induced IR model and *ob/ob* mice, respectively (Fig. 3B and C). In addition, the meta-signature gene sets were significantly enriched in microarray data from rat abdominal fat (Fig. 3D), dog subcutaneous fat (Fig. 3E), and an *in vitro* IR model derived from 3T3-L1 cells treated with tumor necrosis factor α (TNF-α) (Fig. 3F). Our human IR meta-signature was highly enriched in the three model organisms except for swine, because the fat metabolism in pig is profoundly different from those in other model organisms. Therefore, we excluded the swine data from our analysis^12^.

Next, we identified the leading-edge subset to determine core enrichment genes in all IR models. Notably, among the 1,413 genes which made up the meta-signature, only 12 (10 up-regulated and 2 down-regulated) genes were consistently enriched in all IR models we used in Fig. 3 (Table 2). These results indicate that the human IR meta-signature obtained in our study is a set of robust genetic markers of IR in various types of WAT from different *in vitro* and *in vivo* IR models.

**Table 2:** The list of core meta-signatures consistently enriched across all models in Fig. 3.

	Gene ID	Symbol	Gene name	Z-score	FDR
1	**3929**	**LBP**	**lipopolysaccharide binding protein**	**6.11**	**0.00.E + 00**
2	4883	NPR3	natriuretic peptide receptor 3	5.85	0.00.E + 00
3	**6275**	**S100A4**	**S100 calcium binding protein A4**	**5.30**	**0.00.E + 00**
4	4015	LOX	lysyl oxidase	4.65	1.47.E − 04
5	3936	LCP1	lymphocyte cytosolic protein 1	4.53	1.81.E − 04
6	**1436**	**CSF1R**	**colony stimulating factor 1 receptor**	**4.36**	**2.43.E** − **04**
7	822	CAPG	capping actin protein, gelsolin like	4.18	4.16.E − 04
8	960	CD44	CD44 molecule (Indian blood group)	4.06	5.31.E − 04
9	6696	SPP1	secreted phosphoprotein 1	3.96	7.53.E − 04
10	7805	LAPTM5	lysosomal protein transmembrane 5	3.50	2.87.E − 03
11	5264	PHYH	phytanoyl-CoA 2-hydroxylase	−3.23	5.96.E − 03
12	**56922**	**MCCC1**	**methylcrotonoyl-CoA carboxylase 1**	**−4.63**	**1.50.E** − **04**

The genes in the network in Fig. 7A were shown in bold.

### Applying the meta-signature to pharmacogenomics and gain- or loss-of-function studies

**Figure 4:**
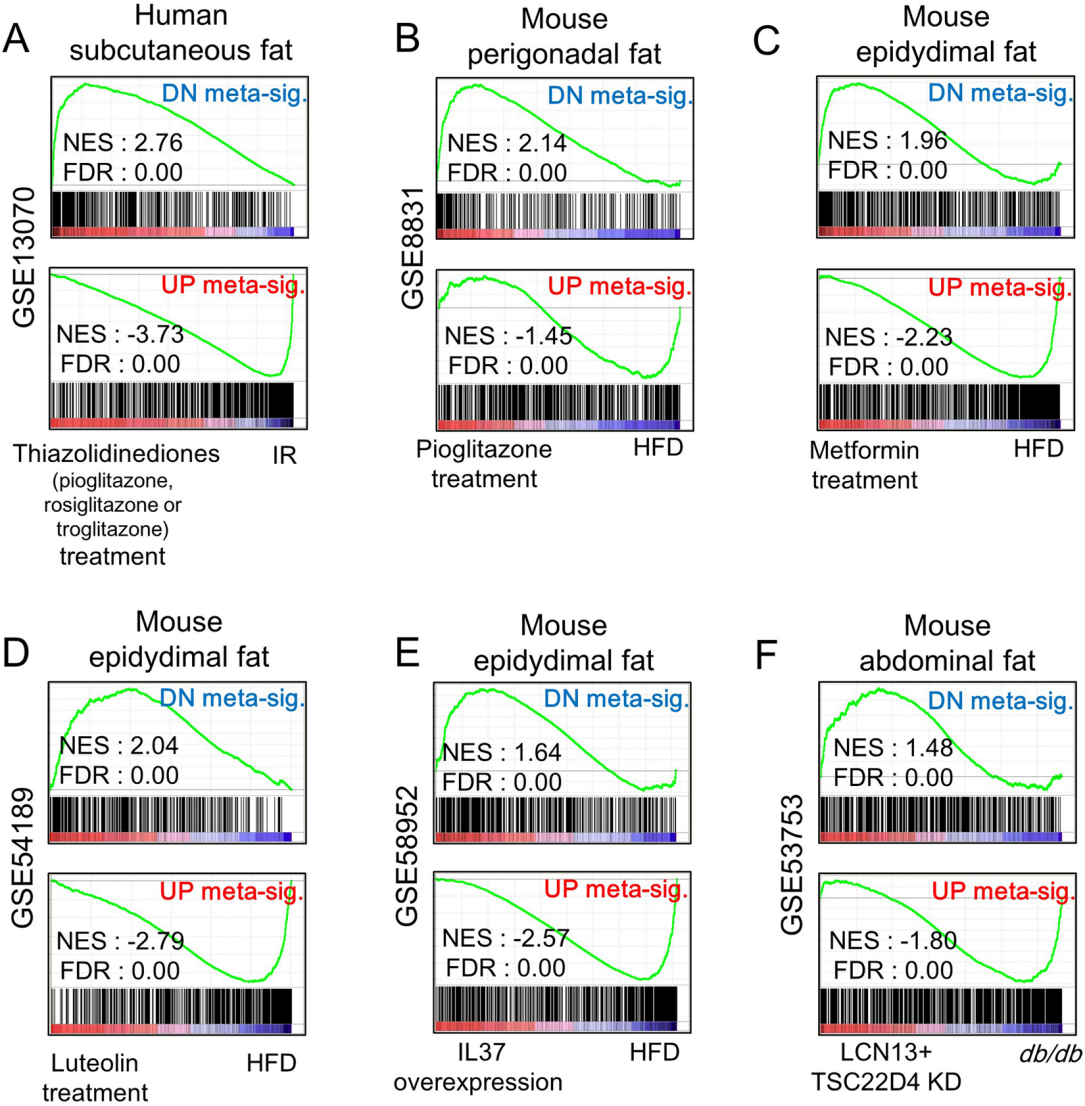
Human IR meta-signatures as potential therapeutic targets for IR. GSEA plots showing that the IR meta-signatures were inversely enriched in pharmacogenomics data treated with (**A**) thiazolidinedione (pioglitazone, rosiglitazone, or troglitazone), (**B**) pioglitazone, (**C**) metformin, and (**D**) luteolin in humans or mice. (**E**) GSEA plot showing that the IR meta-signatures were inversely enriched in interleukin (IL) 37 overexpression data in mouse epididymal fat. (**F**) GSEA plot showing that the meta-signatures were also inversely enriched in double knock down (KD) microarray datasets of transforming growth factor beta-like stimulated clone (TSC) 22 D4 and lipocalin (LCN) 13 in *db/db* mouse abdominal fat. The GEO accession number of each dataset is on the left of the GSEA plot. DN, down; NES, normalized enrichment score; FDR, false discovery rate; KD, knock down.

Several recently published studies directly compared molecular signatures of disease with the molecular effects of drug target genes using an inverse correlation approach for successful drug repositioning and disease therapies^13–21^. Thus, we hypothesized that our IR meta-signature may also be inversely enriched in pharmacogenomics datasets for improvement of IR. We carried out inverse enrichment analysis using pharmacogenomics datasets for thiazolidinediones (TZDs) and metformin, which are approved for the treatment of T2DM by the Food and Drug Administration. Metformin is a biguanide that is recommended by the American Diabetes Association as a first-line agent to treat T2DM patients^22,23^ and TZD is another class of anti-diabetic medication which includes pioglitazone and rosiglitazone, and troglitazone^24^. Consistent with our hypothesis, our meta-signature gene sets were inversely enriched in microarray datasets from IR patients treated with TZD drugs (Fig. 4A), and a mouse IR model treated with pioglitazone (Fig. 4B) and with metformin (Fig. 4C). Additionally, the meta-signature gene sets were inversely enriched in microarray datasets from luteolin-treated mice (Fig. 4D), interleukin-37-overexpressing transgenic mice (Fig. 4E), and double knock-down of transforming growth factor beta-like stimulated clone (TSC) 22 D4 and lipocalin (LCN) 13 in *db/db* mice (Fig. 4F). These results also correspond with those of recent studies showing that the flavonoid luteolin, the anti-inflammatory agent interleukin-37, and double inhibitions of TSC22D4 and LCN13 ameliorate IR^25–27^. Overall, our data suggest that the meta-signature is a set of robust genetic markers which can be used as a screening tool to determine the effects of putative candidate and/or new drugs on improving IR condition in WAT.

### Identification of potential drug targets

**Figure 5:**
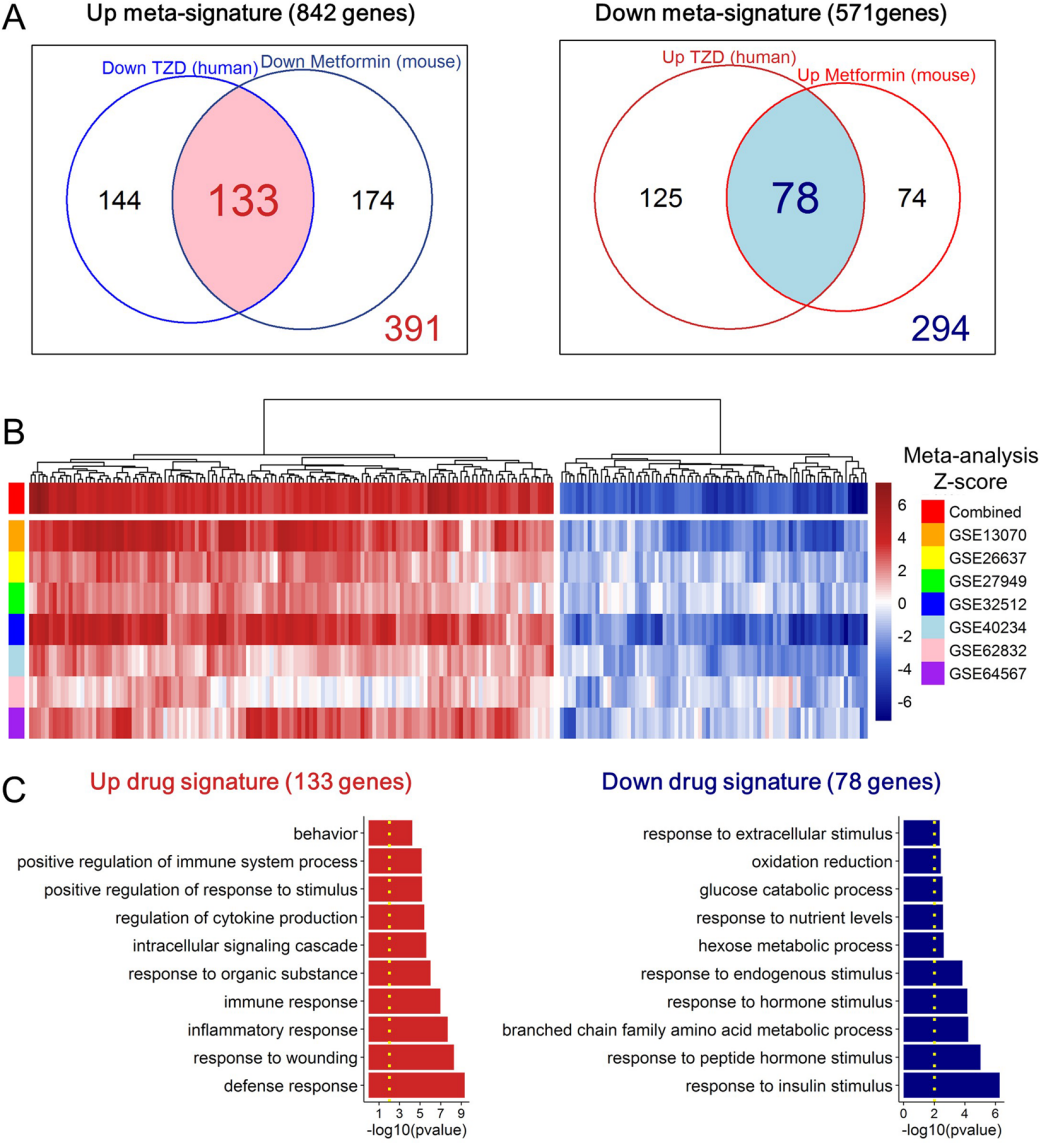
Identification of cross-species drug signature. (**A**) A Venn diagram showing the overlapping genes that were co-regulated in the microarray datasets of TZD-treated humans (Fig. 4A) and metformin-treated mice (Fig. 4B). (**B**) Gene ontology enrichment analysis showing the biological processes using the up- and the down-regulated drug signature. The yellow dotted lines indicate the threshold level for significance (p < 0.01). (**C**) A heatmap showing the meta-analysis Z-scores of 211 drug-signature genes.

The expression levels of a subset of meta-signature genes changed with the treatment of TZDs and metformin (Fig. 4A–C) which have different mechanisms of action against IR and T2DM^24,28^. Therefore, we identified overlapping genes that were co-regulated in both TZD-treated humans (Fig. 4A) and metformin-treated mice (Fig. 4B) to identify potential drug target genes that could ameliorate IR. We carried out co-regulated gene analysis using the core enrichment genes of metformin and TZDs from the GSEA results, which were the leading-edge subset within our meta-signature. The leading-edge subset derived from the GSEA results can be interpreted as the set of core enrichment genes^29^. Among the meta-signature, a total of 211 genes identified across the species were co-regulated by metformin and TZDs, and these genes made up the drug signature which is a set of genes related to drug-induced gene expression changes^30^ (Fig. 5A,B, and Supplementary Table S2). Using the 133 up-regulated drug-signature genes, we found that the GO terms for biological processes were significantly enriched in the defense response (p = 4.84e-10), inflammatory response (p = 2.16e-08), and cellular signaling cascade (p = 2.58e-06), while the enriched GO terms for the 78 down-regulated drug-signature genes were response to insulin stimulus (p = 5.32e-07), response to hormone stimulus (p = 6.82e-05), glucose catabolic process (p = 2.83e-03), and oxidation reduction (p = 3.68e-03) (Fig. 5C). Collectively, our data suggest that the cross-species drug signature, which was made up of genes that were co-regulated in response to metformin and TZDs comprise a subset of genes that are potential drug targets for improving IR status among the meta-signature.

### Clinical relevance of expression levels of the drug signature in IR and obesity

**Figure 6:**
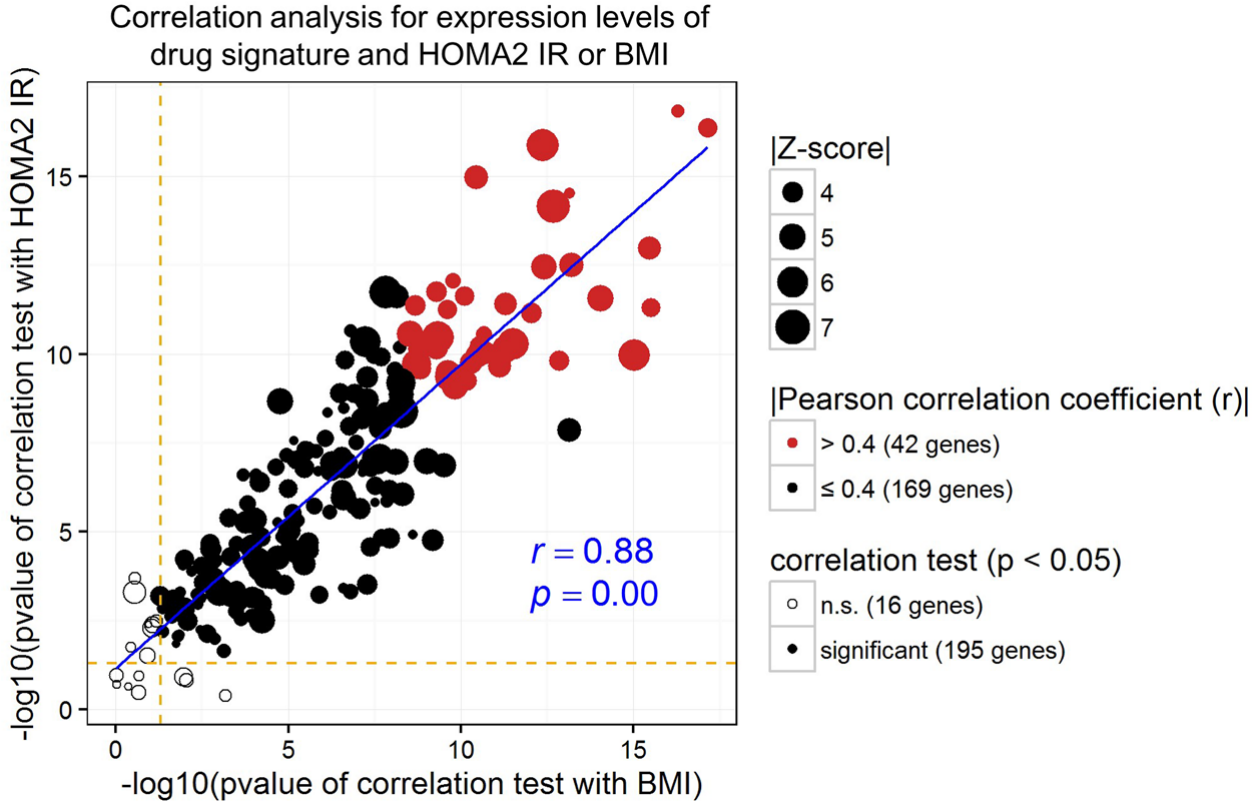
Correlation between expression levels of drug signature and both BMI and HOMA2-IR. The scatter plot showing the correlation between expression levels of 211 drug-signature genes and HOMA2-IR or BMI using GSE32512 datasets. The y-axis and x-axis show the p-value of the correlation test for HOMA2-IR and BMI, respectively. The orange dotted lines indicate the threshold level for significance (p < 0.05). The red circles represent Pearson correlation coefficient >0.4 for both the expression levels of drug-signature genes and BMI or HOMA2-IR. The blue line indicates the correlation analysis of the scatter plot. See also Supplementary Figure S3.

Our results suggested that the genes in the cross-species drug signature may contain direct/indirect results of improved IR condition, target genes of TZD- and metformin-treatments, or potential target genes for novel therapeutics. In any case, we hypothesized that the expression levels of the genes in drug signature may significantly correlate with clinical traits involved in IR. Because BMI, which is used as an indicator of obesity, is also an important factor in the pathogenesis of IR and T2DM^31^ (Supplementary Figure S3A), homeostasis model assessment2-IR (HOMA2-IR) score, which quantifies IR, and BMI were used to identify the relationship between the expression levels of 211 genes in cross-species drug signature and clinical traits. To verify this hypothesis, the expression levels of all 211 genes in GSE32512, which contained gene expression data from subcutaneous fat biopsies of 200 human subjects with clinical traits, were used. Among the 211 genes which made up our drug signature, the expression levels of 195 genes (92.4%) were significantly correlated with both HOMA2-IR and BMI (p < 0.05) (Fig. 6). Genes that were highly correlated with BMI and HOMA2-IR had significantly higher Z-scores in the meta-analysis (Supplementary Figure S3B). The results suggest that the expression levels and Z-scores of the drug signature are strongly associated with obesity and IR.

### Network construction and validation of drug signature

**Figure 7:**
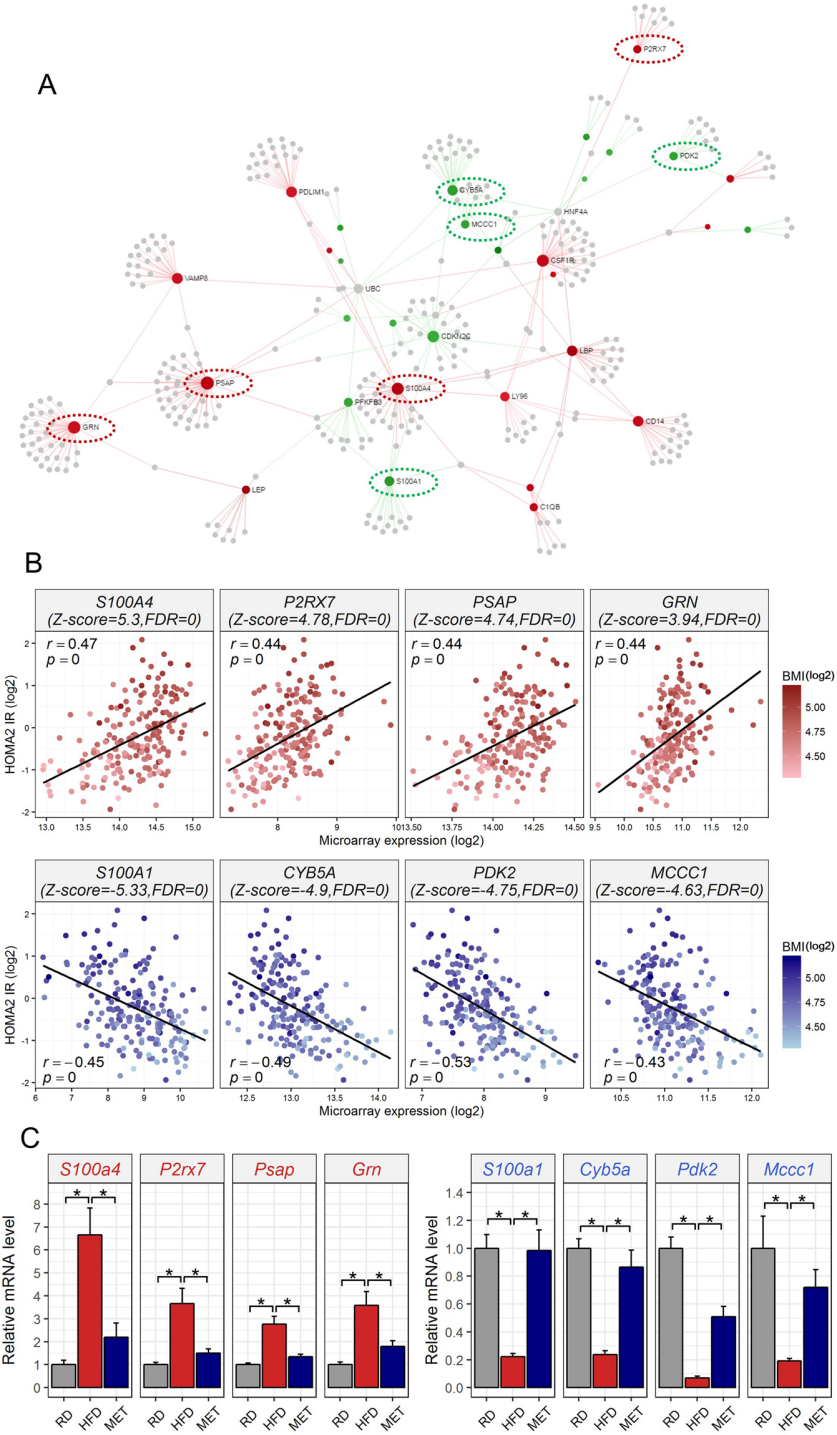
Network analysis and qPCR validation. (**A**) The network shows 362 nodes and 399 edges containing 32 drug-signature genes. Eighteen drug-signature genes (degree ≥9) are indicated by gene symbols and the red and green nodes represent the up- and the down-regulated drug-signature genes, respectively. The dotted ellipses indicate the genes for which the expression levels were validated using qPCR. (**B**) Correlation analysis between gene expression levels and HOMA2-IR or BMI. (**C**) qPCR validation. Data are presented as means ± SEM (n ≥ 3). *p < 0.05. See also Supplementary Figure S4.

Since the expression levels of the genes in the drug signature were significantly related to IR, protein–protein interaction (PPI) networks were constructed to understand the interactions of the biological processes related to the drug signature. From the drug signatures, 42 genes (22 up- and 20 down-regulated genes) which were highly correlated (|*r|* > 0.4) with both BMI and HOMA2-IR (Fig. 6) were used to establish PPI networks. Eighteen genes (network degree ≥9) among drug signature were shown in the PPI networks, contacting the up-regulated drug-signature genes, such as PSAP (prosaposin, degree = 40), GRN (granulin precursor, degree = 34), and S100A4 (S100 calcium binding protein A4, degree = 31) and the down-regulated drug-signature genes, such as CYB5A (cytochrome b5 type A (microsomal), degree = 19), S100A1 (S100 calcium binding protein A1, degree = 16), and MCCC1 (methylcrotonoyl-CoA carboxylase 1, degree = 10) (Fig. 7A). The expression levels of the genes showed either a positive or negative correlation with HOMA2-IR (Figs 7B and Supplementary Figure S4). Notably, among the 12 core meta-signatures (Table 2), proteins encoded by 4 genes (*LBP, S100A4, CSF1R, and MCCC1*) were in the network (Fisher’s exact test, p < 0.01). We randomly selected 8 genes, *S100a4*, purinergic receptor P2X 7 *(P2rx7), Psap, Grn*, S100 calcium binding protein A1 (*S100a1*)*, Cyb5a*, pyruvate dehydrogenase kinase 2 (*Pdk2)*, and *Mccc1* and quantitative PCR (qPCR) was performed to validate the expression levels obtained from PPI networks using mouse epididymal fat treated with metformin (Fig. 7C). The results indicated that the expression levels of *S100a4*, *P2rx7*, *Psap*, and *Grn* in epididymal fat were significantly up-regulated in HFD-fed mice, while those of *S100a1*, *Cyb5a*, *Pdk2*, and *Mccc1* were significantly down-regulated.

## Discussion

In this study, we show that meta-analysis provides valuable information for generalizing the results of multiple studies and investigating the underlying mechanisms of IR in human WAT. One of the major advantages of meta-analysis is that it allows us to overcome the shortcomings of the limited number of available samples in a single study by increasing statistical power and determine whether there is a consensus in transcriptome changes (Fig. 1). For the meta-analysis techniques used in this study, the method of Choi *et al*.^32^ described by the GeneMeta R package was first chosen for a two-class comparison, disease vs. normal state^33^. Because the meta-signature based on gene-wise combined Z-scores can directly be applied to GSEA using the GSEAPre-ranked method. These methods are very useful tools for conducting meta-analysis and functional enrichment analysis. Furthermore, GSEA provides built-in tools for conversion between several organisms’ microarray identifiers (i.e., platform probe identifiers) to human gene symbols, which can then be easily applied in cross-species analysis.

WAT was initially regarded as a passive energy repository, but now is considered to be an endocrine organ that secretes adipokines such as leptin, adiponectin, TNF-α, and interleukin-6 (IL-6), which are common mediators of adipose tissue, inflammation, and immunity^34^. In humans and mice, obesity changes adipose tissue metabolism and increases accumulation of macrophages and other immune cells in WAT^35^. Macrophages are then stimulated by monocyte chemoattractant protein-1 to infiltrate WAT^36^. In the state of obesity, excessive energy triggers NF-κB signaling pathways and may disrupt insulin signaling pathway via induction of TNF-α secretion by WAT, which in turn causes IR^37^. As expected, our results indicated that the up-regulated human IR meta-signature was significantly enriched in immune system activity and inflammation GO terms (Fig. 2A) as well as TNF-α signaling via NF-κB (data not shown). NF-κB can promote cell progression and oncogenesis and its signaling pathway is fundamental to the induction and maintenance of EMT^38^. We found that EMT-related genes are significantly associated with the up-regulated meta-signature (Fig. 2B), suggesting that macrophage infiltration in WAT may be associated with EMT because TNF-α signaling via NF-κB is closely related to inflammation and macrophage infiltration.

Although we performed our meta-analysis using gene expression datasets in subcutaneous WAT from human IR patients, cross-species analysis confirmed that our human IR meta-signature can be applied to animal IR or T2DM model datasets derived from overall WAT, including visceral adipose tissue as well as an *in vitro* IR model (Fig. 3). Our meta-signature can also be utilized to perform inverse correlation approaches using drug candidates and gain- or loss-of-function microarray datasets for attenuating T2DM or IR (Fig. 4). Even though subcutaneous and visceral fat is known to have different roles in IR^34^, our data showed that the gene expression patterns in the two types of fat are similar. Because the enrichment score of mouse IR model data was the highest among model organisms (Fig. 3), the mouse IR model can be optimally applied in human IR studies. Overall, our results sugget that the human IR meta-signature might be utilized as a pre-screening tool for developing diabetes treatments or insulin-sensitizing drugs.

The American Diabetes Association recommends metformin as a first-line agent to treat T2DM patients^22,23^; however, the mechanism of action of metformin is not completely understood^39^. Recent studies suggested that the anti-diabetic effect of metformin may involve 5′ AMP-activated protein kinase (AMPK)-dependent mechanisms, but there remains some controversy as to whether the activation of AMPK by metformin is direct^40,41^. TZDs were approved for treatment of T2DM because they decrease IR and are known to be PPARγ activators^24^. Even though some members of TZDs may have toxicity^42,43^, there already is ample evidence that metformin- and/or TZD-treatment alter whole body or adipose tissue insulin sensitivity in humans as well as in mice^24,28,44^. Together, these findings show the necessity for a deeper understanding of the underlying mechanisms of action of the drugs currently in use. Through identification of the cross-species drug signature that is co-regulated by TZDs (Fig. 4A) as well as metformin (Fig. 4B) and through considering clinical relevance via correlations between the expression of genes in the drug signature and HOMA2-IR, we were able to determine gene sets which are responsive to IR condition in overall WAT. Although we could not conduct a correlation test between gene expression responses and the efficacy of the metformin/TZD due to the insufficient clinical information on the data, we tried to validate the correlation between drug signature expression responses and HOMA2-IR using GSE32512 which is the largest dataset with clinical information (Fig. 6).

Consistent with the results of functional enrichment analysis using up-regulated meta-signature genes (Fig. 2A), we found that the up-regulated drug signature is also closely related to inflammation and immune response (Fig. 5). Among 8 selected genes from 18 genes in the network (degree ≥9) (Fig. 7), *Grn* is known to be an important regulator of obesity and IR^45^. Knock-out mice of *Grn* were prevented from becoming IR induced by HFD, whereas the expression levels of the protein and mRNA are significantly increased in WAT derived from HFD-fed mice. Moreover, treating pioglitazone to *ob/ob* mice reduced the expression level of *Grn*, suggesting that *GRN* can directly regulate IR condition and is a target of a TZD. Among 8 genes shown in Fig. 7C, *PDK2, CYB5A*, and *MCCC1* are known to have direct relationships with IR^46–49^. Additionally, the cross-species drug signature in the PPI network included calcium-binding S100 family proteins, among which *S100A4* and *S100A1* had indirect relationships with IR^50–54^ and the expression patterns were validated by qPCR (Fig. 7C).

We would like to comment that our study is not without limitations. First, we could not control all the variables that could affect adipose gene expression apart from IR, but we tried to minimize the possible contribution of obesity on the signatures by including lean and obese subjects in both insulin sensitive and IR datasets. The expression levels of the genes in our drug signature were compared with both BMI and HOMA2-IR only, because we were more focused on transcriptome levels in WAT than other clinical characteristics such as age, gender, and serum levels of metabolites. Second, we assessed the validity of our results with minimal experimentation, thus more extensive experiments may be necessary to validate our study.

Despite these limitations, however, we strongly believe that our meta-analysis successfully generalized the results of seven studies and identified robust genetic markers of IR. The results may also provide a starting point for identifying potential therapeutic targets. Using meta-analysis based on gene expression microarrays in subcutaneous WAT from human IR patients, we herein showed that the results of our meta-analysis, meta-signature, are robust genetic markers for *in vitro* and *in vivo* IR models in overall WAT. Among the meta-signature, we identified the cross-species drug signature that are co-regulated by both metformin and TZDs and are closely associated with both HOMA2-IR and BMI, suggesting that our results can be a good starting point for identifying genetic markers, potential drug targets, or therapeutic targets for IR.

## Methods

### Data collection for meta-analysis

The NCBI Gene Expression Omnibus (GEO) was queried for one-channel (one color) microarray gene expression datasets that contain human IR subjects who had not been given drug treatment before biopsy sampling. If the IR status of a given individual in the datasets was not given, HOMA2-IR based on fasting glucose and insulin levels was calculated using the HOMA2-IR calculator (https://www.dtu.ox.ac.uk/homacalculator/). The cutoff value for classification of IR subjects was HOMA2-IR ≥1.7, based on a previous study^55^. To make a clear distinction between IR and insulin sensitive (normal), subjects with HOMA2-IR <1 were regarded as insulin sensitive.

### Microarray preprocessing and meta-analysis

Affymetrix microarray datasets were preprocessed and normalized following Jung *et al*.^56^. For Illumina and Agilent datasets, quantile normalization was performed using the limma R package^57^. After preprocessing, the datasets were combined using probe’s Entrez ID and ComBat^58^ function and the surrogate variable analysis (SVA) R package^59^ was applied to adjust for batch effects. Finally, a meta-analysis was carried out using the random effects model (REM) method in the GeneMeta R package proposed by Choi *et al*.^32^ to compute Z-scores and the false discovery rate (FDR).

### Enrichment and cross-species analysis using gene set enrichment analysis

Functional enrichment analyses were performed based on Z-scores using the GSEAPreranked method in gene set enrichment analysis (GSEA)^29,60^. Gene ontology (GO) gene sets in the Molecular Signatures Database (MSigDB)^61^ were applied to GSEA. The results of GO enrichment analysis were visualized using an Enrichment Map^62^ with default settings. Hallmark and Kyoto encyclopedia of genes and genomes (KEGG)^63^ pathway gene sets in MSigDB were also used. For cross-species analysis, the up- (842 genes) and the down-regulated (571 genes) meta-signature obtained from our meta-analysis were used as gene sets for GSEA. Microarrays of log fold-change (FC) levels were considered as a pre-ranked list for cross-species analysis. For co-expression analysis using thiazolidinedione (TZD)- and metformin-treated microarray datasets, leading-edge subset derived from GSEA results were used. This subset of genes made the most contribution to the enrichment results^29^. In addition, the database for annotation, visualization and integrated discovery (DAVID)^64,65^ tool was used to perform functional enrichment analysis for selected genes.

### Epididymal tissue sampling and RNA extraction

Mouse epididymal adipose tissue samples were obtained from a previous study conducted at Kyung Hee University with the approval of the Institutional Animal Care and Use Committee (IACUC) of Kyung Hee University (IACUC approval No. KHUASP(SE)-15-069). Experimental procedures were carried out in accordance with relevant guidelines and regulations. During the study, five-week-old C57BL/6 mice (Envigo, Indianapolis, IN, USA) were housed in a temperature- (22 ± 2 °C) and humidity-controlled (50 ± 5%) room with a 12/12 h light/dark cycle and free access to food and water. Tissue samples used were from mice in three different treatment groups: a regular diet-fed (RD) group, a high fat diet-fed (HFD) group, and an HFD plus 300 mg/kg of metformin treatment (MET) group. The RD (10% kcal from fat; D12450B) and HFD (60% kcal from fat; D12492) were purchased from Research Diets (New Brunswick, NJ, USA). Mice were orally administered with either vehicle or metformin once a day for 12 weeks. Epididymal adipose tissue was dissected from the mice after an overnight fast, snap-frozen in liquid nitrogen, then stored at −75 °C until analysis. Total RNA was extracted from epididymal adipose tissue of the RD, HFD, and MET groups using an RNeasy Mini kit (Qiagen, Hilden, Germany) according to the manufacturer’s instructions.

### Microarray and network analysis

The purity and integrity of total RNA extracted from epididymal adipose tissue were evaluated by the ratio of absorbance at 260 and 280 nm and by using an Agilent 2100 bioanalyzer (Agilent Technologies, Palo Alto, CA, USA), respectively. The RNA samples from each group were subjected to global gene expression analysis using Affymetrix GeneChip Mouse Gene 2.0 ST arrays (Affymetrix, Santa Clara, CA, USA). The raw and processed microarray data reported in this analysis is deposited in the GEO database as GSE102540. The microarray datasets were preprocessed and normalized using the RMA method. A biological protein-protein interaction (PPI) network was constructed by NetworkAnalyst (http://www.networkanalyst.ca/)^22,23^ using the Z-scores of our meta-signature and the interactome database from InnateDB^24^.

### Quantitative PCR (qPCR)

A total of 2 µg of RNA was reversely transcribed using M-MLV reverse transcriptase (Promega, Madison, WI, USA) and oligo (dT) primers as suggested by the manufacturer. The resulting cDNA was used as a template for qPCR analysis using SYBR Green PCR Master Mix (Applied Biosystems, Warrington, UK) with StepOnePlus Real-Time PCR (Applied Biosystems, Foster City, CA, USA). The oligonucleotide primer sequences used in qPCR analysis were as follows: *S100a4* forward, 5′-TCCACAAATACTCAGGCAAAGAG-3′ and reverse, 5′-GCAGCTCCCTGGTCAGTAG-3′; *P2rx7* forward, 5′-GACAAACAAAGTCACCCGGAT-3′ and reverse, 5′-CGCTCACCAAAGCAAAGCTAAT-3′; *Psap* forward, 5′-TGCTGAAAGATAATGCTACGCA-3′ and reverse, 5′-GCAGGTAAGAGTCAACCACCTC-3′; *Grn* forward, 5′-GGACACATGGCCTAGAATAACG-3′ and reverse, 5′-AGACACACCCTTAGAGAACGG-3′; *S100a1* forward, 5′-AATGTGTTCCATGCCCATTCG-3′ and reverse, 5′-ACCAGCACAACATACTCCTTG-3′; *Cyb5a* forward, 5′-TGATGCTACCGAGAATTTTGAGG-3′ and reverse, 5′-GGAGTTCCCCGATGATGTATGT-3′; *Pdk2* forward, 5′-AGGGGCACCCAAGTACATC-3′ and reverse, 5′-TGCCGGAGGAAAGTGAATGAC-3′; *Mccc1* forward, 5′-ACCATGAAGTATGGAACAACCC-3′ and reverse, 5′-TGCACACCCATCTTTTTGGCT-3′. The temperature profile of the reaction was 95 °C for 10 min followed by 40 cycles of denaturation at 95 °C for 15 s and annealing/extension at 60 °C for 1 min. All reactions were performed in triplicate. The relative gene expression levels of the target genes were normalized to the expression levels of *β*-actin and the fold change in expression was calculated using the 2^(−ΔΔCt)^ method.

## Electronic supplementary material

Supplementary Information
