# Supplementary material for: Meta- and cross-species analyses of insulin resistance based on gene expression datasets in human white adipose tissues
Source: Sci Rep. 2018 Feb 27;8:3747. doi: 10.1038/s41598-017-18082-7 (PMC5829071; doi:10.1038/s41598-017-18082-7)
Supplement: Supplementary file 1 — Supplementary Information [file 41598_2017_18082_MOESM1_ESM.pdf]

Supplementary Information

Meta- and cross-species analyses of insulin resistance based on gene expression datasets in human white adipose tissues

Junghyun Jung[1], Go Woon Kim[2], Woosuk Lee[1], Changsoo Mok[1], Sung Hyun Chung[2], Wonhee Jang[1*]

1. *Department of Life Science, Dongguk University, Pildong 3-ga, Seoul, Korea 04620*
2. *Department of Pharmacology, College of Pharmacy, Kyung Hee University, 26 Kyungheedae-ro, Seoul, Korea 02447*

Figure S1. An overview of the analysis workflow.

Figure S2. Functional enrichment analysis using GSEA.

Figure S3. Correlation analysis of BMI, HOMA2, and Z-scores of drug signature.

Figure S4. Z-scores of each study and the meta-analysis.

Table S1. The identified human IR meta-signature.

Table S2. The identified cross-species drug signature.

Figure S1. An overview of the analysis workflow.

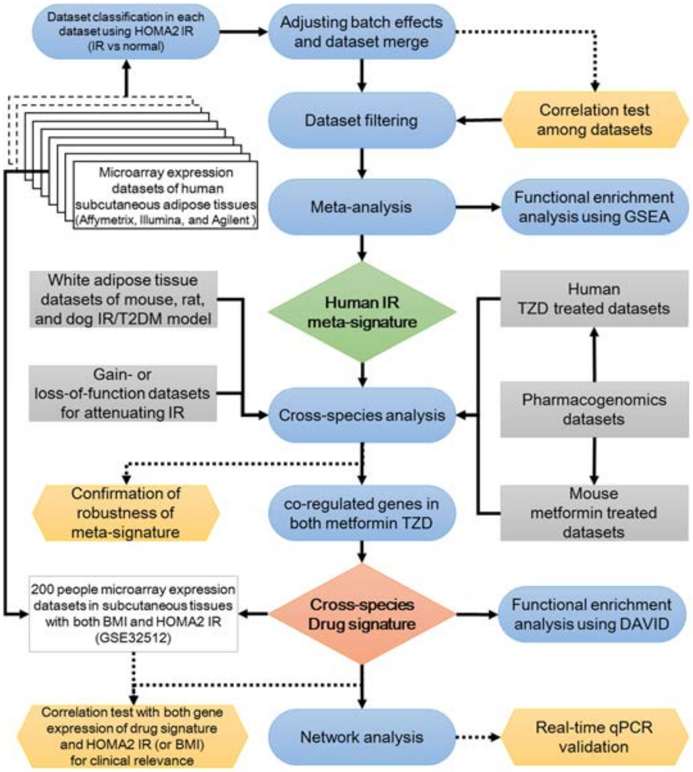

The scheme of meta-analysis and cross-species analysis procedures used to identify robust genetic markers and potential therapeutic target genes involved in IR are shown. The blank boxes denote the microarray datasets used for meta-analysis in human subcutaneous adipose tissue except for two microarray datasets represented by the dotted blank box that were removed from the analysis. The blue eclipses denote the analysis steps and the diamonds indicate signature genes involved in insulin resistance and potential therapeutic targets. The grey boxes indicate other expression microarray datasets used to confirm the robustness of human IR meta-signature and to identify cross-species drug signature, and the solid arrows indicate the direction of analysis. The yellow hexagons denote the validation step and the dashed arrow lines represent the direction of the validation step.

Figure S2. Functional enrichment analysis using GSEA.

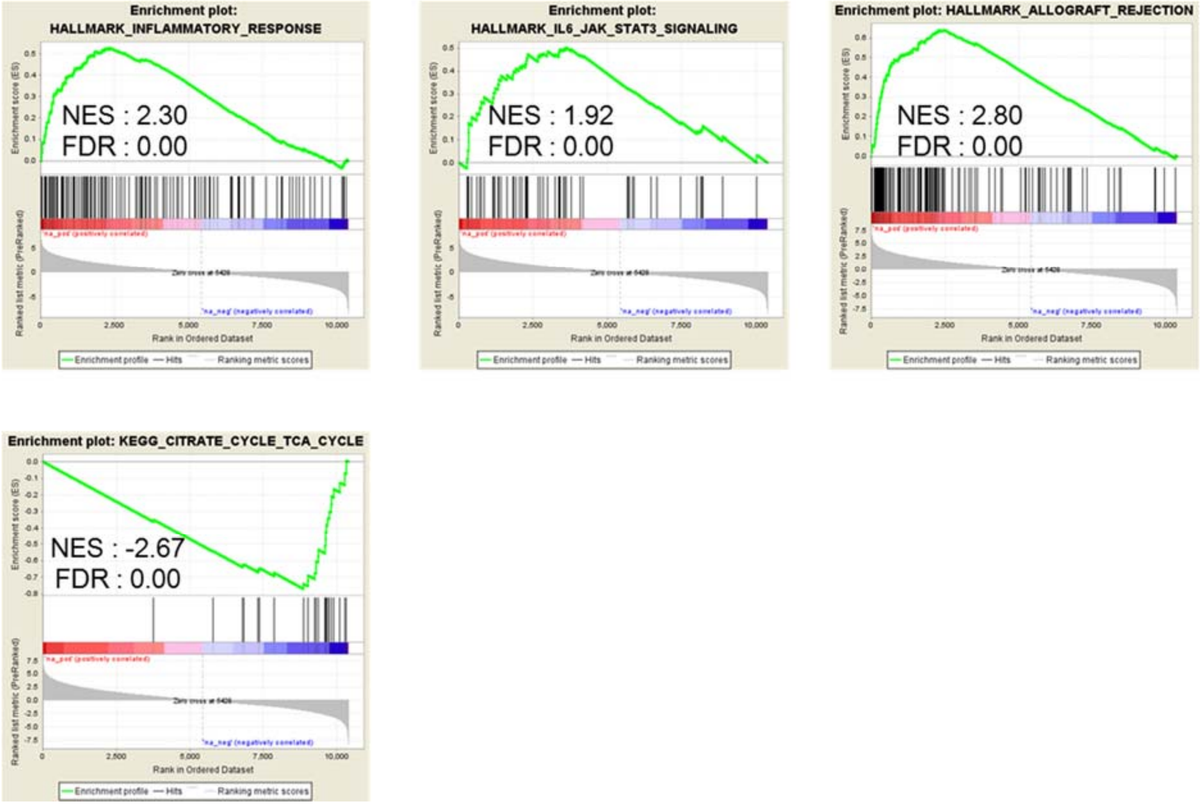

GSEA results obtained using meta-analysis Z-scores and both KEGG and MSigDB hallmark gene sets.

Figure S3. Correlation analysis of BMI, HOMA2, and Z-scores of drug signature.

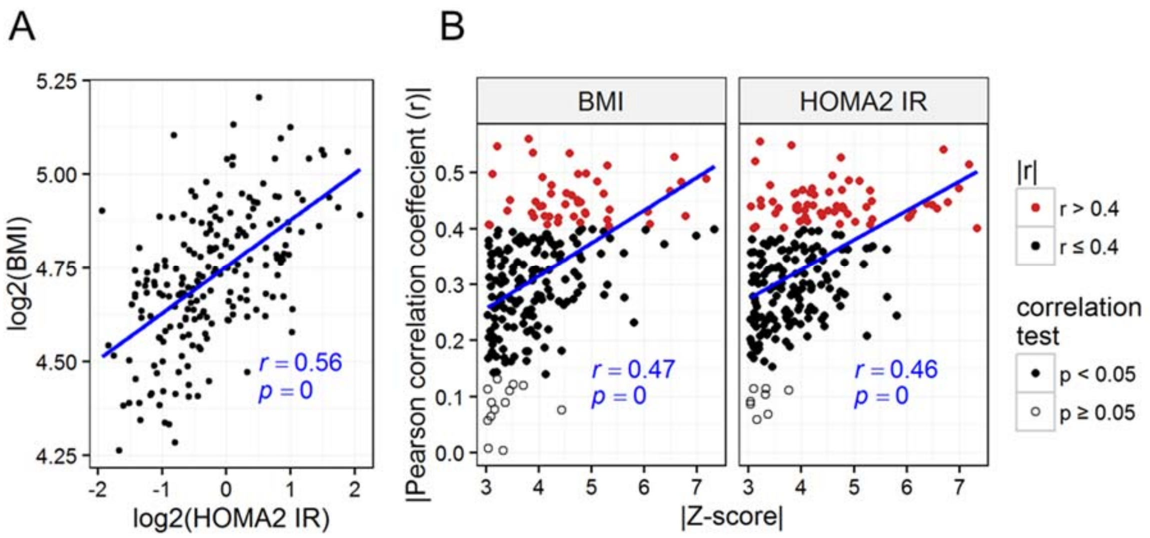

(A) Correlation analysis between HOMA2-IR and BMI based on 200 subjects using the GSE32512 datasets. (B) Correlation analysis of BMI, HOMA2-IR, and Z-scores. The y-axis shows the absolute value of the correlation coefficient between the expression levels of 211 drug signature and HOMA2-IR or BMI. The x-axis shows the absolute value of the 211 drug signature's Z-scores.

Figure S4. Z-scores of each study and the meta-analysis.

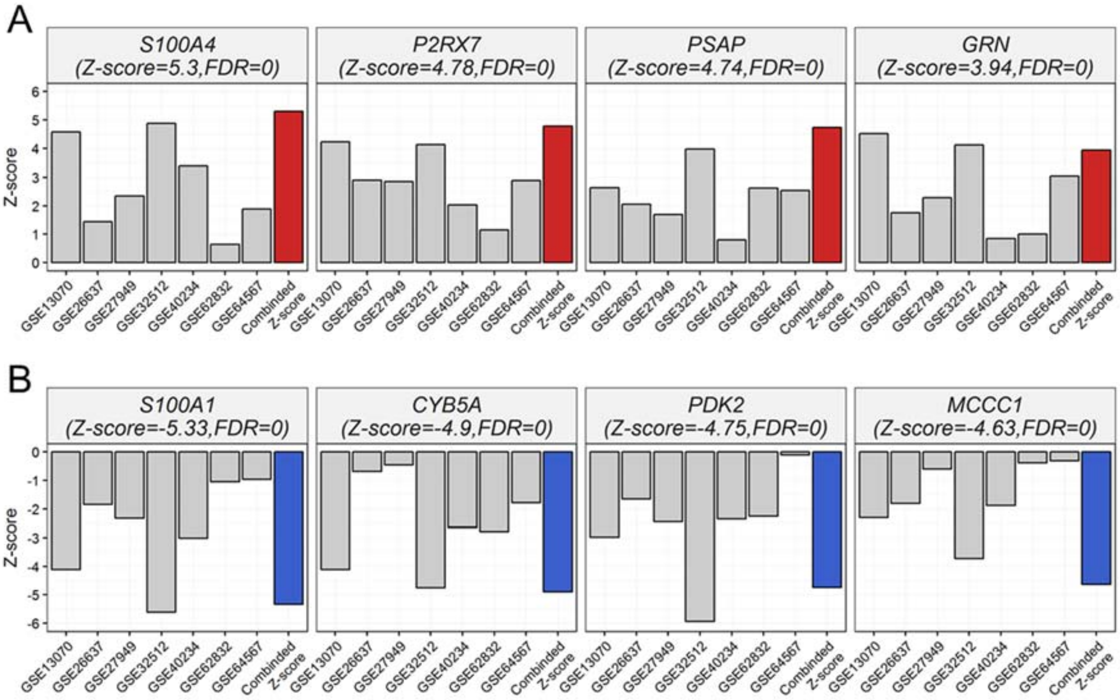

(A) Up- and (B) down-regulated drug signature's Z-scores in each study and in the meta-analysis for genes identified by network analysis (Fig. 8A).

Table S1. The identified human IR meta-signatures.

Gene id	Symbol	Gene name	Z-score	FDR
7422	VEGFA	vascular endothelial growth factor A	-8.34	0.00.E+00
8714	ABCC3	ATP binding cassette subfamily C member 3	7.89	0.00.E+00
1890	TYMP	thymidine phosphorylase	7.85	0.00.E+00
23171	GPD1L	glycerol-3-phosphate dehydrogenase 1-like	-7.80	0.00.E+00
152007	GLIPR2	GLI pathogenesis related 2	7.52	0.00.E+00
10157	AASS	aminoadipate-semialdehyde synthase	-7.41	0.00.E+00
8870	IER3	immediate early response 3	7.33	0.00.E+00
338	APOB	apolipoprotein B	-7.25	0.00.E+00
894	CCND2	cyclin D2	7.18	0.00.E+00
1979	EIF4EBP2	eukaryotic translation initiation factor 4E binding protein 2	-7.18	0.00.E+00
563	AZGP1	alpha-2-glycoprotein 1, zinc-binding	-7.16	0.00.E+00
7291	TWIST1	twist family bHLH transcription factor 1	-7.13	0.00.E+00
347733	TUBB2B	tubulin beta 2B class IIb	7.13	0.00.E+00
6678	SPARC	secreted protein acidic and cysteine rich	7.05	0.00.E+00
11043	MID2	midline 2	-6.99	0.00.E+00
1175	AP2S1	adaptor related protein complex 2 sigma 1 subunit	6.81	0.00.E+00
80763	SPX	spexin hormone	-6.80	0.00.E+00
56301	SLC7A10	solute carrier family 7 member 10	-6.78	0.00.E+00
4337	MOCS1	molybdenum cofactor synthesis 1	-6.70	0.00.E+00
83861	RSPH3	radial spoke 3 homolog	6.70	0.00.E+00
7100	TLR5	toll like receptor 5	6.69	0.00.E+00
2752	GLUL	glutamate-ammonia ligase	-6.67	0.00.E+00
84868	HAVCR2	hepatitis A virus cellular receptor 2	6.65	0.00.E+00
51660	MPC1	mitochondrial pyruvate carrier 1	-6.59	0.00.E+00
8682	PEA15	phosphoprotein enriched in astrocytes 15	6.58	0.00.E+00
25975	EGFL6	EGF like domain multiple 6	6.58	0.00.E+00
8644	AKR1C3	aldo-keto reductase family 1 member C3	6.57	0.00.E+00
3075	CFH	complement factor H	6.55	0.00.E+00
23258	DENND5A	DENN domain containing 5A	6.53	0.00.E+00
80301	PLEKHO2	pleckstrin homology domain containing O2	6.50	0.00.E+00
3952	LEP	leptin	6.49	0.00.E+00
64756	ATPAF1	ATP synthase mitochondrial F1 complex assembly factor 1	-6.38	0.00.E+00
340348	TSPAN33	tetraspanin 33	6.35	0.00.E+00
8777	MPDZ	multiple PDZ domain crumbs cell polarity complex component	-6.32	0.00.E+00
813	CALU	calumenin	6.29	0.00.E+00
1536	CYBB	cytochrome b-245 beta chain	6.28	0.00.E+00
390	RND3	Rho family GTPase 3	6.12	0.00.E+00
3929	LBP	lipopolysaccharide binding protein	6.11	0.00.E+00
1475	CSTA	cystatin A	6.10	0.00.E+00
2650	GCNT1	glucosaminyl (N-acetyl) transferase 1, core 2	6.07	0.00.E+00
57698	SHTN1	shootin 1	6.06	0.00.E+00
5216	PFN1	profilin 1	6.03	0.00.E+00
201161	CENPV	centromere protein V	-6.02	0.00.E+00
114548	NLRP3	NLR family pyrin domain containing 3	6.01	0.00.E+00
3033	HADH	hydroxyacyl-CoA dehydrogenase	-5.99	0.00.E+00
10655	DMRT2	doublesex and mab-3 related transcription factor 2	-5.98	0.00.E+00
151306	GPBAR1	G protein-coupled bile acid receptor 1	5.95	0.00.E+00
51222	ZNF219	zinc finger protein 219	5.94	0.00.E+00
4145	MATK	megakaryocyte-associated tyrosine kinase	5.93	0.00.E+00
1522	CTSZ	cathepsin Z	5.90	0.00.E+00
3693	ITGB5	integrin subunit beta 5	5.88	0.00.E+00
6309	SC5D	sterol-C5-desaturase	-5.88	0.00.E+00
54732	TMED9	transmembrane p24 trafficking protein 9	5.86	0.00.E+00
255631	COL24A1	collagen type XXIV alpha 1 chain	5.86	0.00.E+00
977	CD151	CD151 molecule (Raph blood group)	5.85	0.00.E+00
4883	NPR3	natriuretic peptide receptor 3	5.85	0.00.E+00
284021	MILR1	mast cell immunoglobulin like receptor 1	5.85	0.00.E+00
55787	TXLNG	taxilin gamma	-5.84	0.00.E+00
7108	TM7SF2	transmembrane 7 superfamily member 2	-5.84	0.00.E+00
55283	MCOLN3	mucolipin 3	-5.82	0.00.E+00
9051	PSTPIP1	proline-serine-threonine phosphatase interacting protein 1	5.81	0.00.E+00
54976	C20orf27	chromosome 20 open reading frame 27	-5.79	0.00.E+00
87	ACTN1	actinin alpha 1	5.77	0.00.E+00
7385	UQCRC2	ubiquinol-cytochrome c reductase core protein II	-5.77	0.00.E+00
59338	PLEKHA1	pleckstrin homology domain containing A1	5.76	0.00.E+00

85477	SCIN	scinderin	5.75	0.00.E+00
2675	GFRA2	GDNF family receptor alpha 2	5.75	0.00.E+00
7739	ZNF185	zinc finger protein 185 (LIM domain)	5.74	0.00.E+00
549	AUH	AU RNA binding methylglutaconyl-CoA hydratase	-5.74	0.00.E+00
8204	NRIP1	nuclear receptor interacting protein 1	-5.71	0.00.E+00
5092	PCBD1	pterin-4 alpha-carbinolamine dehydratase 1	-5.70	0.00.E+00
5774	PTPN3	protein tyrosine phosphatase, non-receptor type 3	-5.70	0.00.E+00
115416	MALSU1	mitochondrial assembly of ribosomal large subunit 1	-5.68	0.00.E+00
11006	LILRB4	leukocyte immunoglobulin like receptor B4	5.68	0.00.E+00
51313	FAM198B	family with sequence similarity 198 member B	5.65	0.00.E+00
1026	CDKN1A	cyclin dependent kinase inhibitor 1A	5.64	0.00.E+00
3309	HSPA5	heat shock protein family A (Hsp70) member 5	5.63	0.00.E+00
91461	PKDCC	protein kinase domain containing, cytoplasmic	-5.63	0.00.E+00
3791	KDR	kinase insert domain receptor	-5.63	0.00.E+00
1959	EGR2	early growth response 2	5.62	0.00.E+00
6623	SNCG	synuclein gamma	5.60	0.00.E+00
1880	GPR183	G protein-coupled receptor 183	5.60	0.00.E+00
5707	PSMD1	proteasome 26S subunit, non-ATPase 1	5.60	0.00.E+00
94103	ORMDL3	ORMDL sphingolipid biosynthesis regulator 3	-5.58	0.00.E+00
2028	ENPEP	glutamyl aminopeptidase	-5.58	0.00.E+00
27147	DENND2A	DENN domain containing 2A	-5.57	0.00.E+00
9469	CHST3	carbohydrate sulfotransferase 3	5.57	0.00.E+00
3667	IRS1	insulin receptor substrate 1	-5.53	0.00.E+00
9194	SLC16A7	solute carrier family 16 member 7	-5.51	0.00.E+00
7345	UCHL1	ubiquitin C-terminal hydrolase L1	5.51	0.00.E+00
28973	MRPS18B	mitochondrial ribosomal protein S18B	-5.50	0.00.E+00
54463	FAM134B	family with sequence similarity 134 member B	-5.50	0.00.E+00
9956	HS3ST2	heparan sulfate-glucosamine 3-sulfotransferase 2	5.50	0.00.E+00
408	ARRB1	arrestin beta 1	-5.49	0.00.E+00
284	ANGPT1	angiopoietin 1	5.47	0.00.E+00
54958	TMEM160	transmembrane protein 160	5.47	0.00.E+00
34	ACADM	acyl-CoA dehydrogenase, C-4 to C-12 straight chain	-5.46	0.00.E+00
9326	ZNHIT3	zinc finger HIT-type containing 3	-5.45	0.00.E+00
29992	PILRA	paired immunoglobin like type 2 receptor alpha	5.44	0.00.E+00
7037	TFRC	transferrin receptor	5.43	0.00.E+00
51726	DNAJB11	DnaJ heat shock protein family (Hsp40) member B11	5.40	0.00.E+00
3914	LAMB3	laminin subunit beta 3	5.40	0.00.E+00
113675	SDSL	serine dehydratase like	5.39	0.00.E+00
6920	TCEA3	transcription elongation factor A3	-5.38	0.00.E+00
5996	RGS1	regulator of G-protein signaling 1	5.36	0.00.E+00
595	CCND1	cyclin D1	5.36	0.00.E+00
10783	NEK6	NIMA related kinase 6	5.36	0.00.E+00
57552	NCEH1	neutral cholesterol ester hydrolase 1	5.35	0.00.E+00
316	AOX1	aldehyde oxidase 1	5.35	0.00.E+00
83943	IMMP2L	inner mitochondrial membrane peptidase subunit 2	-5.35	0.00.E+00
116843	SLC18B1	solute carrier family 18 member B1	5.34	0.00.E+00
10272	FSTL3	follistatin like 3	5.34	0.00.E+00
6271	S100A1	S100 calcium binding protein A1	-5.33	0.00.E+00
27287	VENTX	VENT homeobox	5.32	0.00.E+00
8444	DYRK3	dual specificity tyrosine phosphorylation regulated kinase 3	-5.32	0.00.E+00
128553	TSHZ2	teashirt zinc finger homeobox 2	5.32	0.00.E+00
7481	WNT11	Wnt family member 11	-5.31	0.00.E+00
2872	MKNK2	MAP kinase interacting serine/threonine kinase 2	-5.31	0.00.E+00
3704	ITPA	inosine triphosphatase	5.30	0.00.E+00
6275	S100A4	S100 calcium binding protein A4	5.30	0.00.E+00
10533	ATG7	autophagy related 7	5.29	0.00.E+00
134266	GRPEL2	GrpE like 2, mitochondrial	-5.29	0.00.E+00
2817	GPC1	glypican 1	5.29	0.00.E+00
1601	DAB2	DAB2, clathrin adaptor protein	5.27	8.06.E-06
64102	TNMD	tenomodulin	5.26	8.00.E-06
153579	BTNL9	butyrophilin like 9	-5.25	7.94.E-06
4329	ALDH6A1	aldehyde dehydrogenase 6 family member A1	-5.23	7.87.E-06
28999	KLF15	Kruppel like factor 15	-5.23	7.81.E-06
393	ARHGAP4	Rho GTPase activating protein 4	5.21	7.75.E-06
57124	CD248	CD248 molecule	5.21	7.69.E-06
54602	NDFIP2	Nedd4 family interacting protein 2	-5.20	7.63.E-06
8495	PPFIBP2	PPFIA binding protein 2	5.19	7.58.E-06

79709	COLGALT1	collagen beta(1-O)galactosyltransferase 1	5.19	7.52.E-06
594	BCKDHB	branched chain keto acid dehydrogenase E1 subunit beta	-5.18	7.46.E-06
23022	PALLD	palladin, cytoskeletal associated protein	5.18	7.41.E-06
9833	MELK	maternal embryonic leucine zipper kinase	5.18	7.35.E-06
35	ACADS	acyl-CoA dehydrogenase, C-2 to C-3 short chain	-5.15	7.30.E-06
27350	APOBEC3C	apolipoprotein B mRNA editing enzyme catalytic subunit 3C	5.15	7.25.E-06
2119	ETV5	ETS variant 5	5.15	7.19.E-06
3003	GZMK	granzyme K	5.12	7.14.E-06
54665	RSBN1	round spermatid basic protein 1	-5.11	1.42.E-05
6448	SGSH	N-sulfoglucosamine sulfohydrolase	5.11	1.41.E-05
5827	PXMP2	peroxisomal membrane protein 2	-5.11	1.40.E-05
90768	LOC90768	uncharacterized LOC90768	-5.11	1.39.E-05
1845	DUSP3	dual specificity phosphatase 3	5.11	1.38.E-05
83606	GUCD1	guanylyl cyclase domain containing 1	-5.10	1.37.E-05
3687	ITGAX	integrin subunit alpha X	5.09	1.36.E-05
10003	NAALAD2	N-acetylated alpha-linked acidic dipeptidase 2	-5.08	1.35.E-05
5715	PSMD9	proteasome 26S subunit, non-ATPase 9	5.08	1.34.E-05
201164	PLD6	phospholipase D family member 6	-5.05	1.33.E-05
9242	MSC	musculin	5.05	1.32.E-05
196740	VSTM4	V-set and transmembrane domain containing 4	-5.05	1.32.E-05
8832	CD84	CD84 molecule	5.05	1.31.E-05
25805	BAMBI	BMP and activin membrane bound inhibitor	5.04	1.30.E-05
56997	COQ8A	coenzyme Q8A	-5.04	1.29.E-05
4982	TNFRSF11B	TNF receptor superfamily member 11b	5.03	1.28.E-05
716	C1S	complement C1s	5.03	1.27.E-05
7941	PLA2G7	phospholipase A2 group VII	5.02	1.27.E-05
23507	LRRC8B	leucine rich repeat containing 8 family member B	-5.01	1.26.E-05
27128	CYTH4	cytohesin 4	4.99	2.50.E-05
8801	SUCLG2	succinate-CoA ligase GDP-forming beta subunit	-4.98	2.48.E-05
29091	STXBP6	syntaxin binding protein 6	-4.98	2.47.E-05
64420	SUSD1	sushi domain containing 1	4.97	2.45.E-05
9500	MAGED1	MAGE family member D1	4.97	2.44.E-05
80760	ITIH5	inter-alpha-trypsin inhibitor heavy chain family member 5	4.96	3.64.E-05
114804	RNF157	ring finger protein 157	-4.96	3.61.E-05
10269	ZMPSTE24	zinc metallopeptidase STE24	4.96	3.59.E-05
432	ASGR1	asialoglycoprotein receptor 1	4.95	3.57.E-05
945	CD33	CD33 molecule	4.94	4.14.E-05
254428	SLC41A1	solute carrier family 41 member 1	-4.93	4.12.E-05
55183	RIF1	replication timing regulatory factor 1	-4.93	4.09.E-05
2535	FZD2	frizzled class receptor 2	4.93	4.07.E-05
23423	TMED3	transmembrane p24 trafficking protein 3	4.93	4.05.E-05
9353	SLIT2	slit guidance ligand 2	4.92	4.02.E-05
54539	NDUFB11	NADH:ubiquinone oxidoreductase subunit B11	-4.92	4.57.E-05
11031	RAB31	RAB31, member RAS oncogene family	4.92	4.55.E-05
4702	NDUFA8	NADH:ubiquinone oxidoreductase subunit A8	-4.91	4.52.E-05
5737	PTGFR	prostaglandin F receptor	4.91	4.49.E-05
23753	SDF2L1	stromal cell derived factor 2 like 1	4.91	4.47.E-05
81552	VOPP1	vesicular, overexpressed in cancer, prosurvival protein 1	4.91	4.44.E-05
57419	SLC24A3	solute carrier family 24 member 3	4.91	4.42.E-05
152189	CMTM8	CKLF like MARVEL transmembrane domain containing 8	-4.91	4.40.E-05
10203	CALCRL	calcitonin receptor like receptor	-4.91	4.37.E-05
151887	CCDC80	coiled-coil domain containing 80	4.91	4.35.E-05
7763	ZFAND5	zinc finger AN1-type containing 5	-4.91	4.32.E-05
1528	CYB5A	cytochrome b5 type A	-4.90	4.84.E-05
308	ANXA5	annexin A5	4.90	5.35.E-05
925	CD8A	CD8a molecule	4.90	5.32.E-05
314	AOC2	amine oxidase, copper containing 2	4.90	5.29.E-05
3685	ITGAV	integrin subunit alpha V	4.89	5.26.E-05
940	CD28	CD28 molecule	4.89	5.24.E-05
157506	RDH10	retinol dehydrogenase 10 (all-trans)	-4.89	5.21.E-05
5973	RENBP	renin binding protein	4.89	5.18.E-05
10170	DHRS9	dehydrogenase/reductase 9	4.88	5.15.E-05
285362	SUMF1	sulfatase modifying factor 1	4.86	6.15.E-05
158326	FREM1	FRAS1 related extracellular matrix 1	4.86	6.12.E-05
3705	ITPK1	inositol-tetrakisphosphate 1-kinase	4.86	6.09.E-05
9170	LPAR2	lysophosphatidic acid receptor 2	4.86	6.06.E-05
283078	MKX	mohawk homeobox	-4.86	6.03.E-05

5947	RBP1	retinol binding protein 1	-4.85	6.00.E-05
126014	OSCAR	osteoclast associated, immunoglobulin-like receptor	4.85	6.47.E-05
58504	ARHGAP22	Rho GTPase activating protein 22	4.85	6.93.E-05
56892	C8orf4	chromosome 8 open reading frame 4	-4.84	7.39.E-05
1062	CENPE	centromere protein E	4.83	7.35.E-05
4711	NDUFB5	NADH:ubiquinone oxidoreductase subunit B5	-4.83	7.80.E-05
2589	GALNT1	polypeptide N-acetylgalactosaminyltransferase 1	4.83	7.77.E-05
83706	FERMT3	fermitin family member 3	4.83	7.73.E-05
5768	QSOX1	quiescin sulfhydryl oxidase 1	4.83	7.69.E-05
2802	GOLGA3	golgin A3	4.83	7.66.E-05
57520	HECW2	HECT, C2 and WW domain containing E3 ubiquitin protein ligase 2	-4.82	7.62.E-05
2769	GNA15	G protein subunit alpha 15	4.82	7.58.E-05
219855	SLC37A2	solute carrier family 37 member 2	4.81	8.02.E-05
56937	PMEPA1	prostate transmembrane protein, androgen induced 1	4.81	7.98.E-05
1292	COL6A2	collagen type VI alpha 2 chain	4.81	7.94.E-05
388135	C15orf59	chromosome 15 open reading frame 59	4.80	7.91.E-05
84706	GPT2	glutamic--pyruvic transaminase 2	-4.80	7.87.E-05
5027	P2RX7	purinergic receptor P2X 7	4.78	8.29.E-05
301	ANXA1	annexin A1	4.78	8.26.E-05
64785	GINS3	GINS complex subunit 3	-4.78	8.22.E-05
2876	GPX1	glutathione peroxidase 1	4.77	8.18.E-05
8800	PEX11A	peroxisomal biogenesis factor 11 alpha	-4.77	8.14.E-05
38	ACAT1	acetyl-CoA acetyltransferase 1	-4.77	8.11.E-05
1728	NQO1	NAD(P)H quinone dehydrogenase 1	4.77	8.07.E-05
5920	RARRES3	retinoic acid receptor responder 3	4.76	8.04.E-05
11332	ACOT7	acyl-CoA thioesterase 7	4.76	8.00.E-05
9781	RNF144A	ring finger protein 144A	-4.75	8.85.E-05
55690	PACS1	phosphofurin acidic cluster sorting protein 1	4.75	9.25.E-05
23175	LPIN1	lipin 1	-4.75	9.21.E-05
5164	PDK2	pyruvate dehydrogenase kinase 2	-4.75	9.17.E-05
2213	FCGR2B	Fc fragment of IgG receptor IIb	4.74	9.13.E-05
9710	KIAA0355	KIAA0355	-4.74	9.09.E-05
10159	ATP6AP2	ATPase H+ transporting accessory protein 2	4.74	9.05.E-05
1476	CSTB	cystatin B	4.74	9.44.E-05
5660	PSAP	prosaposin	4.74	9.40.E-05
6129	RPL7	ribosomal protein L7	-4.74	1.02.E-04
1066	CES1	carboxylesterase 1	4.73	1.02.E-04
914	CD2	CD2 molecule	4.73	1.01.E-04
80727	TTYH3	tweety family member 3	4.73	1.05.E-04
51299	NRN1	neuritin 1	-4.71	1.21.E-04
1102	RCBTB2	RCC1 and BTB domain containing protein 2	4.70	1.21.E-04
8876	VNN1	vanin 1	4.70	1.24.E-04
6709	SPTAN1	spectrin alpha, non-erythrocytic 1	4.69	1.24.E-04
51227	PIGP	phosphatidylinositol glycan anchor biosynthesis class P	-4.69	1.23.E-04
10144	FAM13A	family with sequence similarity 13 member A	-4.69	1.23.E-04
222553	SLC35F1	solute carrier family 35 member F1	-4.68	1.39.E-04
4131	MAP1B	microtubule associated protein 1B	4.67	1.38.E-04
85457	CIPC	CLOCK interacting pacemaker	-4.67	1.38.E-04
10184	LHFPL2	lipoma HMGIC fusion partner-like 2	4.67	1.41.E-04
22850	ADNP2	ADNP homeobox 2	-4.67	1.45.E-04
713	C1QB	complement C1q B chain	4.67	1.48.E-04
55902	ACSS2	acyl-CoA synthetase short-chain family member 2	-4.66	1.47.E-04
128977	C22orf39	chromosome 22 open reading frame 39	-4.66	1.47.E-04
56833	SLAMF8	SLAM family member 8	4.66	1.46.E-04
51015	ISOC1	isochorismatase domain containing 1	-4.66	1.46.E-04
23654	PLXNB2	plexin B2	4.66	1.45.E-04
282973	JAKMIP3	Janus kinase and microtubule interacting protein 3	4.66	1.45.E-04
4070	TACSTD2	tumor-associated calcium signal transducer 2	4.66	1.44.E-04
339390	CLEC4G	C-type lectin domain family 4 member G	4.66	1.43.E-04
4015	LOX	lysyl oxidase	4.65	1.47.E-04
8767	RIPK2	receptor interacting serine/threonine kinase 2	4.65	1.46.E-04
57121	LPAR5	lysophosphatidic acid receptor 5	4.65	1.46.E-04
25878	MXRA5	matrix remodeling associated 5	4.65	1.45.E-04
124	ADH1A	alcohol dehydrogenase 1A (class I), alpha polypeptide	-4.65	1.44.E-04
23417	MLYCD	malonyl-CoA decarboxylase	-4.63	1.52.E-04
8614	STC2	stanniocalcin 2	4.63	1.51.E-04
56922	MCCC1	methylcrotonoyl-CoA carboxylase 1	-4.63	1.50.E-04

202018	TAPT1	transmembrane anterior posterior transformation 1	-4.62	1.54.E-04
4277	MICB	MHC class I polypeptide-related sequence B	4.62	1.57.E-04
64423	INF2	inverted formin, FH2 and WH2 domain containing	4.62	1.56.E-04
92162	TMEM88	transmembrane protein 88	-4.61	1.59.E-04
5099	PCDH7	protocadherin 7	4.61	1.59.E-04
29927	SEC61A1	Sec61 translocon alpha 1 subunit	4.61	1.58.E-04
8455	ATRN	attractin	4.61	1.58.E-04
6403	SELP	selectin P	4.60	1.64.E-04
4318	MMP9	matrix metallopeptidase 9	4.60	1.64.E-04
10402	ST3GAL6	ST3 beta-galactoside alpha-2,3-sialyltransferase 6	-4.60	1.63.E-04
79095	C9orf16	chromosome 9 open reading frame 16	4.60	1.62.E-04
3600	IL15	interleukin 15	4.60	1.62.E-04
2132	EXT2	exostosin glycosyltransferase 2	4.59	1.68.E-04
5209	PFKFB3	6-phosphofructo-2-kinase/fructose-2,6-biphosphatase 3	-4.59	1.68.E-04
4035	LRP1	LDL receptor related protein 1	4.58	1.67.E-04
864	RUNX3	runt related transcription factor 3	4.58	1.67.E-04
6799	SULT1A2	sulfotransferase family 1A member 2	4.58	1.66.E-04
58526	MID1IP1	MID1 interacting protein 1	-4.58	1.73.E-04
29763	PACSIN3	protein kinase C and casein kinase substrate in neurons 3	-4.57	1.72.E-04
80273	GRPEL1	GrpE like 1, mitochondrial	-4.57	1.71.E-04
257106	ARHGAP30	Rho GTPase activating protein 30	4.56	1.74.E-04
2108	ETFA	electron transfer flavoprotein alpha subunit	-4.56	1.77.E-04
1975	EIF4B	eukaryotic translation initiation factor 4B	-4.55	1.83.E-04
83937	RASSF4	Ras association domain family member 4	4.54	1.83.E-04
8942	KYNU	kynureninase	4.54	1.82.E-04
28514	DLL1	delta like canonical Notch ligand 1	-4.54	1.82.E-04
3936	LCP1	lymphocyte cytosolic protein 1	4.53	1.81.E-04
55638	SYBU	syntabulin	-4.53	1.80.E-04
54	ACP5	acid phosphatase 5, tartrate resistant	4.53	1.80.E-04
11326	VSIG4	V-set and immunoglobulin domain containing 4	4.52	1.79.E-04
6609	SMPD1	sphingomyelin phosphodiesterase 1	4.52	1.78.E-04
126321	MFSD12	major facilitator superfamily domain containing 12	4.52	1.78.E-04
9093	DNAJA3	DnaJ heat shock protein family (Hsp40) member A3	-4.52	1.84.E-04
6184	RPN1	ribophorin I	4.52	1.87.E-04
9804	TOMM20	translocase of outer mitochondrial membrane 20	-4.52	1.86.E-04
7342	UBP1	upstream binding protein 1 (LBP-1a)	4.52	1.85.E-04
23307	FKBP15	FK506 binding protein 15	4.51	1.85.E-04
56945	MRPS22	mitochondrial ribosomal protein S22	-4.51	1.84.E-04
714	C1QC	complement C1q C chain	4.51	1.90.E-04
11075	STMN2	stathmin 2	4.51	1.90.E-04
54432	YIPF1	Yip1 domain family member 1	4.50	1.89.E-04
12	SERPINA3	serpin family A member 3	4.50	1.88.E-04
6509	SLC1A4	solute carrier family 1 member 4	4.50	1.91.E-04
23460	ABCA6	ATP binding cassette subfamily A member 6	4.50	1.90.E-04
28992	MACROD1	MACRO domain containing 1	-4.50	1.90.E-04
5160	PDHA1	pyruvate dehydrogenase (lipoamide) alpha 1	-4.49	1.92.E-04
84215	ZNF541	zinc finger protein 541	4.48	1.95.E-04
2350	FOLR2	folate receptor beta	4.48	1.94.E-04
1650	DDOST	dolichyl-diphosphooligosaccharide--protein glycosyltransferase non-catalytic subunit	4.48	1.97.E-04
11184	MAP4K1	mitogen-activated protein kinase kinase kinase kinase 1	4.47	1.96.E-04
5179	PENK	proenkephalin	4.47	1.96.E-04
3646	EIF3E	eukaryotic translation initiation factor 3 subunit E	-4.47	1.98.E-04
5330	PLCB2	phospholipase C beta 2	4.46	2.10.E-04
85460	ZNF518B	zinc finger protein 518B	-4.46	2.09.E-04
5067	CNTN3	contactin 3	4.46	2.09.E-04
7436	VLDLR	very low density lipoprotein receptor	4.46	2.08.E-04
6146	RPL22	ribosomal protein L22	-4.46	2.07.E-04
51455	REV1	REV1, DNA directed polymerase	-4.46	2.07.E-04
3140	MR1	major histocompatibility complex, class I-related	4.46	2.06.E-04
28984	RGCC	regulator of cell cycle	-4.45	2.12.E-04
55203	LGI2	leucine rich repeat LGI family member 2	4.44	2.14.E-04
1410	CRYAB	crystallin alpha B	4.44	2.13.E-04
84263	HSDL2	hydroxysteroid dehydrogenase like 2	-4.44	2.13.E-04
2331	FMOD	fibromodulin	4.43	2.12.E-04
6357	CCL13	C-C motif chemokine ligand 13	4.42	2.24.E-04
9961	MVP	major vault protein	4.42	2.23.E-04
5025	P2RX4	purinergic receptor P2X 4	4.41	2.22.E-04

1874	E2F4	E2F transcription factor 4	-4.41	2.22.E-04
55758	RCOR3	REST corepressor 3	-4.41	2.24.E-04
201266	SLC39A11	solute carrier family 39 member 11	4.41	2.26.E-04
54996	MARC2	mitochondrial amidoxime reducing component 2	-4.41	2.26.E-04
4481	MSR1	macrophage scavenger receptor 1	4.41	2.25.E-04
79746	ECHDC3	enoyl-CoA hydratase domain containing 3	-4.41	2.24.E-04
2533	FYB	FYN binding protein	4.40	2.24.E-04
27040	LAT	linker for activation of T-cells	4.40	2.26.E-04
6850	SYK	spleen associated tyrosine kinase	4.40	2.25.E-04
6100	RP9	retinitis pigmentosa 9 (autosomal dominant)	-4.40	2.24.E-04
3656	IRAK2	interleukin 1 receptor associated kinase 2	-4.39	2.27.E-04
55168	MRPS18A	mitochondrial ribosomal protein S18A	-4.39	2.26.E-04
55526	DHTKD1	dehydrogenase E1 and transketolase domain containing 1	-4.39	2.25.E-04
23213	SULF1	sulfatase 1	-4.39	2.25.E-04
256236	NAPSB	napsin B aspartic peptidase, pseudogene	4.38	2.30.E-04
64092	SAMSN1	SAM domain, SH3 domain and nuclear localization signals 1	4.38	2.29.E-04
3290	HSD11B1	hydroxysteroid 11-beta dehydrogenase 1	4.38	2.29.E-04
5360	PLTP	phospholipid transfer protein	4.38	2.31.E-04
3988	LIPA	lipase A, lysosomal acid type	4.37	2.36.E-04
59343	SENP2	SUMO1/sentrin/SMT3 specific peptidase 2	-4.37	2.35.E-04
1436	CSF1R	colony stimulating factor 1 receptor	4.36	2.43.E-04
4642	MYO1D	myosin ID	4.36	2.45.E-04
991	CDC20	cell division cycle 20	4.36	2.44.E-04
140885	SIRPA	signal regulatory protein alpha	4.36	2.44.E-04
23353	SUN1	Sad1 and UNC84 domain containing 1	-4.36	2.46.E-04
2194	FASN	fatty acid synthase	-4.36	2.45.E-04
25807	RHBDD3	rhomboid domain containing 3	4.36	2.50.E-04
84364	ARFGAP2	ADP ribosylation factor GTPase activating protein 2	-4.35	2.49.E-04
302	ANXA2	annexin A2	4.35	2.49.E-04
1471	CST3	cystatin C	4.35	2.48.E-04
902	CCNH	cyclin H	-4.35	2.47.E-04
811	CALR	calreticulin	4.35	2.52.E-04
54496	PRMT7	protein arginine methyltransferase 7	-4.34	2.60.E-04
8565	YARS	tyrosyl-tRNA synthetase	4.34	2.59.E-04
241	ALOX5AP	arachidonate 5-lipoxygenase activating protein	4.34	2.66.E-04
130733	TMEM178A	transmembrane protein 178A	4.33	2.66.E-04
79017	GGCT	gamma-glutamylcyclotransferase	-4.33	2.70.E-04
3557	IL1RN	interleukin 1 receptor antagonist	4.33	2.75.E-04
3654	IRAK1	interleukin 1 receptor associated kinase 1	4.33	2.77.E-04
56243	KIAA1217	KIAA1217	-4.33	2.76.E-04
6363	CCL19	C-C motif chemokine ligand 19	4.33	2.75.E-04
9114	ATP6V0D1	ATPase H+ transporting V0 subunit d1	4.32	2.77.E-04
201895	SMIM14	small integral membrane protein 14	4.32	2.77.E-04
7454	WAS	Wiskott-Aldrich syndrome	4.32	2.79.E-04
54331	GNG2	G protein subunit gamma 2	4.31	2.96.E-04
137872	ADHFE1	alcohol dehydrogenase, iron containing 1	-4.30	2.98.E-04
2717	GLA	galactosidase alpha	4.30	3.11.E-04
9677	PPIP5K1	diphosphoinositol pentakisphosphate kinase 1	4.30	3.10.E-04
5095	PCCA	propionyl-CoA carboxylase alpha subunit	-4.30	3.09.E-04
7462	LAT2	linker for activation of T-cells family member 2	4.29	3.11.E-04
51134	CEP83	centrosomal protein 83	4.29	3.13.E-04
57333	RCN3	reticulocalbin 3	4.29	3.12.E-04
157	GRK3	G protein-coupled receptor kinase 3	-4.29	3.11.E-04
55228	PNMAL1	paraneoplastic Ma antigen family like 1	4.28	3.10.E-04
84268	RPAIN	RPA interacting protein	-4.28	3.12.E-04
11309	SLCO2B1	solute carrier organic anion transporter family member 2B1	4.28	3.19.E-04
10263	CDK2AP2	cyclin dependent kinase 2 associated protein 2	4.28	3.23.E-04
11261	CHP1	calcineurin like EF-hand protein 1	-4.28	3.25.E-04
389136	VGLL3	vestigial like family member 3	4.27	3.24.E-04
55900	ZNF302	zinc finger protein 302	-4.27	3.26.E-04
9938	ARHGAP25	Rho GTPase activating protein 25	4.27	3.25.E-04
7107	GPR137B	G protein-coupled receptor 137B	4.27	3.29.E-04
2631	GBAS	glioblastoma amplified sequence	-4.27	3.28.E-04
125	ADH1B	alcohol dehydrogenase 1B (class I), beta polypeptide	-4.26	3.27.E-04
51110	LACTB2	lactamase beta 2	-4.26	3.27.E-04
10140	TOB1	transducer of ERBB2, 1	-4.26	3.26.E-04
132671	SPATA18	spermatogenesis associated 18	4.26	3.28.E-04

55589	BMP2K	BMP2 inducible kinase	4.26	3.27.E-04
8613	PLPP3	phospholipid phosphatase 3	-4.26	3.26.E-04
4953	ODC1	ornithine decarboxylase 1	4.26	3.33.E-04
245802	MS4A6E	membrane spanning 4-domains A6E	4.25	3.34.E-04
90853	SPOCD1	SPOC domain containing 1	4.25	3.33.E-04
124565	SLC38A10	solute carrier family 38 member 10	4.25	3.40.E-04
26227	PHGDH	phosphoglycerate dehydrogenase	-4.25	3.39.E-04
64231	MS4A6A	membrane spanning 4-domains A6A	4.24	3.41.E-04
6035	RNASE1	ribonuclease A family member 1, pancreatic	4.24	3.40.E-04
84987	COX14	COX14, cytochrome c oxidase assembly factor	-4.24	3.39.E-04
54504	CPVL	carboxypeptidase, vitellogenic like	4.24	3.41.E-04
205	AK4	adenylate kinase 4	-4.24	3.47.E-04
1031	CDKN2C	cyclin dependent kinase inhibitor 2C	-4.23	3.66.E-04
9332	CD163	CD163 molecule	4.23	3.65.E-04
96764	TGS1	trimethylguanosine synthase 1	-4.23	3.64.E-04
51263	MRPL30	mitochondrial ribosomal protein L30	-4.23	3.70.E-04
81037	CLPTM1L	CLPTM1 like	4.22	3.74.E-04
84134	TOMM40L	translocase of outer mitochondrial membrane 40 like	4.22	3.80.E-04
10400	PEMT	phosphatidylethanolamine N-methyltransferase	4.22	3.79.E-04
10261	IGSF6	immunoglobulin superfamily member 6	4.22	3.79.E-04
10318	TNIP1	TNFAIP3 interacting protein 1	4.22	3.85.E-04
55023	PHIP	pleckstrin homology domain interacting protein	-4.22	3.89.E-04
3074	HEXB	hexosaminidase subunit beta	4.21	4.00.E-04
3131	HLF	HLF, PAR bZIP transcription factor	-4.21	3.99.E-04
29944	PNMA3	paraneoplastic Ma antigen 3	-4.21	4.07.E-04
929	CD14	CD14 molecule	4.20	4.06.E-04
55803	ADAP2	ArfGAP with dual PH domains 2	4.20	4.05.E-04
664	BNIP3	BCL2 interacting protein 3	-4.20	4.04.E-04
378	ARF4	ADP ribosylation factor 4	4.20	4.08.E-04
4701	NDUFA7	NADH:ubiquinone oxidoreductase subunit A7	-4.20	4.09.E-04
712	C1QA	complement C1q A chain	4.20	4.11.E-04
55200	PLEKHG6	pleckstrin homology and RhoGEF domain containing G6	-4.20	4.10.E-04
80206	FHOD3	formin homology 2 domain containing 3	-4.19	4.11.E-04
2764	GMFB	glia maturation factor beta	4.19	4.10.E-04
3512	JCHAIN	joining chain of multimeric IgA and IgM	4.19	4.09.E-04
53833	IL20RB	interleukin 20 receptor subunit beta	4.19	4.13.E-04
219736	STOX1	storkhead box 1	-4.19	4.12.E-04
64983	MRPL32	mitochondrial ribosomal protein L32	-4.19	4.11.E-04
6195	RPS6KA1	ribosomal protein S6 kinase A1	4.19	4.10.E-04
2517	FUCA1	fucosidase, alpha-L- 1, tissue	4.19	4.09.E-04
5269	SERPINB6	serpin family B member 6	-4.19	4.08.E-04
3101	HK3	hexokinase 3	4.18	4.10.E-04
55374	TMCO6	transmembrane and coiled-coil domains 6	-4.18	4.09.E-04
6856	SYPL1	synaptophysin like 1	-4.18	4.17.E-04
822	CAPG	capping actin protein, gelsolin like	4.18	4.16.E-04
2936	GSR	glutathione-disulfide reductase	4.18	4.15.E-04
23404	EXOSC2	exosome component 2	-4.18	4.14.E-04
2532	ACKR1	atypical chemokine receptor 1 (Duffy blood group)	4.17	4.13.E-04
4233	MET	MET proto-oncogene, receptor tyrosine kinase	-4.17	4.14.E-04
50484	RRM2B	ribonucleotide reductase regulatory TP53 inducible subunit M2B	4.17	4.16.E-04
942	CD86	CD86 molecule	4.17	4.28.E-04
6050	RNH1	ribonuclease/angiogenin inhibitor 1	4.16	4.29.E-04
10243	GPHN	gephyrin	-4.16	4.30.E-04
80896	NPL	N-acetylneuraminate pyruvate lyase	4.16	4.30.E-04
7018	TF	transferrin	-4.16	4.31.E-04
4301	AFDN	afadin, adherens junction formation factor	-4.16	4.32.E-04
10072	DPP3	dipeptidyl peptidase 3	4.16	4.31.E-04
58477	SRPRB	SRP receptor beta subunit	4.16	4.30.E-04
3109	HLA-DMB	major histocompatibility complex, class II, DM beta	4.16	4.29.E-04
196527	ANO6	anoctamin 6	-4.15	4.39.E-04
50619	DEF6	DEF6, guanine nucleotide exchange factor	4.15	4.43.E-04
10020	GNE	glucosamine (UDP-N-acetyl)-2-epimerase/N-acetylmannosamine kinase	-4.14	4.46.E-04
719	C3AR1	complement C3a receptor 1	4.14	4.45.E-04
8877	SPHK1	sphingosine kinase 1	4.14	4.46.E-04
8459	TPST2	tyrosylprotein sulfotransferase 2	4.14	4.47.E-04
29127	RACGAP1	Rac GTPase activating protein 1	4.14	4.51.E-04
4999	ORC2	origin recognition complex subunit 2	-4.13	4.50.E-04

51588	PIAS4	protein inhibitor of activated STAT 4	-4.13	4.57.E-04
57185	NIPAL3	NIPA like domain containing 3	-4.13	4.56.E-04
56920	SEMA3G	semaphorin 3G	4.12	4.62.E-04
10525	HYOU1	hypoxia up-regulated 1	4.12	4.61.E-04
3625	INHBB	inhibin beta B subunit	4.12	4.60.E-04
9056	SLC7A7	solute carrier family 7 member 7	4.12	4.61.E-04
58191	CXCL16	C-X-C motif chemokine ligand 16	4.12	4.60.E-04
6832	SUPV3L1	Suv3 like RNA helicase	-4.12	4.59.E-04
1291	COL6A1	collagen type VI alpha 1 chain	4.12	4.60.E-04
3001	GZMA	granzyme A	4.11	4.63.E-04
138716	RPP25L	ribonuclease P/MRP subunit p25 like	-4.11	4.64.E-04
55022	PID1	phosphotyrosine interaction domain containing 1	4.11	4.68.E-04
2101	ESRRA	estrogen related receptor alpha	-4.11	4.67.E-04
84622	ZNF594	zinc finger protein 594	-4.11	4.72.E-04
143458	LDLRAD3	low density lipoprotein receptor class A domain containing 3	-4.10	4.83.E-04
214	ALCAM	activated leukocyte cell adhesion molecule	4.10	4.82.E-04
64098	PARVG	parvin gamma	4.10	4.90.E-04
7133	TNFRSF1B	TNF receptor superfamily member 1B	4.10	4.93.E-04
118980	SFXN2	sideroflexin 2	-4.09	4.98.E-04
1535	CYBA	cytochrome b-245 alpha chain	4.09	4.97.E-04
10840	ALDH1L1	aldehyde dehydrogenase 1 family member L1	-4.09	4.96.E-04
8727	CTNNAL1	catenin alpha like 1	-4.09	4.99.E-04
6355	CCL8	C-C motif chemokine ligand 8	4.08	5.06.E-04
158800	RHOXF1	Rhox homeobox family member 1	4.08	5.09.E-04
51057	WDPCP	WD repeat containing planar cell polarity effector	4.08	5.14.E-04
56952	PRTFDC1	phosphoribosyl transferase domain containing 1	4.08	5.15.E-04
58528	RRAGD	Ras related GTP binding D	-4.08	5.14.E-04
114876	OSBPL1A	oxysterol binding protein like 1A	-4.08	5.15.E-04
2822	GPLD1	glycosylphosphatidylinositol specific phospholipase D1	4.08	5.14.E-04
23179	RGL1	ral guanine nucleotide dissociation stimulator like 1	4.08	5.13.E-04
51296	SLC15A3	solute carrier family 15 member 3	4.07	5.16.E-04
9811	CTIF	cap binding complex dependent translation initiation factor	4.07	5.19.E-04
58986	TMEM8A	transmembrane protein 8A	4.07	5.20.E-04
5274	SERPINI1	serpin family I member 1	-4.07	5.23.E-04
55013	MCUB	mitochondrial calcium uniporter dominant negative beta subunit	4.07	5.22.E-04
54850	FBXL12	F-box and leucine rich repeat protein 12	-4.07	5.23.E-04
8857	FCGBP	Fc fragment of IgG binding protein	4.07	5.28.E-04
665	BNIP3L	BCL2 interacting protein 3 like	-4.07	5.29.E-04
1020	CDK5	cyclin dependent kinase 5	4.06	5.28.E-04
960	CD44	CD44 molecule (Indian blood group)	4.06	5.31.E-04
3162	HMOX1	heme oxygenase 1	4.06	5.33.E-04
79944	L2HGDH	L-2-hydroxyglutarate dehydrogenase	-4.06	5.38.E-04
4316	MMP7	matrix metallopeptidase 7	4.06	5.37.E-04
57502	NLGN4X	neuroligin 4, X-linked	-4.05	5.54.E-04
10961	ERP29	endoplasmic reticulum protein 29	4.05	5.53.E-04
2222	FDFT1	farnesyl-diphosphate farnesyltransferase 1	-4.05	5.58.E-04
55799	CACNA2D3	calcium voltage-gated channel auxiliary subunit alpha2delta 3	4.05	5.72.E-04
30817	ADGRE2	adhesion G protein-coupled receptor E2	4.04	5.77.E-04
5341	PLEK	pleckstrin	4.04	5.76.E-04
1632	ECI1	enoyl-CoA delta isomerase 1	-4.04	5.76.E-04
5781	PTPN11	protein tyrosine phosphatase, non-receptor type 11	4.04	5.89.E-04
10287	RGS19	regulator of G-protein signaling 19	4.04	5.95.E-04
283316	CD163L1	CD163 molecule like 1	4.04	5.94.E-04
143503	OR51E1	olfactory receptor family 51 subfamily E member 1	-4.03	6.01.E-04
4134	MAP4	microtubule associated protein 4	4.03	6.00.E-04
55335	NIPSNAP3B	nipsnap homolog 3B	-4.03	5.98.E-04
55876	GSDMB	gasdermin B	-4.03	6.05.E-04
9397	NMT2	N-myristoyltransferase 2	4.03	6.15.E-04
54675	CRLS1	cardiolipin synthase 1	-4.03	6.14.E-04
10234	LRRC17	leucine rich repeat containing 17	-4.02	6.22.E-04
57863	CADM3	cell adhesion molecule 3	4.02	6.23.E-04
10491	CRTAP	cartilage associated protein	4.02	6.29.E-04
54510	PCDH18	protocadherin 18	-4.01	6.49.E-04
7461	CLIP2	CAP-Gly domain containing linker protein 2	4.01	6.61.E-04
9728	SECISBP2L	SECIS binding protein 2 like	-4.01	6.60.E-04
4258	MGST2	microsomal glutathione S-transferase 2	-4.01	6.66.E-04
7337	UBE3A	ubiquitin protein ligase E3A	-4.01	6.67.E-04

79651	RHBDF2	rhomboid 5 homolog 2	4.01	6.69.E-04
1666	DECR1	2,4-dienoyl-CoA reductase 1, mitochondrial	-4.00	6.74.E-04
9377	COX5A	cytochrome c oxidase subunit 5A	-4.00	6.91.E-04
2734	GLG1	golgi glycoprotein 1	3.99	7.01.E-04
875	CBS	cystathionine-beta-synthase	-3.99	6.99.E-04
84966	IGSF21	immunoglobin superfamily member 21	3.99	7.09.E-04
53340	SPA17	sperm autoantigenic protein 17	3.99	7.08.E-04
2534	FYN	FYN proto-oncogene, Src family tyrosine kinase	3.99	7.07.E-04
121506	ERP27	endoplasmic reticulum protein 27	3.99	7.05.E-04
57522	SRGAP1	SLIT-ROBO Rho GTPase activating protein 1	3.99	7.10.E-04
64859	NABP1	nucleic acid binding protein 1	3.99	7.10.E-04
29887	SNX10	sorting nexin 10	3.99	7.09.E-04
3683	ITGAL	integrin subunit alpha L	3.98	7.17.E-04
4126	MANBA	mannosidase beta	3.98	7.23.E-04
1508	CTSB	cathepsin B	3.98	7.23.E-04
7386	UQCRFS1	ubiquinol-cytochrome c reductase, Rieske iron-sulfur polypeptide 1	-3.98	7.22.E-04
2207	FCER1G	Fc fragment of IgE receptor Ig	3.98	7.24.E-04
79699	ZYG11B	zyg-11 family member B, cell cycle regulator	-3.97	7.25.E-04
27286	SRPX2	sushi repeat containing protein, X-linked 2	3.97	7.23.E-04
10950	BTG3	BTG anti-proliferation factor 3	-3.97	7.26.E-04
22839	DLGAP4	DLG associated protein 4	3.97	7.30.E-04
8667	EIF3H	eukaryotic translation initiation factor 3 subunit H	-3.97	7.36.E-04
57103	TIGAR	TP53 induced glycolysis regulatory phosphatase	3.97	7.47.E-04
5777	PTPN6	protein tyrosine phosphatase, non-receptor type 6	3.96	7.53.E-04
6696	SPP1	secreted phosphoprotein 1	3.96	7.53.E-04
125113	KRT222	keratin 222	-3.96	7.59.E-04
8671	SLC4A4	solute carrier family 4 member 4	-3.96	7.58.E-04
283431	GAS2L3	growth arrest specific 2 like 3	3.95	7.56.E-04
374403	TBC1D10C	TBC1 domain family member 10C	3.95	7.62.E-04
84171	LOXL4	lysyl oxidase like 4	3.95	7.61.E-04
6788	STK3	serine/threonine kinase 3	-3.95	7.66.E-04
10227	MFSD10	major facilitator superfamily domain containing 10	3.95	7.65.E-04
9903	KLHL21	kelch like family member 21	-3.95	7.65.E-04
10460	TACC3	transforming acidic coiled-coil containing protein 3	3.95	7.78.E-04
430	ASCL2	achaete-scute family bHLH transcription factor 2	3.95	7.79.E-04
25849	PARM1	prostate androgen-regulated mucin-like protein 1	-3.95	7.77.E-04
9046	DOK2	docking protein 2	3.95	7.76.E-04
2768	GNA12	G protein subunit alpha 12	3.94	7.83.E-04
5699	PSMB10	proteasome subunit beta 10	3.94	7.82.E-04
5791	PTPRE	protein tyrosine phosphatase, receptor type E	3.94	7.80.E-04
2896	GRN	granulin	3.94	7.95.E-04
113	ADCY7	adenylate cyclase 7	3.94	8.00.E-04
115827	RAB3C	RAB3C, member RAS oncogene family	3.94	7.99.E-04
26061	HACL1	2-hydroxyacyl-CoA lyase 1	3.93	8.01.E-04
126	ADH1C	alcohol dehydrogenase 1C (class I), gamma polypeptide	-3.93	8.00.E-04
3357	HTR2B	5-hydroxytryptamine receptor 2B	3.92	8.24.E-04
80312	TET1	tet methylcytosine dioxygenase 1	-3.92	8.30.E-04
2099	ESR1	estrogen receptor 1	-3.92	8.28.E-04
115098	CCDC124	coiled-coil domain containing 124	3.92	8.34.E-04
55260	TMEM143	transmembrane protein 143	-3.92	8.36.E-04
23031	MAST3	microtubule associated serine/threonine kinase 3	3.92	8.34.E-04
8382	NME5	NME/NM23 family member 5	3.91	8.52.E-04
133383	SETD9	SET domain containing 9	-3.91	8.55.E-04
8673	VAMP8	vesicle associated membrane protein 8	3.91	8.61.E-04
57706	DENND1A	DENN domain containing 1A	3.91	8.66.E-04
115123	MARCH3	membrane associated ring-CH-type finger 3	-3.91	8.66.E-04
8050	PDHX	pyruvate dehydrogenase complex component X	-3.91	8.66.E-04
123920	CMTM3	CKLF like MARVEL transmembrane domain containing 3	3.91	8.65.E-04
80018	NAA25	N(alpha)-acetyltransferase 25, NatB auxiliary subunit	-3.90	8.75.E-04
31	ACACA	acetyl-CoA carboxylase alpha	-3.90	8.77.E-04
7305	TYROBP	TYRO protein tyrosine kinase binding protein	3.90	8.79.E-04
79752	ZFAND1	zinc finger AN1-type containing 1	-3.90	8.79.E-04
55357	TBC1D2	TBC1 domain family member 2	3.90	8.79.E-04
729359	PLIN4	perilipin 4	3.90	8.78.E-04
32	ACACB	acetyl-CoA carboxylase beta	-3.89	8.93.E-04
64174	DPEP2	dipeptidase 2	3.89	8.98.E-04
3071	NCKAP1L	NCK associated protein 1 like	3.89	9.02.E-04

79873	NUDT18	nudix hydrolase 18	3.89	9.15.E-04
220002	CYB561A3	cytochrome b561 family member A3	3.89	9.14.E-04
27173	SLC39A1	solute carrier family 39 member 1	3.89	9.24.E-04
23523	CABIN1	calcineurin binding protein 1	3.88	9.31.E-04
64747	MFSD1	major facilitator superfamily domain containing 1	3.88	9.34.E-04
9697	TRAM2	translocation associated membrane protein 2	3.88	9.34.E-04
79085	SLC25A23	solute carrier family 25 member 23	-3.88	9.33.E-04
1104	RCC1	regulator of chromosome condensation 1	3.88	9.38.E-04
4891	SLC11A2	solute carrier family 11 member 2	3.88	9.41.E-04
5329	PLAUR	plasminogen activator, urokinase receptor	3.88	9.44.E-04
4644	MYO5A	myosin VA	3.88	9.43.E-04
80704	SLC19A3	solute carrier family 19 member 3	-3.87	9.59.E-04
60	ACTB	actin beta	3.87	9.61.E-04
2956	MSH6	mutS homolog 6	-3.87	9.74.E-04
5654	HTRA1	HtrA serine peptidase 1	3.87	9.81.E-04
8476	CDC42BPA	CDC42 binding protein kinase alpha	-3.87	9.84.E-04
23166	STAB1	stabilin 1	3.86	9.94.E-04
51099	ABHD5	abhydrolase domain containing 5	-3.86	9.98.E-04
409	ARRB2	arrestin beta 2	3.86	1.01.E-03
51199	NIN	ninein	3.86	1.02.E-03
2123	EVI2A	ecotropic viral integration site 2A	3.85	1.03.E-03
3059	HCLS1	hematopoietic cell-specific Lyn substrate 1	3.85	1.03.E-03
287	ANK2	ankyrin 2	-3.85	1.04.E-03
54927	CHCHD3	coiled-coil-helix-coiled-coil-helix domain containing 3	-3.85	1.04.E-03
6789	STK4	serine/threonine kinase 4	3.85	1.04.E-03
6730	SRP68	signal recognition particle 68	-3.85	1.04.E-03
221303	FAM162B	family with sequence similarity 162 member B	-3.85	1.04.E-03
10981	RAB32	RAB32, member RAS oncogene family	3.85	1.05.E-03
55852	TEX2	testis expressed 2	3.84	1.06.E-03
29071	C1GALT1C1	C1GALT1 specific chaperone 1	3.84	1.06.E-03
84803	GPAT3	glycerol-3-phosphate acyltransferase 3	-3.84	1.06.E-03
5465	PPARA	peroxisome proliferator activated receptor alpha	-3.84	1.07.E-03
128346	C1orf162	chromosome 1 open reading frame 162	3.84	1.07.E-03
9276	COPB2	coatomer protein complex subunit beta 2	3.84	1.08.E-03
3216	HOXB6	homeobox B6	-3.84	1.08.E-03
57533	TBC1D14	TBC1 domain family member 14	3.83	1.09.E-03
987	LRBA	LPS responsive beige-like anchor protein	3.83	1.09.E-03
199	AIF1	allograft inflammatory factor 1	3.83	1.10.E-03
23406	COTL1	coactosin like F-actin binding protein 1	3.83	1.11.E-03
57380	MRS2	MRS2, magnesium transporter	-3.83	1.11.E-03
23597	ACOT9	acyl-CoA thioesterase 9	3.83	1.11.E-03
55840	EAF2	ELL associated factor 2	3.82	1.12.E-03
51320	MEX3C	mex-3 RNA binding family member C	-3.82	1.12.E-03
116159	CYYR1	cysteine and tyrosine rich 1	-3.82	1.15.E-03
53834	FGFRL1	fibroblast growth factor receptor-like 1	-3.81	1.17.E-03
124222	PAQR4	progestin and adipoQ receptor family member 4	3.81	1.17.E-03
231	AKR1B1	aldo-keto reductase family 1 member B	3.81	1.19.E-03
57546	PDP2	pyruvate dehyrogenase phosphatase catalytic subunit 2	-3.81	1.20.E-03
23235	SIK2	salt inducible kinase 2	-3.81	1.20.E-03
64965	MRPS9	mitochondrial ribosomal protein S9	-3.81	1.20.E-03
4267	CD99	CD99 molecule	3.81	1.21.E-03
5833	PCYT2	phosphate cytidylyltransferase 2, ethanolamine	-3.80	1.21.E-03
1203	CLN5	ceroid-lipofuscinosis, neuronal 5	3.80	1.21.E-03
56886	UGGT1	UDP-glucose glycoprotein glucosyltransferase 1	3.79	1.29.E-03
83483	PLVAP	plasmalemma vesicle associated protein	3.79	1.30.E-03
1491	CTH	cystathionine gamma-lyase	-3.79	1.31.E-03
81502	HM13	histocompatibility minor 13	3.79	1.31.E-03
2162	F13A1	coagulation factor XIII A chain	3.79	1.31.E-03
3111	HLA-DOA	major histocompatibility complex, class II, DO alpha	3.79	1.31.E-03
10653	SPINT2	serine peptidase inhibitor, Kunitz type 2	3.78	1.32.E-03
79171	RBM42	RNA binding motif protein 42	3.78	1.32.E-03
3965	LGALS9	galectin 9	3.78	1.32.E-03
717	C2	complement C2	3.78	1.33.E-03
25996	REXO2	RNA exonuclease 2	3.78	1.33.E-03
1478	CSTF2	cleavage stimulation factor subunit 2	3.78	1.32.E-03
3912	LAMB1	laminin subunit beta 1	-3.78	1.33.E-03
81888	HYI	hydroxypyruvate isomerase (putative)	-3.78	1.33.E-03

401052	LOC401052	uncharacterized LOC401052	-3.78	1.33.E-03
5641	LGMN	legumain	3.78	1.34.E-03
89846	FGD3	FYVE, RhoGEF and PH domain containing 3	3.77	1.35.E-03
4067	LYN	LYN proto-oncogene, Src family tyrosine kinase	3.77	1.35.E-03
1290	COL5A2	collagen type V alpha 2 chain	3.77	1.36.E-03
54963	UCKL1	uridine-cytidine kinase 1 like 1	-3.77	1.39.E-03
6210	RPS15A	ribosomal protein S15a	-3.77	1.40.E-03
10370	CITED2	Cbp/p300 interacting transactivator with Glu/Asp rich carboxy-terminal domain 2	-3.76	1.42.E-03
54941	RNF125	ring finger protein 125	-3.76	1.42.E-03
6282	S100A11	S100 calcium binding protein A11	3.76	1.42.E-03
54749	EPDR1	ependymin related 1	3.76	1.43.E-03
8460	TPST1	tyrosylprotein sulfotransferase 1	-3.76	1.43.E-03
643837	LINC01128	long intergenic non-protein coding RNA 1128	-3.76	1.43.E-03
8925	HERC1	HECT and RLD domain containing E3 ubiquitin protein ligase family member 1	-3.76	1.43.E-03
80820	EEPD1	endonuclease/exonuclease/phosphatase family domain containing 1	-3.76	1.43.E-03
10226	PLIN3	perilipin 3	3.75	1.47.E-03
23411	SIRT1	sirtuin 1	-3.75	1.47.E-03
10046	MAMLD1	mastermind like domain containing 1	-3.75	1.47.E-03
54913	RPP25	ribonuclease P/MRP subunit p25	3.75	1.47.E-03
6424	SFRP4	secreted frizzled related protein 4	3.75	1.47.E-03
8718	TNFRSF25	TNF receptor superfamily member 25	3.75	1.47.E-03
3954	LETM1	leucine zipper and EF-hand containing transmembrane protein 1	-3.75	1.49.E-03
65009	NDRG4	NDRG family member 4	-3.74	1.49.E-03
81622	UNC93B1	unc-93 homolog B1 (C. elegans)	3.74	1.49.E-03
23517	SKIV2L2	Ski2 like RNA helicase 2	-3.74	1.49.E-03
55288	RHOT1	ras homolog family member T1	-3.74	1.49.E-03
8445	DYRK2	dual specificity tyrosine phosphorylation regulated kinase 2	3.74	1.49.E-03
5054	SERPINE1	serpin family E member 1	3.74	1.49.E-03
6913	TBX15	T-box 15	-3.74	1.49.E-03
57669	EPB41L5	erythrocyte membrane protein band 4.1 like 5	-3.74	1.51.E-03
6643	SNX2	sorting nexin 2	3.73	1.51.E-03
10148	EBI3	Epstein-Barr virus induced 3	3.73	1.51.E-03
10398	MYL9	myosin light chain 9	3.73	1.51.E-03
2131	EXT1	exostosin glycosyltransferase 1	3.73	1.51.E-03
7533	YWHAH	tyrosine 3-monooxygenase/tryptophan 5-monooxygenase activation protein eta	3.73	1.52.E-03
55186	SLC25A36	solute carrier family 25 member 36	-3.73	1.52.E-03
27106	ARRDC2	arrestin domain containing 2	3.73	1.52.E-03
10654	PMVK	phosphomevalonate kinase	3.73	1.52.E-03
24145	PANX1	pannexin 1	3.73	1.52.E-03
1612	DAPK1	death associated protein kinase 1	3.73	1.52.E-03
130399	ACVR1C	activin A receptor type 1C	-3.73	1.52.E-03
79947	DHDDS	dehydrodolichyl diphosphate synthase subunit	3.73	1.53.E-03
114883	OSBPL9	oxysterol binding protein like 9	3.72	1.54.E-03
51816	CECR1	cat eye syndrome chromosome region, candidate 1	3.72	1.56.E-03
607	BCL9	B-cell CLL/lymphoma 9	-3.72	1.57.E-03
10240	MRPS31	mitochondrial ribosomal protein S31	-3.72	1.58.E-03
374291	NDUFS7	NADH:ubiquinone oxidoreductase core subunit S7	-3.72	1.59.E-03
80851	SH3BP5L	SH3 binding domain protein 5 like	3.72	1.60.E-03
7881	KCNAB1	potassium voltage-gated channel subfamily A member regulatory beta subunit 1	-3.71	1.61.E-03
6352	CCL5	C-C motif chemokine ligand 5	3.71	1.61.E-03
2178	FANCE	Fanconi anemia complementation group E	-3.71	1.61.E-03
23203	PMPCA	peptidase, mitochondrial processing alpha subunit	-3.71	1.61.E-03
10857	PGRMC1	progesterone receptor membrane component 1	-3.71	1.61.E-03
9334	B4GALT5	beta-1,4-galactosyltransferase 5	3.71	1.61.E-03
2246	FGF1	fibroblast growth factor 1	3.71	1.62.E-03
5230	PGK1	phosphoglycerate kinase 1	3.71	1.62.E-03
2592	GALT	galactose-1-phosphate uridylyltransferase	-3.71	1.62.E-03
51560	RAB6B	RAB6B, member RAS oncogene family	3.71	1.63.E-03
6039	RNASE6	ribonuclease A family member k6	3.70	1.64.E-03
10289	EIF1B	eukaryotic translation initiation factor 1B	-3.70	1.66.E-03
5034	P4HB	prolyl 4-hydroxylase subunit beta	3.70	1.66.E-03
757	TMEM50B	transmembrane protein 50B	-3.70	1.67.E-03
9308	CD83	CD83 molecule	3.70	1.68.E-03
144132	DNHD1	dynein heavy chain domain 1	-3.70	1.68.E-03
2639	GCDH	glutaryl-CoA dehydrogenase	-3.70	1.68.E-03
79624	ARMT1	acidic residue methyltransferase 1	-3.70	1.68.E-03
10808	HSPH1	heat shock protein family H (Hsp110) member 1	3.69	1.69.E-03

79180	EFHD2	EF-hand domain family member D2	3.69	1.69.E-03
51150	SDF4	stromal cell derived factor 4	3.69	1.71.E-03
10788	IQGAP2	IQ motif containing GTPase activating protein 2	3.69	1.73.E-03
441549	CDNF	cerebral dopamine neurotrophic factor	-3.69	1.74.E-03
63940	GPSM3	G-protein signaling modulator 3	3.69	1.73.E-03
84318	CCDC77	coiled-coil domain containing 77	3.68	1.75.E-03
6335	SCN9A	sodium voltage-gated channel alpha subunit 9	3.68	1.75.E-03
387	RHOA	ras homolog family member A	3.68	1.77.E-03
6390	SDHB	succinate dehydrogenase complex iron sulfur subunit B	-3.68	1.77.E-03
330	BIRC3	baculoviral IAP repeat containing 3	3.68	1.78.E-03
6902	TBCA	tubulin folding cofactor A	-3.68	1.78.E-03
157922	CAMSAP1	calmodulin regulated spectrin associated protein 1	-3.68	1.78.E-03
55711	FAR2	fatty acyl-CoA reductase 2	3.68	1.78.E-03
10870	HCST	hematopoietic cell signal transducer	3.67	1.79.E-03
6208	RPS14	ribosomal protein S14	-3.67	1.79.E-03
1027	CDKN1B	cyclin dependent kinase inhibitor 1B	-3.67	1.79.E-03
5445	PON2	paraoxonase 2	3.67	1.79.E-03
944	TNFSF8	tumor necrosis factor superfamily member 8	3.67	1.80.E-03
1490	CTGF	connective tissue growth factor	3.66	1.83.E-03
55714	TENM3	teneurin transmembrane protein 3	-3.66	1.84.E-03
64969	MRPS5	mitochondrial ribosomal protein S5	-3.66	1.85.E-03
56950	SMYD2	SET and MYND domain containing 2	3.66	1.87.E-03
11025	LILRB3	leukocyte immunoglobulin like receptor B3	3.66	1.87.E-03
63892	THADA	THADA, armadillo repeat containing	3.66	1.87.E-03
10778	ZNF271P	zinc finger protein 271, pseudogene	3.65	1.88.E-03
51284	TLR7	toll like receptor 7	3.65	1.88.E-03
23462	HEY1	hes related family bHLH transcription factor with YRPW motif 1	-3.65	1.87.E-03
2690	GHR	growth hormone receptor	-3.65	1.87.E-03
25907	TMEM158	transmembrane protein 158 (gene/pseudogene)	3.65	1.87.E-03
55577	NAGK	N-acetylglucosamine kinase	3.65	1.89.E-03
116255	MOGAT1	monoacylglycerol O-acyltransferase 1	-3.64	1.92.E-03
64744	SMAP2	small ArfGAP2	3.64	1.94.E-03
93210	PGAP3	post-GPI attachment to proteins 3	3.64	1.95.E-03
55704	CCDC88A	coiled-coil domain containing 88A	3.64	1.96.E-03
5880	RAC2	ras-related C3 botulinum toxin substrate 2 (rho family, small GTP binding protein Rac2)	3.64	1.96.E-03
50808	AK3	adenylate kinase 3	-3.64	1.96.E-03
55605	KIF21A	kinesin family member 21A	-3.64	1.98.E-03
23743	BHMT2	betaine--homocysteine S-methyltransferase 2	3.63	1.99.E-03
10492	SYNCRIP	synaptotagmin binding cytoplasmic RNA interacting protein	3.63	1.99.E-03
6586	SLIT3	slit guidance ligand 3	3.63	1.99.E-03
689	BTF3	basic transcription factor 3	-3.63	2.01.E-03
115201	ATG4A	autophagy related 4A cysteine peptidase	3.63	2.01.E-03
2999	GZMH	granzyme H	3.63	2.01.E-03
23028	KDM1A	lysine demethylase 1A	-3.63	2.02.E-03
221504	ZBTB9	zinc finger and BTB domain containing 9	-3.63	2.02.E-03
64776	C11orf1	chromosome 11 open reading frame 1	-3.62	2.02.E-03
54883	CWC25	CWC25 spliceosome associated protein homolog	-3.62	2.02.E-03
9404	LPXN	leupaxin	3.62	2.04.E-03
2519	FUCA2	fucosidase, alpha-L- 2, plasma	3.62	2.07.E-03
695	BTK	Bruton tyrosine kinase	3.62	2.08.E-03
7965	AIMP2	aminoacyl tRNA synthetase complex interacting multifunctional protein 2	3.62	2.09.E-03
54436	SH3TC1	SH3 domain and tetratricopeptide repeats 1	3.62	2.09.E-03
26589	MRPL46	mitochondrial ribosomal protein L46	-3.62	2.09.E-03
6419	SETMAR	SET domain and mariner transposase fusion gene	-3.61	2.10.E-03
54440	SASH3	SAM and SH3 domain containing 3	3.61	2.13.E-03
1200	TPP1	tripeptidyl peptidase 1	3.61	2.15.E-03
5265	SERPINA1	serpin family A member 1	3.61	2.15.E-03
7280	TUBB2A	tubulin beta 2A class IIa	3.61	2.15.E-03
10938	EHD1	EH domain containing 1	-3.61	2.16.E-03
5549	PRELP	proline and arginine rich end leucine rich repeat protein	3.60	2.16.E-03
11331	PHB2	prohibitin 2	-3.60	2.17.E-03
54502	RBM47	RNA binding motif protein 47	3.60	2.17.E-03
83442	SH3BGRL3	SH3 domain binding glutamate rich protein like 3	3.60	2.18.E-03
160897	GPR180	G protein-coupled receptor 180	-3.60	2.18.E-03
64210	MMS19	MMS19 homolog, cytosolic iron-sulfur assembly component	-3.60	2.18.E-03
9488	PIGB	phosphatidylinositol glycan anchor biosynthesis class B	3.60	2.20.E-03
1786	DNMT1	DNA methyltransferase 1	3.59	2.20.E-03

64374	SIL1	SIL1 nucleotide exchange factor	3.59	2.22.E-03
1534	CYB561	cytochrome b561	3.59	2.22.E-03
5372	PMM1	phosphomannomutase 1	-3.59	2.21.E-03
377	ARF3	ADP ribosylation factor 3	3.59	2.23.E-03
84140	FAM161A	family with sequence similarity 161 member A	-3.59	2.24.E-03
826	CAPNS1	calpain small subunit 1	3.59	2.26.E-03
8864	PER2	period circadian clock 2	-3.59	2.26.E-03
133584	EGFLAM	EGF like, fibronectin type III and laminin G domains	-3.59	2.26.E-03
6261	RYR1	ryanodine receptor 1	3.59	2.26.E-03
3820	KLRB1	killer cell lectin like receptor B1	3.58	2.28.E-03
10618	TGOLN2	trans-golgi network protein 2	3.58	2.28.E-03
3689	ITGB2	integrin subunit beta 2	3.58	2.28.E-03
23336	SYNM	synemin	3.58	2.30.E-03
349565	NMNAT3	nicotinamide nucleotide adenylyltransferase 3	-3.58	2.31.E-03
7405	UVRAG	UV radiation resistance associated	-3.58	2.31.E-03
8439	NSMAF	neutral sphingomyelinase activation associated factor	3.58	2.31.E-03
131118	DNAJC19	DnaJ heat shock protein family (Hsp40) member C19	-3.58	2.30.E-03
191	AHCY	adenosylhomocysteinase	3.58	2.31.E-03
1841	DTYMK	deoxythymidylate kinase	3.58	2.31.E-03
8320	EOMES	eomesodermin	3.58	2.31.E-03
11314	CD300A	CD300a molecule	3.58	2.32.E-03
10752	CHL1	cell adhesion molecule L1 like	-3.58	2.31.E-03
124935	SLC43A2	solute carrier family 43 member 2	3.57	2.31.E-03
8685	MARCO	macrophage receptor with collagenous structure	3.57	2.32.E-03
586	BCAT1	branched chain amino acid transaminase 1	3.57	2.32.E-03
10209	EIF1	eukaryotic translation initiation factor 1	-3.57	2.33.E-03
7779	SLC30A1	solute carrier family 30 member 1	3.57	2.32.E-03
9980	DOPEY2	dopey family member 2	3.57	2.33.E-03
55140	ELP3	elongator acetyltransferase complex subunit 3	3.57	2.33.E-03
6916	TBXAS1	thromboxane A synthase 1	3.57	2.37.E-03
4689	NCF4	neutrophil cytosolic factor 4	3.57	2.37.E-03
55070	DET1	de-etiolated homolog 1 (Arabidopsis)	-3.57	2.38.E-03
10130	PDIA6	protein disulfide isomerase family A member 6	3.57	2.39.E-03
6667	SP1	Sp1 transcription factor	-3.56	2.39.E-03
220388	CCDC89	coiled-coil domain containing 89	3.56	2.40.E-03
84886	C1orf198	chromosome 1 open reading frame 198	3.56	2.45.E-03
80318	GKAP1	G kinase anchoring protein 1	-3.56	2.49.E-03
8411	EEA1	early endosome antigen 1	3.55	2.51.E-03
4837	NNMT	nicotinamide N-methyltransferase	3.55	2.52.E-03
1043	CD52	CD52 molecule	3.55	2.52.E-03
23414	ZFPM2	zinc finger protein, FOG family member 2	-3.55	2.54.E-03
27102	EIF2AK1	eukaryotic translation initiation factor 2 alpha kinase 1	3.55	2.54.E-03
57415	C3orf14	chromosome 3 open reading frame 14	3.55	2.54.E-03
261729	STEAP2	STEAP2 metalloreductase	3.55	2.57.E-03
80023	NRSN2	neurensin 2	-3.54	2.59.E-03
10266	RAMP2	receptor activity modifying protein 2	-3.54	2.58.E-03
84935	MEDAG	mesenteric estrogen dependent adipogenesis	3.54	2.59.E-03
196463	PLBD2	phospholipase B domain containing 2	3.54	2.59.E-03
84695	LOXL3	lysyl oxidase like 3	3.54	2.60.E-03
1119	CHKA	choline kinase alpha	-3.54	2.61.E-03
11316	COPE	coatomer protein complex subunit epsilon	3.54	2.60.E-03
5621	PRNP	prion protein	3.54	2.61.E-03
103910	MYL12B	myosin light chain 12B	3.54	2.62.E-03
7852	CXCR4	C-X-C motif chemokine receptor 4	3.54	2.62.E-03
8677	STX10	syntaxin 10	3.54	2.63.E-03
2195	FAT1	FAT atypical cadherin 1	3.54	2.64.E-03
10320	IKZF1	IKAROS family zinc finger 1	3.54	2.64.E-03
23224	SYNE2	spectrin repeat containing nuclear envelope protein 2	-3.54	2.65.E-03
963	CD53	CD53 molecule	3.53	2.66.E-03
80310	PDGFD	platelet derived growth factor D	3.53	2.66.E-03
54491	FAM105A	family with sequence similarity 105 member A	3.53	2.67.E-03
159371	SLC35G1	solute carrier family 35 member G1	3.53	2.67.E-03
8863	PER3	period circadian clock 3	-3.53	2.68.E-03
51144	HSD17B12	hydroxysteroid 17-beta dehydrogenase 12	-3.53	2.68.E-03
91860	CALML4	calmodulin like 4	3.53	2.70.E-03
5007	OSBP	oxysterol binding protein	-3.53	2.70.E-03
8028	MLLT10	myeloid/lymphoid or mixed-lineage leukemia; translocated to, 10	-3.53	2.70.E-03

971	CD72	CD72 molecule	3.52	2.71.E-03
222256	CDHR3	cadherin related family member 3	-3.52	2.73.E-03
2124	EVI2B	ecotropic viral integration site 2B	3.52	2.75.E-03
57171	DOLPP1	dolichyldiphosphatase 1	-3.52	2.75.E-03
8703	B4GALT3	beta-1,4-galactosyltransferase 3	-3.52	2.76.E-03
252969	NEIL2	nei like DNA glycosylase 2	3.52	2.77.E-03
7030	TFE3	transcription factor binding to IGHM enhancer 3	3.52	2.77.E-03
4239	MFAP4	microfibrillar associated protein 4	3.52	2.78.E-03
366	AQP9	aquaporin 9	3.52	2.79.E-03
90102	PHLDB2	pleckstrin homology like domain family B member 2	-3.52	2.79.E-03
115572	FAM46B	family with sequence similarity 46 member B	-3.52	2.79.E-03
92400	RBM18	RNA binding motif protein 18	-3.51	2.81.E-03
2617	GARS	glycyl-tRNA synthetase	3.51	2.81.E-03
93343	MVB12A	multivesicular body subunit 12A	3.51	2.83.E-03
4256	MGP	matrix Gla protein	3.51	2.84.E-03
64175	P3H1	prolyl 3-hydroxylase 1	3.51	2.85.E-03
51087	YBX2	Y-box binding protein 2	-3.51	2.86.E-03
83608	C18orf21	chromosome 18 open reading frame 21	-3.50	2.86.E-03
7805	LAPTM5	lysosomal protein transmembrane 5	3.50	2.87.E-03
23609	MKRN2	makorin ring finger protein 2	-3.50	2.89.E-03
55356	SLC22A15	solute carrier family 22 member 15	3.50	2.88.E-03
8717	TRADD	TNFRSF1A associated via death domain	3.50	2.88.E-03
3099	HK2	hexokinase 2	-3.50	2.88.E-03
27154	BRPF3	bromodomain and PHD finger containing 3	-3.50	2.88.E-03
8535	CBX4	chromobox 4	3.50	2.91.E-03
540	ATP7B	ATPase copper transporting beta	-3.50	2.93.E-03
252995	FNDC5	fibronectin type Ⅲ domain containing 5	-3.49	2.94.E-03
23705	CADM1	cell adhesion molecule 1	-3.49	2.95.E-03
593	BCKDHA	branched chain keto acid dehydrogenase E1, alpha polypeptide	-3.49	2.94.E-03
2167	FABP4	fatty acid binding protein 4	-3.49	2.96.E-03
10871	CD300C	CD300c molecule	3.49	2.96.E-03
11167	FSTL1	follistatin like 1	3.49	2.96.E-03
10495	ENOX2	ecto-NOX disulfide-thiol exchanger 2	3.49	2.99.E-03
285590	SH3PXD2B	SH3 and PX domains 2B	3.49	3.02.E-03
6277	S100A6	S100 calcium binding protein A6	3.49	3.02.E-03
80183	RUBCNL	RUN and cysteine rich domain containing beclin 1 interacting protein like	3.49	3.02.E-03
84858	ZNF503	zinc finger protein 503	3.48	3.04.E-03
8839	WISP2	WNT1 inducible signaling pathway protein 2	3.48	3.05.E-03
3212	HOXB2	homeobox B2	-3.48	3.06.E-03
9249	DHRS3	dehydrogenase/reductase 3	-3.48	3.09.E-03
64333	ARHGAP9	Rho GTPase activating protein 9	3.48	3.11.E-03
4650	MYO9B	myosin IXB	3.47	3.13.E-03
9588	PRDX6	peroxiredoxin 6	-3.47	3.14.E-03
3115	HLA-DPB1	major histocompatibility complex, class Ⅱ, DP beta 1	3.47	3.16.E-03
1780	DYNC1I1	dynein cytoplasmic 1 intermediate chain 1	3.47	3.16.E-03
83607	AMMECR1L	AMMECR1 like	-3.47	3.18.E-03
23165	NUP205	nucleoporin 205	3.47	3.19.E-03
2908	NR3C1	nuclear receptor subfamily 3 group C member 1	-3.47	3.19.E-03
4363	ABCC1	ATP binding cassette subfamily C member 1	3.47	3.19.E-03
51291	GMIP	GEM interacting protein	3.47	3.19.E-03
8309	ACOX2	acyl-CoA oxidase 2	-3.47	3.19.E-03
4706	NDUFAB1	NADH:ubiquinone oxidoreductase subunit AB1	-3.47	3.19.E-03
118429	ANTXR2	anthrax toxin receptor 2	-3.47	3.18.E-03
728	C5AR1	complement C5a receptor 1	3.46	3.23.E-03
7417	VDAC2	voltage dependent anion channel 2	-3.46	3.23.E-03
1794	DOCK2	dedicator of cytokinesis 2	3.46	3.23.E-03
8165	AKAP1	A-kinase anchoring protein 1	-3.46	3.24.E-03
9262	STK17B	serine/threonine kinase 17b	3.46	3.25.E-03
6545	SLC7A4	solute carrier family 7 member 4	3.46	3.25.E-03
2875	GPT	glutamic--pyruvic transaminase	-3.46	3.26.E-03
349152	DPY19L2P2	DPY19L2 pseudogene 2	-3.46	3.25.E-03
64789	EXO5	exonuclease 5	3.46	3.26.E-03
9124	PDLIM1	PDZ and LIM domain 1	3.46	3.26.E-03
79411	GLB1L	galactosidase beta 1 like	3.46	3.27.E-03
54823	SWT1	SWT1, RNA endoribonuclease homolog	-3.46	3.29.E-03
153769	SH3RF2	SH3 domain containing ring finger 2	3.46	3.30.E-03
10908	PNPLA6	patatin like phospholipase domain containing 6	3.45	3.31.E-03

8702	B4GALT4	beta-1,4-galactosyltransferase 4	3.45	3.30.E-03
122525	C14orf28	chromosome 14 open reading frame 28	-3.45	3.32.E-03
1271	CNTFR	ciliary neurotrophic factor receptor	-3.45	3.35.E-03
10245	TIMM17B	translocase of inner mitochondrial membrane 17 homolog B (yeast)	-3.45	3.35.E-03
2217	FCGRT	Fc fragment of IgG receptor and transporter	3.45	3.36.E-03
54764	ZRANB1	zinc finger RANBP2-type containing 1	-3.45	3.36.E-03
11259	FILIP1L	filamin A interacting protein 1 like	3.45	3.37.E-03
219654	ZCCHC24	zinc finger CCHC-type containing 24	3.45	3.38.E-03
64641	EBF2	early B-cell factor 2	-3.45	3.38.E-03
11010	GLIPR1	GLI pathogenesis related 1	3.44	3.39.E-03
2495	FTH1	ferritin heavy chain 1	3.44	3.42.E-03
1760	DMPK	dystrophia myotonica protein kinase	3.44	3.42.E-03
8660	IRS2	insulin receptor substrate 2	-3.44	3.46.E-03
3113	HLA-DPA1	major histocompatibility complex, class II, DP alpha 1	3.44	3.46.E-03
9935	MAFB	MAF bZIP transcription factor B	3.44	3.47.E-03
4647	MYO7A	myosin VIIA	3.44	3.47.E-03
9552	SPAG7	sperm associated antigen 7	-3.44	3.49.E-03
8034	SLC25A16	solute carrier family 25 member 16	-3.44	3.49.E-03
51760	SYT17	synaptotagmin 17	-3.43	3.50.E-03
2960	GTF2E1	general transcription factor IIE subunit 1	3.43	3.50.E-03
9967	THRAP3	thyroid hormone receptor associated protein 3	-3.43	3.52.E-03
51020	HDDC2	HD domain containing 2	-3.43	3.54.E-03
1992	SERPINB1	serpin family B member 1	3.43	3.55.E-03
9604	RNF14	ring finger protein 14	3.43	3.57.E-03
27247	NFU1	NFU1 iron-sulfur cluster scaffold	-3.42	3.61.E-03
90423	ATP6V1E2	ATPase H+ transporting V1 subunit E2	-3.42	3.62.E-03
29926	GMPPA	GDP-mannose pyrophosphorylase A	3.42	3.63.E-03
116441	TM4SF18	transmembrane 4 L six family member 18	-3.42	3.63.E-03
4753	NELL2	neural EGFL like 2	3.42	3.63.E-03
23136	EPB41L3	erythrocyte membrane protein band 4.1 like 3	3.42	3.65.E-03
51338	MS4A4A	membrane spanning 4-domains A4A	3.42	3.65.E-03
1230	CCR1	C-C motif chemokine receptor 1	3.42	3.67.E-03
55750	AGK	acylglycerol kinase	-3.42	3.67.E-03
915	CD3D	CD3d molecule	3.42	3.68.E-03
10060	ABCC9	ATP binding cassette subfamily C member 9	-3.41	3.71.E-03
220	ALDH1A3	aldehyde dehydrogenase 1 family member A3	3.41	3.70.E-03
1072	CFL1	cofilin 1	3.41	3.70.E-03
84888	SPPL2A	signal peptide peptidase like 2A	3.41	3.70.E-03
7289	TULP3	tubby like protein 3	-3.41	3.74.E-03
11112	HIBADH	3-hydroxyisobutyrate dehydrogenase	-3.41	3.75.E-03
9770	RASSF2	Ras association domain family member 2	3.41	3.75.E-03
64764	CREB3L2	cAMP responsive element binding protein 3 like 2	3.41	3.76.E-03
8424	BBOX1	gamma-butyrobetaine hydroxylase 1	3.41	3.76.E-03
285195	SLC9A9	solute carrier family 9 member A9	3.41	3.76.E-03
5784	PTPN14	protein tyrosine phosphatase, non-receptor type 14	-3.41	3.75.E-03
1629	DBT	dihydrolipoamide branched chain transacylase E2	-3.41	3.76.E-03
4818	NKG7	natural killer cell granule protein 7	3.41	3.75.E-03
11214	AKAP13	A-kinase anchoring protein 13	3.41	3.75.E-03
84791	LINC00467	long intergenic non-protein coding RNA 467	-3.41	3.75.E-03
22795	NID2	nidogen 2	-3.40	3.76.E-03
63874	ABHD4	abhydrolase domain containing 4	3.40	3.76.E-03
10461	MERTK	MER proto-oncogene, tyrosine kinase	3.40	3.76.E-03
83892	KCTD10	potassium channel tetramerization domain containing 10	3.40	3.78.E-03
1948	EFNB2	ephrin B2	-3.40	3.80.E-03
222389	BEND7	BEN domain containing 7	-3.40	3.83.E-03
79415	C17orf62	chromosome 17 open reading frame 62	3.40	3.83.E-03
5579	PRKCB	protein kinase C beta	3.40	3.83.E-03
57701	NCKAP5L	NCK associated protein 5 like	3.40	3.83.E-03
29062	WDR91	WD repeat domain 91	3.40	3.84.E-03
23335	WDR7	WD repeat domain 7	3.40	3.86.E-03
79581	SLC52A2	solute carrier family 52 member 2	3.40	3.87.E-03
27010	TPK1	thiamin pyrophosphokinase 1	3.39	3.92.E-03
253959	RALGAPA1	Ral GTPase activating protein catalytic alpha subunit 1	-3.39	3.98.E-03
80895	ILKAP	ILK associated serine/threonine phosphatase	-3.39	4.01.E-03
64116	SLC39A8	solute carrier family 39 member 8	3.38	4.01.E-03
1152	CKB	creatine kinase B	-3.38	4.02.E-03
4217	MAP3K5	mitogen-activated protein kinase kinase kinase 5	-3.38	4.06.E-03

2219	FCN1	ficolin 1	3.38	4.06.E-03
124995	MRPL10	mitochondrial ribosomal protein L10	-3.38	4.09.E-03
397	ARHGDIB	Rho GDP dissociation inhibitor beta	3.38	4.10.E-03
25801	GCA	grancalcin	3.38	4.10.E-03
5604	MAP2K1	mitogen-activated protein kinase kinase 1	3.38	4.12.E-03
6159	RPL29	ribosomal protein L29	-3.38	4.13.E-03
912	CD1D	CD1d molecule	3.37	4.14.E-03
164091	PAQR7	progestin and adipoQ receptor family member 7	3.37	4.18.E-03
126364	LRRC25	leucine rich repeat containing 25	3.37	4.17.E-03
3643	INSR	insulin receptor	-3.37	4.17.E-03
51065	RPS27L	ribosomal protein S27 like	3.37	4.18.E-03
55527	FEM1A	fem-1 homolog A	-3.37	4.18.E-03
10213	PSMD14	proteasome 26S subunit, non-ATPase 14	3.37	4.19.E-03
79148	MMP28	matrix metallopeptidase 28	3.37	4.19.E-03
27335	EIF3K	eukaryotic translation initiation factor 3 subunit K	-3.37	4.19.E-03
10247	RIDA	reactive intermediate imine deaminase A homolog	-3.37	4.20.E-03
653140	FAM228A	family with sequence similarity 228 member A	-3.37	4.21.E-03
9182	RASSF9	Ras association domain family member 9	3.37	4.22.E-03
59348	ZNF350	zinc finger protein 350	3.36	4.24.E-03
5476	CTSA	cathepsin A	3.36	4.24.E-03
1520	CTSS	cathepsin S	3.36	4.24.E-03
10390	CEPT1	choline/ethanolamine phosphotransferase 1	-3.36	4.26.E-03
5096	PCCB	propionyl-CoA carboxylase beta subunit	-3.36	4.26.E-03
170954	PPP1R18	protein phosphatase 1 regulatory subunit 18	3.36	4.25.E-03
2006	ELN	elastin	3.36	4.27.E-03
2110	ETFDH	electron transfer flavoprotein dehydrogenase	-3.36	4.28.E-03
9159	PCSK7	proprotein convertase subtilisin/kexin type 7	3.36	4.29.E-03
8635	RNASET2	ribonuclease T2	3.36	4.29.E-03
1307	COL16A1	collagen type XVI alpha 1 chain	3.35	4.33.E-03
152137	CCDC50	coiled-coil domain containing 50	-3.35	4.36.E-03
81493	SYNC	syncoilin, intermediate filament protein	3.35	4.37.E-03
2888	GRB14	growth factor receptor bound protein 14	3.35	4.37.E-03
23396	PIP5K1C	phosphatidylinositol-4-phosphate 5-kinase type 1 gamma	3.35	4.37.E-03
2212	FCGR2A	Fc fragment of IgG receptor IIa	3.35	4.37.E-03
375287	RBM43	RNA binding motif protein 43	-3.35	4.39.E-03
26031	OSBPL3	oxysterol binding protein like 3	3.35	4.39.E-03
1071	CETP	cholesteryl ester transfer protein	-3.35	4.39.E-03
219972	MPEG1	macrophage expressed 1	3.35	4.40.E-03
4719	NDUFS1	NADH:ubiquinone oxidoreductase core subunit S1	-3.35	4.42.E-03
93594	TBC1D31	TBC1 domain family member 31	3.35	4.42.E-03
254042	METAP1D	methionyl aminopeptidase type 1D, mitochondrial	-3.35	4.41.E-03
11026	LILRA3	leukocyte immunoglobulin like receptor A3	3.34	4.46.E-03
8521	GCM1	glial cells missing homolog 1	-3.34	4.46.E-03
26053	AUTS2	autism susceptibility candidate 2	-3.34	4.46.E-03
10935	PRDX3	peroxiredoxin 3	-3.34	4.47.E-03
8078	USP5	ubiquitin specific peptidase 5	3.34	4.49.E-03
2760	GM2A	GM2 ganglioside activator	3.34	4.49.E-03
120425	JAML	junction adhesion molecule like	3.34	4.51.E-03
84885	ZDHHC12	zinc finger DHHC-type containing 12	3.34	4.52.E-03
131873	COL6A6	collagen type VI alpha 6 chain	-3.34	4.51.E-03
1778	DYNC1H1	dynein cytoplasmic 1 heavy chain 1	3.34	4.51.E-03
27303	RBMS3	RNA binding motif single stranded interacting protein 3	-3.34	4.52.E-03
6988	TCTA	T-cell leukemia translocation altered	3.34	4.53.E-03
112609	MRAP2	melanocortin 2 receptor accessory protein 2	3.33	4.56.E-03
4851	NOTCH1	notch 1	-3.33	4.56.E-03
25903	OLFML2B	olfactomedin like 2B	3.33	4.63.E-03
51181	DCXR	dicarbonyl and L-xylulose reductase	-3.33	4.65.E-03
22949	PTGR1	prostaglandin reductase 1	-3.33	4.64.E-03
51027	BOLA1	bolA family member 1	-3.33	4.66.E-03
84952	CGNL1	cingulin like 1	3.33	4.66.E-03
10990	LILRB5	leukocyte immunoglobulin like receptor B5	3.33	4.67.E-03
1149	CIDEA	cell death-inducing DFFA-like effector a	-3.33	4.67.E-03
683	BST1	bone marrow stromal cell antigen 1	3.32	4.69.E-03
8835	SOCS2	suppressor of cytokine signaling 2	-3.32	4.69.E-03
9371	KIF3B	kinesin family member 3B	3.32	4.69.E-03
4012	LNPEP	leucyl and cystinyl aminopeptidase	3.32	4.68.E-03
9791	PTDSS1	phosphatidylserine synthase 1	3.32	4.69.E-03

25911	DPCD	deleted in primary ciliary dyskinesia homolog (mouse)	3.32	4.70.E-03
23078	VWA8	von Willebrand factor A domain containing 8	-3.32	4.73.E-03
63923	TNN	tenascin N	-3.32	4.77.E-03
8407	TAGLN2	transgelin 2	3.32	4.80.E-03
55240	STEAP3	STEAP3 metalloreductase	3.32	4.81.E-03
25804	LSM4	LSM4 homolog, U6 small nuclear RNA and mRNA degradation associated	3.32	4.81.E-03
79007	DBNDD1	dysbindin domain containing 1	3.31	4.81.E-03
23473	CAPN7	calpain 7	-3.31	4.82.E-03
54931	TRMT10C	tRNA methyltransferase 10C, mitochondrial RNase P subunit	-3.31	4.83.E-03
2644	GCHFR	GTP cyclohydrolase I feedback regulator	-3.31	4.86.E-03
9738	CCP110	centriolar coiled-coil protein 110	-3.31	4.86.E-03
113402	SFT2D1	SFT2 domain containing 1	-3.31	4.85.E-03
27163	NAAA	N-acylethanolamine acid amidase	3.31	4.85.E-03
6230	RPS25	ribosomal protein S25	-3.31	4.87.E-03
23408	SIRT5	sirtuin 5	-3.31	4.87.E-03
26509	MYOF	myoferlin	3.31	4.89.E-03
4068	SH2D1A	SH2 domain containing 1A	3.31	4.89.E-03
9934	P2RY14	purinergic receptor P2Y14	3.30	4.93.E-03
1725	DHPS	deoxyhypusine synthase	-3.30	4.94.E-03
3570	IL6R	interleukin 6 receptor	3.30	4.95.E-03
4125	MAN2B1	mannosidase alpha class 2B member 1	3.30	4.95.E-03
3055	HCK	HCK proto-oncogene, Src family tyrosine kinase	3.30	4.95.E-03
400451	FAM174B	family with sequence similarity 174 member B	3.30	4.97.E-03
635	BHMT	betaine--homocysteine S-methyltransferase	3.30	4.98.E-03
2054	STX2	syntaxin 2	3.30	4.99.E-03
51428	DDX41	DEAD-box helicase 41	3.30	5.00.E-03
9638	FEZ1	fasciculation and elongation protein zeta 1	3.30	5.01.E-03
4671	NAIP	NLR family apoptosis inhibitory protein	3.30	5.01.E-03
91694	LONRF1	LON peptidase N-terminal domain and ring finger 1	-3.30	5.01.E-03
1956	EGFR	epidermal growth factor receptor	-3.29	5.07.E-03
51085	MLXIPL	MLX interacting protein like	-3.29	5.07.E-03
3257	HPS1	HPS1, biogenesis of lysosomal organelles complex 3 subunit 1	3.29	5.07.E-03
5443	POMC	proopiomelanocortin	-3.29	5.10.E-03
123096	SLC25A29	solute carrier family 25 member 29	-3.29	5.11.E-03
6688	SPI1	Spi-1 proto-oncogene	3.29	5.10.E-03
7251	TSG101	tumor susceptibility 101	-3.29	5.11.E-03
80221	ACSF2	acyl-CoA synthetase family member 2	-3.29	5.11.E-03
23524	SRRM2	serine/arginine repetitive matrix 2	-3.29	5.15.E-03
161742	SPRED1	sprouty related EVH1 domain containing 1	3.29	5.14.E-03
29979	UBQLN1	ubiquilin 1	-3.29	5.17.E-03
22887	FOXJ3	forkhead box J3	-3.29	5.17.E-03
1611	DAP	death associated protein	3.29	5.17.E-03
27075	TSPAN13	tetraspanin 13	-3.29	5.17.E-03
5728	PTEN	phosphatase and tensin homolog	-3.28	5.18.E-03
2788	GNG7	G protein subunit gamma 7	-3.28	5.18.E-03
3663	IRF5	interferon regulatory factor 5	3.28	5.23.E-03
403340	MGC70870	C-terminal binding protein 2 pseudogene	-3.28	5.29.E-03
22919	MAPRE1	microtubule associated protein RP/EB family member 1	3.28	5.29.E-03
326624	RAB37	RAB37, member RAS oncogene family	3.28	5.29.E-03
3396	MRPL58	mitochondrial ribosomal protein L58	-3.28	5.29.E-03
10212	DDX39A	DEAD-box helicase 39A	3.28	5.33.E-03
3329	HSPD1	heat shock protein family D (Hsp60) member 1	-3.28	5.32.E-03
92014	SLC25A51	solute carrier family 25 member 51	-3.28	5.33.E-03
339541	C1orf228	chromosome 1 open reading frame 228	3.27	5.35.E-03
23048	FNBP1	formin binding protein 1	3.27	5.37.E-03
4283	CXCL9	C-X-C motif chemokine ligand 9	3.27	5.37.E-03
4000	LMNA	lamin A/C	3.27	5.40.E-03
57599	WDR48	WD repeat domain 48	-3.27	5.42.E-03
1192	CLIC1	chloride intracellular channel 1	3.27	5.44.E-03
55055	ZWILCH	zwilch kinetochore protein	3.27	5.45.E-03
8878	SQSTM1	sequestosome 1	3.27	5.45.E-03
9454	HOMER3	homer scaffolding protein 3	3.27	5.45.E-03
56731	SLC2A4RG	SLC2A4 regulator	-3.26	5.46.E-03
9747	TCAF1	TRPM8 channel associated factor 1	-3.26	5.46.E-03
976	ADGRE5	adhesion G protein-coupled receptor E5	3.26	5.47.E-03
9214	FCMR	Fc fragment of IgM receptor	3.26	5.47.E-03
27109	ATP5S	ATP synthase, H+ transporting, mitochondrial Fo complex subunit s (factor B)	-3.26	5.50.E-03

84102	SLC41A2	solute carrier family 41 member 2	3.26	5.50.E-03
6840	SVIL	supervillin	-3.26	5.51.E-03
158293	FAM120AOS	family with sequence similarity 120A opposite strand	-3.26	5.53.E-03
3425	IDUA	iduronidase, alpha-L-	3.26	5.52.E-03
168002	DACT2	dishevelled binding antagonist of beta catenin 2	-3.26	5.55.E-03
8975	USP13	ubiquitin specific peptidase 13 (isopeptidase T-3)	-3.26	5.55.E-03
57573	ZNF471	zinc finger protein 471	-3.26	5.57.E-03
381	ARF5	ADP ribosylation factor 5	3.26	5.56.E-03
23604	DAPK2	death associated protein kinase 2	-3.25	5.60.E-03
53829	P2RY13	purinergic receptor P2Y13	3.25	5.63.E-03
10462	CLEC10A	C-type lectin domain family 10 member A	3.25	5.64.E-03
55825	PECR	peroxisomal trans-2-enoyl-CoA reductase	-3.25	5.65.E-03
4924	NUCB1	nucleobindin 1	3.25	5.65.E-03
151473	SLC16A14	solute carrier family 16 member 14	-3.25	5.72.E-03
9	NAT1	N-acetyltransferase 1	3.25	5.71.E-03
57446	NDRG3	NDRG family member 3	3.25	5.71.E-03
23225	NUP210	nucleoporin 210	3.25	5.71.E-03
10011	SRA1	steroid receptor RNA activator 1	3.25	5.71.E-03
4354	MPP1	membrane palmitoylated protein 1	3.25	5.72.E-03
6944	VPS72	vacuolar protein sorting 72 homolog	-3.25	5.74.E-03
533	ATP6V0B	ATPase H+ transporting V0 subunit b	3.25	5.73.E-03
84034	EMILIN2	elastin microfibril interfacer 2	3.24	5.75.E-03
1117	CHI3L2	chitinase 3 like 2	3.24	5.79.E-03
1025	CDK9	cyclin dependent kinase 9	-3.24	5.81.E-03
4916	NTRK3	neurotrophic receptor tyrosine kinase 3	-3.24	5.84.E-03
221395	ADGRF5	adhesion G protein-coupled receptor F5	-3.24	5.85.E-03
113802	HENMT1	HEN1 methyltransferase homolog 1	3.24	5.86.E-03
79022	TMEM106C	transmembrane protein 106C	-3.24	5.90.E-03
5264	PHYH	phytanoyl-CoA 2-hydroxylase	-3.23	5.96.E-03
55315	SLC29A3	solute carrier family 29 member 3	3.23	5.97.E-03
5711	PSMD5	proteasome 26S subunit, non-ATPase 5	-3.23	5.97.E-03
64718	UNKL	unkempt family like zinc finger	3.23	5.98.E-03
1329	COX5B	cytochrome c oxidase subunit 5B	-3.23	6.08.E-03
2359	FPR3	formyl peptide receptor 3	3.22	6.13.E-03
404672	GTF2H5	general transcription factor IIH subunit 5	3.22	6.14.E-03
54884	RETSAT	retinol saturase	3.22	6.14.E-03
23286	WWC1	WW and C2 domain containing 1	3.22	6.13.E-03
1186	CLCN7	chloride voltage-gated channel 7	3.22	6.14.E-03
1318	SLC31A2	solute carrier family 31 member 2	3.22	6.15.E-03
10891	PPARGC1A	PPARG coactivator 1 alpha	-3.22	6.17.E-03
463	ZFHX3	zinc finger homeobox 3	3.22	6.17.E-03
5255	PHKA1	phosphorylase kinase regulatory subunit alpha 1	-3.22	6.19.E-03
23313	KIAA0930	KIAA0930	3.22	6.22.E-03
10124	ARL4A	ADP ribosylation factor like GTPase 4A	3.22	6.23.E-03
115330	GPR146	G protein-coupled receptor 146	-3.22	6.22.E-03
7084	TK2	thymidine kinase 2, mitochondrial	3.22	6.22.E-03
10131	TRAP1	TNF receptor associated protein 1	-3.22	6.23.E-03
1856	DVL2	dishevelled segment polarity protein 2	-3.22	6.23.E-03
7010	TEK	TEK receptor tyrosine kinase	-3.22	6.23.E-03
6926	TBX3	T-box 3	-3.21	6.24.E-03
23483	TGDS	TDP-glucose 4,6-dehydratase	-3.21	6.26.E-03
10884	MRPS30	mitochondrial ribosomal protein S30	-3.21	6.25.E-03
8575	PRKRA	protein activator of interferon induced protein kinase EIF2AK2	-3.21	6.29.E-03
473	RERE	arginine-glutamic acid dipeptide repeats	-3.21	6.28.E-03
55892	MYNN	myoneurin	-3.21	6.36.E-03
80746	TSEN2	tRNA splicing endonuclease subunit 2	-3.21	6.35.E-03
51311	TLR8	toll like receptor 8	3.21	6.35.E-03
5236	PGM1	phosphoglucomutase 1	-3.21	6.35.E-03
3684	ITGAM	integrin subunit alpha M	3.21	6.38.E-03
65005	MRPL9	mitochondrial ribosomal protein L9	-3.20	6.41.E-03
7409	VAV1	vav guanine nucleotide exchange factor 1	3.20	6.44.E-03
51175	TUBE1	tubulin epsilon 1	-3.20	6.44.E-03
9267	CYTH1	cytohesin 1	3.20	6.45.E-03
64222	TOR3A	torsin family 3 member A	3.20	6.45.E-03
10128	LRPPRC	leucine rich pentatricopeptide repeat containing	-3.20	6.46.E-03
80237	ELL3	elongation factor for RNA polymerase II 3	3.20	6.47.E-03
64757	MARC1	mitochondrial amidoxime reducing component 1	-3.20	6.50.E-03

51294	PCDH12	protocadherin 12	-3.20	6.51.E-03
1350	COX7C	cytochrome c oxidase subunit 7C	-3.20	6.52.E-03
4332	MNDA	myeloid cell nuclear differentiation antigen	3.20	6.54.E-03
55713	ZNF334	zinc finger protein 334	-3.20	6.58.E-03
6258	RXRG	retinoid X receptor gamma	-3.19	6.60.E-03
6423	SFRP2	secreted frizzled related protein 2	3.19	6.60.E-03
79035	NABP2	nucleic acid binding protein 2	3.19	6.60.E-03
23629	BRD7P3	bromodomain containing 7 pseudogene 3	-3.19	6.63.E-03
116362	RBP7	retinol binding protein 7	-3.19	6.66.E-03
8334	HIST1H2AC	histone cluster 1 H2A family member c	3.19	6.66.E-03
1317	SLC31A1	solute carrier family 31 member 1	3.19	6.69.E-03
10149	ADGRG2	adhesion G protein-coupled receptor G2	-3.19	6.70.E-03
23788	MTCH2	mitochondrial carrier 2	3.19	6.70.E-03
55245	UQCC1	ubiquinol-cytochrome c reductase complex assembly factor 1	-3.19	6.73.E-03
80731	THSD7B	thrombospondin type 1 domain containing 7B	-3.18	6.76.E-03
25886	POC1A	POC1 centriolar protein A	3.18	6.76.E-03
57647	DHX37	DEAH-box helicase 37	3.18	6.77.E-03
9450	LY86	lymphocyte antigen 86	3.18	6.77.E-03
84062	DTNBP1	dystrobrevin binding protein 1	3.18	6.82.E-03
4810	NHS	NHS actin remodeling regulator	-3.18	6.82.E-03
8906	AP1G2	adaptor related protein complex 1 gamma 2 subunit	3.18	6.82.E-03
50651	SLC45A1	solute carrier family 45 member 1	-3.18	6.94.E-03
5162	PDHB	pyruvate dehydrogenase (lipoamide) beta	-3.18	6.94.E-03
5292	PIM1	Pim-1 proto-oncogene, serine/threonine kinase	3.17	6.99.E-03
4124	MAN2A1	mannosidase alpha class 2A member 1	3.17	7.00.E-03
64759	TNS3	tensin 3	3.17	6.99.E-03
175	AGA	aspartylglucosaminidase	3.17	6.99.E-03
6581	SLC22A3	solute carrier family 22 member 3	-3.17	7.00.E-03
92999	ZBTB47	zinc finger and BTB domain containing 47	3.17	7.00.E-03
65985	AACS	acetoacetyl-CoA synthetase	-3.17	7.03.E-03
6821	SUOX	sulfite oxidase	-3.17	7.02.E-03
55116	TMEM39B	transmembrane protein 39B	3.16	7.14.E-03
112770	GLMP	glycosylated lysosomal membrane protein	3.16	7.16.E-03
57509	MTUS1	microtubule associated tumor suppressor 1	-3.16	7.15.E-03
54438	GFOD1	glucose-fructose oxidoreductase domain containing 1	-3.16	7.16.E-03
11329	STK38	serine/threonine kinase 38	3.16	7.16.E-03
890	CCNA2	cyclin A2	3.16	7.17.E-03
2328	FMO3	flavin containing monooxygenase 3	3.16	7.17.E-03
526	ATP6V1B2	ATPase H+ transporting V1 subunit B2	3.16	7.18.E-03
132158	GLYCTK	glycerate kinase	-3.16	7.18.E-03
256949	KANK3	KN motif and ankyrin repeat domains 3	-3.16	7.23.E-03
80321	CEP70	centrosomal protein 70	-3.16	7.27.E-03
9601	PDIA4	protein disulfide isomerase family A member 4	3.16	7.27.E-03
6324	SCN1B	sodium voltage-gated channel beta subunit 1	3.15	7.37.E-03
64395	GMCL1	germ cell-less, spermatogenesis associated 1	-3.15	7.36.E-03
84245	MRI1	methylthioribose-1-phosphate isomerase 1	-3.15	7.36.E-03
4129	MAOB	monoamine oxidase B	-3.15	7.36.E-03
51765	STK26	serine/threonine protein kinase 26	-3.15	7.36.E-03
2673	GFPT1	glutamine--fructose-6-phosphate transaminase 1	-3.15	7.36.E-03
114790	STK11IP	serine/threonine kinase 11 interacting protein	3.15	7.35.E-03
6197	RPS6KA3	ribosomal protein S6 kinase A3	3.15	7.35.E-03
286148	DPY19L4	dpy-19 like 4 (C. elegans)	-3.15	7.46.E-03
145389	SLC38A6	solute carrier family 38 member 6	3.15	7.46.E-03
1495	CTNNA1	catenin alpha 1	3.15	7.48.E-03
7316	UBC	ubiquitin C	3.15	7.48.E-03
9324	HMGN3	high mobility group nucleosomal binding domain 3	-3.15	7.48.E-03
2729	GCLC	glutamate-cysteine ligase catalytic subunit	-3.15	7.48.E-03
7153	TOP2A	topoisomerase (DNA) II alpha	3.15	7.49.E-03
1201	CLN3	CLN3, battenin	3.15	7.48.E-03
22822	PHLDA1	pleckstrin homology like domain family A member 1	3.15	7.48.E-03
22899	ARHGEF15	Rho guanine nucleotide exchange factor 15	-3.15	7.48.E-03
125476	INO80C	INO80 complex subunit C	-3.15	7.47.E-03
23350	U2SURP	U2 snRNP associated SURP domain containing	-3.14	7.51.E-03
197257	LDHD	lactate dehydrogenase D	-3.14	7.55.E-03
137835	TMEM71	transmembrane protein 71	3.14	7.60.E-03
7164	TPD52L1	tumor protein D52-like 1	-3.14	7.60.E-03
116447	TOP1MT	topoisomerase (DNA) I, mitochondrial	3.14	7.61.E-03

217	ALDH2	aldehyde dehydrogenase 2 family (mitochondrial)	-3.14	7.63.E-03
313	AOAH	acyloxyacyl hydrolase	3.14	7.65.E-03
11153	FICD	FIC domain containing	3.14	7.64.E-03
348094	ANKDD1A	ankyrin repeat and death domain containing 1A	3.14	7.67.E-03
79364	ZXDC	ZXD family zinc finger C	-3.14	7.68.E-03
54993	ZSCAN2	zinc finger and SCAN domain containing 2	-3.14	7.69.E-03
5350	PLN	phospholamban	-3.13	7.73.E-03
29108	PYCARD	PYD and CARD domain containing	3.13	7.74.E-03
2937	GSS	glutathione synthetase	3.13	7.81.E-03
1716	DGUOK	deoxyguanosine kinase	-3.13	7.81.E-03
23368	PPP1R13B	protein phosphatase 1 regulatory subunit 13B	-3.13	7.86.E-03
5020	OXT	oxytocin/neurophysin I prepropeptide	3.13	7.86.E-03
6611	SMS	spermine synthase	3.13	7.86.E-03
23499	MACF1	microtubule-actin crosslinking factor 1	3.13	7.92.E-03
27430	MAT2B	methionine adenosyltransferase 2B	-3.13	7.92.E-03
5742	PTGS1	prostaglandin-endoperoxide synthase 1	3.12	7.95.E-03
84988	PPP1R16A	protein phosphatase 1 regulatory subunit 16A	-3.12	7.95.E-03
79161	TMEM243	transmembrane protein 243	-3.12	7.95.E-03
286530	P2RY8	purinergic receptor P2Y8	3.12	7.99.E-03
56995	TULP4	tubby like protein 4	3.12	7.99.E-03
9367	RAB9A	RAB9A, member RAS oncogene family	3.12	8.04.E-03
3916	LAMP1	lysosomal associated membrane protein 1	3.12	8.04.E-03
6584	SLC22A5	solute carrier family 22 member 5	3.12	8.05.E-03
2268	FGR	FGR proto-oncogene, Src family tyrosine kinase	3.12	8.05.E-03
23300	ATMIN	ATM interactor	-3.12	8.05.E-03
51360	MBTPS2	membrane bound transcription factor peptidase, site 2	-3.12	8.05.E-03
5603	MAPK13	mitogen-activated protein kinase 13	3.12	8.05.E-03
11231	SEC63	SEC63 homolog, protein translocation regulator	3.12	8.05.E-03
146894	CD300LG	CD300 molecule like family member g	-3.12	8.09.E-03
9950	GOLGA5	golgin A5	3.11	8.12.E-03
165324	UBXN2A	UBX domain protein 2A	-3.11	8.11.E-03
8884	SLC5A6	solute carrier family 5 member 6	-3.11	8.15.E-03
148741	ANKRD35	ankyrin repeat domain 35	3.11	8.18.E-03
80198	MUS81	MUS81 structure-specific endonuclease subunit	-3.11	8.22.E-03
3937	LCP2	lymphocyte cytosolic protein 2	3.11	8.24.E-03
4327	MMP19	matrix metallopeptidase 19	3.11	8.24.E-03
221908	PPP1R35	protein phosphatase 1 regulatory subunit 35	-3.11	8.26.E-03
57414	RHBDD2	rhomboid domain containing 2	3.11	8.26.E-03
761	CA3	carbonic anhydrase 3	-3.11	8.26.E-03
1312	COMT	catechol-O-methyltransferase	3.11	8.28.E-03
29902	FAM216A	family with sequence similarity 216 member A	3.11	8.30.E-03
127670	TEDDM1	transmembrane epididymal protein 1	-3.11	8.30.E-03
1244	ABCC2	ATP binding cassette subfamily C member 2	3.11	8.29.E-03
92002	FAM58A	family with sequence similarity 58 member A	3.10	8.32.E-03
51409	HEMK1	HemK methyltransferase family member 1	-3.10	8.37.E-03
6993	DYNLT1	dynein light chain Tctex-type 1	3.10	8.37.E-03
57685	CACHD1	cache domain containing 1	-3.10	8.37.E-03
10670	RRAGA	Ras related GTP binding A	-3.10	8.38.E-03
8402	SLC25A11	solute carrier family 25 member 11	-3.10	8.37.E-03
57580	PREX1	phosphatidylinositol-3,4,5-trisphosphate dependent Rac exchange factor 1	3.10	8.39.E-03
84519	ACRBP	acrosin binding protein	3.10	8.39.E-03
7057	THBS1	thrombospondin 1	3.10	8.39.E-03
6041	RNASEL	ribonuclease L	3.10	8.40.E-03
64960	MRPS15	mitochondrial ribosomal protein S15	-3.10	8.40.E-03
8546	AP3B1	adaptor related protein complex 3 beta 1 subunit	3.10	8.40.E-03
8428	STK24	serine/threonine kinase 24	-3.10	8.41.E-03
9055	PRC1	protein regulator of cytokinesis 1	3.10	8.41.E-03
5176	SERPINF1	serpin family F member 1	3.10	8.41.E-03
23266	ADGRL2	adhesion G protein-coupled receptor L2	-3.10	8.43.E-03
1978	EIF4EBP1	eukaryotic translation initiation factor 4E binding protein 1	-3.10	8.43.E-03
2526	FUT4	fucosyltransferase 4	3.10	8.43.E-03
6256	RXRA	retinoid X receptor alpha	-3.10	8.42.E-03
5701	PSMC2	proteasome 26S subunit, ATPase 2	3.10	8.42.E-03
9509	ADAMTS2	ADAM metallopeptidase with thrombospondin type 1 motif 2	3.10	8.41.E-03
64425	POLR1E	RNA polymerase I subunit E	-3.10	8.42.E-03
1173	AP2M1	adaptor related protein complex 2 mu 1 subunit	3.10	8.41.E-03
27152	INTU	inturned planar cell polarity protein	-3.09	8.47.E-03

5101	PCDH9	protocadherin 9	3.09	8.52.E-03
3315	HSPB1	heat shock protein family B (small) member 1	3.09	8.52.E-03
283638	CEP170B	centrosomal protein 170B	3.09	8.52.E-03
6141	RPL18	ribosomal protein L18	-3.09	8.52.E-03
84148	KAT8	lysine acetyltransferase 8	-3.09	8.55.E-03
23005	MAPKBP1	mitogen-activated protein kinase binding protein 1	3.09	8.58.E-03
83543	AIF1L	allograft inflammatory factor 1 like	-3.09	8.58.E-03
221472	FGD2	FYVE, RhoGEF and PH domain containing 2	3.09	8.59.E-03
845	CASQ2	calsequestrin 2	-3.09	8.63.E-03
118424	UBE2J2	ubiquitin conjugating enzyme E2 J2	-3.09	8.63.E-03
23012	STK38L	serine/threonine kinase 38 like	3.09	8.63.E-03
1164	CKS2	CDC28 protein kinase regulatory subunit 2	3.09	8.64.E-03
60487	TRMT11	tRNA methyltransferase 11 homolog	-3.09	8.65.E-03
11137	PWP1	PWP1 homolog, endonuclein	3.09	8.66.E-03
2202	EFEMP1	EGF containing fibulin like extracellular matrix protein 1	3.08	8.70.E-03
90390	MED30	mediator complex subunit 30	-3.08	8.72.E-03
1983	EIF5	eukaryotic translation initiation factor 5	-3.08	8.73.E-03
79607	FAM118B	family with sequence similarity 118 member B	3.08	8.74.E-03
1777	DNASE2	deoxyribonuclease 2, lysosomal	3.08	8.75.E-03
56910	STARD7	StAR related lipid transfer domain containing 7	3.08	8.77.E-03
5050	PAFAH1B3	platelet activating factor acetylhydrolase 1b catalytic subunit 3	3.08	8.77.E-03
5638	PRRG1	proline rich and Gla domain 1	3.08	8.78.E-03
23731	TMEM245	transmembrane protein 245	-3.08	8.81.E-03
29785	CYP2S1	cytochrome P450 family 2 subfamily S member 1	3.08	8.86.E-03
821	CANX	calnexin	3.07	9.01.E-03
55208	DCUN1D2	defective in cullin neddylation 1 domain containing 2	-3.07	9.06.E-03
119504	ANAPC16	anaphase promoting complex subunit 16	-3.07	9.06.E-03
509	ATP5C1	ATP synthase, H+ transporting, mitochondrial F1 complex, gamma polypeptide 1	-3.07	9.06.E-03
5207	PFKFB1	6-phosphofructo-2-kinase/fructose-2,6-biphosphatase 1	-3.07	9.05.E-03
5002	SLC22A18	solute carrier family 22 member 18	3.07	9.05.E-03
6786	STIM1	stromal interaction molecule 1	3.07	9.07.E-03
771	CA12	carbonic anhydrase 12	3.07	9.07.E-03
338382	RAB7B	RAB7B, member RAS oncogene family	3.06	9.23.E-03
4889	NPY5R	neuropeptide Y receptor Y5	3.06	9.24.E-03
5140	PDE3B	phosphodiesterase 3B	-3.06	9.29.E-03
9563	H6PD	hexose-6-phosphate dehydrogenase/glucose 1-dehydrogenase	3.06	9.31.E-03
55268	ECHDC2	enoyl-CoA hydratase domain containing 2	-3.06	9.30.E-03
56984	PSMG2	proteasome assembly chaperone 2	-3.06	9.31.E-03
29929	ALG6	ALG6, alpha-1,3-glucosyltransferase	3.06	9.34.E-03
55074	OXR1	oxidation resistance 1	-3.06	9.38.E-03
80324	PUS1	pseudouridylate synthase 1	3.06	9.39.E-03
285527	FRYL	FRY like transcription coactivator	-3.06	9.39.E-03
8076	MFAP5	microfibrillar associated protein 5	3.06	9.39.E-03
79981	FRMD1	FERM domain containing 1	-3.06	9.41.E-03
7791	ZYX	zyxin	3.06	9.44.E-03
57184	FAM219B	family with sequence similarity 219 member B	-3.06	9.45.E-03
3927	LASP1	LIM and SH3 protein 1	3.06	9.44.E-03
10113	PREB	prolactin regulatory element binding	3.05	9.47.E-03
5446	PON3	paraoxonase 3	-3.05	9.50.E-03
3838	KPNA2	karyopherin subunit alpha 2	3.05	9.54.E-03
10457	GPNMB	glycoprotein nmb	3.05	9.55.E-03
23643	LY96	lymphocyte antigen 96	3.05	9.54.E-03
26512	INTS6	integrator complex subunit 6	-3.05	9.65.E-03
23646	PLD3	phospholipase D family member 3	3.05	9.66.E-03
26018	LRIG1	leucine rich repeats and immunoglobulin like domains 1	-3.05	9.66.E-03
6201	RPS7	ribosomal protein S7	-3.05	9.67.E-03
83541	FAM110A	family with sequence similarity 110 member A	3.05	9.69.E-03
55621	TRMT1	tRNA methyltransferase 1	3.05	9.69.E-03
115677	NOSTRIN	nitric oxide synthase trafficking	-3.04	9.69.E-03
956	ENTPD3	ectonucleoside triphosphate diphosphohydrolase 3	-3.04	9.69.E-03
54948	MRPL16	mitochondrial ribosomal protein L16	-3.04	9.69.E-03
56963	RGMA	repulsive guidance molecule family member a	-3.04	9.71.E-03
285172	FAM126B	family with sequence similarity 126 member B	-3.04	9.72.E-03
55010	PARPBP	PARP1 binding protein	3.04	9.71.E-03
2271	FH	fumarate hydratase	-3.04	9.77.E-03
51135	IRAK4	interleukin 1 receptor associated kinase 4	3.04	9.79.E-03
7169	TPM2	tropomyosin 2 (beta)	3.04	9.79.E-03

6300	MAPK12	mitogen-activated protein kinase 12	-3.04	9.82.E-03
2055	CLN8	ceroid-lipofuscinosis, neuronal 8	3.04	9.81.E-03
391	RHOG	ras homolog family member G	3.04	9.88.E-03
9659	PDE4DIP	phosphodiesterase 4D interacting protein	3.04	9.89.E-03
338328	GPIHBP1	glycosylphosphatidylinositol anchored high density lipoprotein binding protein 1	-3.04	9.91.E-03
3759	KCNJ2	potassium voltage-gated channel subfamily J member 2	3.03	9.93.E-03
51604	PIGT	phosphatidylinositol glycan anchor biosynthesis class T	3.03	9.96.E-03
27034	ACAD8	acyl-CoA dehydrogenase family member 8	-3.03	9.99.E-03

Table S2. The identified cross-species drug-signatures.

Gene id	Symbol	Gene name	Z-score	FDR
8870	IER3	immediate early response 3	7.33	0.00.E+00
1979	EIF4EBP2	eukaryotic translation initiation factor 4E binding protein 2	-7.18	0.00.E+00
11043	MID2	midline 2	-6.99	0.00.E+00
56301	SLC7A10	solute carrier family 7 (neutral amino acid transporter light chain, asc system), member 10	-6.78	0.00.E+00
4337	MOCS1	molybdenum cofactor synthesis 1	-6.70	0.00.E+00
25975	EGFL6	EGF-like-domain, multiple 6	6.58	0.00.E+00
3952	LEP	leptin	6.49	0.00.E+00
64756	ATPAF1	ATP synthase mitochondrial F1 complex assembly factor 1	-6.38	0.00.E+00
3929	LBP	lipopolysaccharide binding protein	6.11	0.00.E+00
2650	GCNT1	glucosaminyl (N-acetyl) transferase 1, core 2	6.07	0.00.E+00
5216	PFN1	profilin 1	6.03	0.00.E+00
9051	PSTPIP1	proline-serine-threonine phosphatase interacting protein 1	5.81	0.00.E+00
3791	KDR	kinase insert domain receptor	-5.63	0.00.E+00
1959	EGR2	early growth response 2	5.62	0.00.E+00
595	CCND1	cyclin D1	5.36	0.00.E+00
83943	IMMP2L	inner mitochondrial membrane peptidase subunit 2	-5.35	0.00.E+00
6271	S100A1	S100 calcium binding protein A1	-5.33	0.00.E+00
7481	WNT11	wingless-type MMTV integration site family, member 11	-5.31	0.00.E+00
2872	MKNK2	MAP kinase interacting serine/threonine kinase 2	-5.31	0.00.E+00
6275	S100A4	S100 calcium binding protein A4	5.30	0.00.E+00
153579	BTNL9	butyrophilin-like 9	-5.25	7.94.E-06
4329	ALDH6A1	aldehyde dehydrogenase 6 family, member A1	-5.23	7.87.E-06
28999	KLF15	Kruppel-like factor 15	-5.23	7.81.E-06
594	BCKDHB	branched chain keto acid dehydrogenase E1, beta polypeptide	-5.18	7.46.E-06
5827	PXMP2	peroxisomal membrane protein 2, 22kDa	-5.11	1.40.E-05
7941	PLA2G7	phospholipase A2, group VII (platelet-activating factor acetylhydrolase, plasma)	5.02	1.27.E-05
1528	CYB5A	cytochrome b5 type A (microsomal)	-4.90	4.84.E-05
5973	RENBP	renin binding protein	4.89	5.18.E-05
10170	DHRS9	dehydrogenase/reductase (SDR family) member 9	4.88	5.15.E-05
57520	HECW2	HECT, C2 and WW domain containing E3 ubiquitin protein ligase 2	-4.82	7.62.E-05
2769	GNA15	guanine nucleotide binding protein (G protein), alpha 15 (Gq class)	4.82	7.58.E-05
219855	SLC37A2	solute carrier family 37 (glucose-6-phosphate transporter), member 2	4.81	8.02.E-05
84706	GPT2	glutamic pyruvate transaminase (alanine aminotransferase) 2	-4.80	7.87.E-05
5027	P2RX7	purinergic receptor P2X, ligand gated ion channel, 7	4.78	8.29.E-05
8800	PEX11A	peroxisomal biogenesis factor 11 alpha	-4.77	8.14.E-05
38	ACAT1	acetyl-CoA acetyltransferase 1	-4.77	8.11.E-05
23175	LPIN1	lipin 1	-4.75	9.21.E-05
5164	PDK2	pyruvate dehydrogenase kinase, isozyme 2	-4.75	9.17.E-05
9710	KIAA0355	KIAA0355	-4.74	9.09.E-05
1476	CSTB	cystatin B (stefin B)	4.74	9.44.E-05
5660	PSAP	prosaposin	4.74	9.40.E-05
80727	TTYH3	tweety family member 3	4.73	1.05.E-04
4131	MAP1B	microtubule-associated protein 1B	4.67	1.38.E-04
713	C1QB	complement component 1, q subcomponent, B chain	4.67	1.48.E-04
56833	SLAMF8	SLAM family member 8	4.66	1.46.E-04
4015	LOX	lysyl oxidase	4.65	1.47.E-04
56922	MCCC1	methylcrotonoyl-CoA carboxylase 1 (alpha)	-4.63	1.50.E-04
5209	PFKFB3	6-phosphofructo-2-kinase/fructose-2,6-biphosphatase 3	-4.59	1.68.E-04
28514	DLL1	delta-like 1 (Drosophila)	-4.54	1.82.E-04
11326	VSIG4	V-set and immunoglobulin domain containing 4	4.52	1.79.E-04
714	C1QC	complement component 1, q subcomponent, C chain	4.51	1.90.E-04
12	SERPINA3	serpin peptidase inhibitor, clade A (alpha-1 antiproteinase, antitrypsin), member 3	4.50	1.88.E-04
5179	PENK	proenkephalin	4.47	1.96.E-04
84263	HSDL2	hydroxysteroid dehydrogenase like 2	-4.44	2.13.E-04
2331	FMOD	fibromodulin	4.43	2.12.E-04
5025	P2RX4	purinergic receptor P2X, ligand gated ion channel, 4	4.41	2.22.E-04
4481	MSR1	macrophage scavenger receptor 1	4.41	2.25.E-04
6850	SYK	spleen tyrosine kinase	4.40	2.25.E-04
55526	DHTKD1	dehydrogenase E1 and transketolase domain containing 1	-4.39	2.25.E-04
5360	PLTP	phospholipid transfer protein	4.38	2.31.E-04
1436	CSF1R	colony stimulating factor 1 receptor	4.36	2.43.E-04
56243	KIAA1217	KIAA1217	-4.33	2.76.E-04
137872	ADHFE1	alcohol dehydrogenase, iron containing, 1	-4.30	2.98.E-04
2717	GLA	galactosidase, alpha	4.30	3.11.E-04
26227	PHGDH	phosphoglycerate dehydrogenase	-4.25	3.39.E-04
64231	MS4A6A	membrane-spanning 4-domains, subfamily A, member 6A	4.24	3.41.E-04
1031	CDKN2C	cyclin-dependent kinase inhibitor 2C (p18, inhibits CDK4)	-4.23	3.66.E-04

10261	IGSF6	immunoglobulin superfamily, member 6	4.22	3.79.E-04
3074	HEXB	hexosaminidase B (beta polypeptide)	4.21	4.00.E-04
929	CD14	CD14 molecule	4.20	4.06.E-04
712	C1QA	complement component 1, q subcomponent, A chain	4.20	4.11.E-04
55200	PLEKHG6	pleckstrin homology domain containing, family G (with RhoGef domain) member 6	-4.20	4.10.E-04
3101	HK3	hexokinase 3 (white cell)	4.18	4.10.E-04
822	CAPG	capping protein (actin filament), gelsolin-like	4.18	4.16.E-04
10243	GPHN	gephyrin	-4.16	4.30.E-04
80896	NPL	N-acetylneuraminate pyruvate lyase (dihydrodipicolinate synthase)	4.16	4.30.E-04
719	C3AR1	complement component 3a receptor 1	4.14	4.45.E-04
8877	SPHK1	sphingosine kinase 1	4.14	4.46.E-04
29127	RACGAP1	Rac GTPase activating protein 1	4.14	4.51.E-04
9056	SLC7A7	solute carrier family 7 (amino acid transporter light chain, y+L system), member 7	4.12	4.61.E-04
58191	CXCL16	chemokine (C-X-C motif) ligand 16	4.12	4.60.E-04
3001	GZMA	granzyme A (granzyme 1, cytotoxic T-lymphocyte-associated serine esterase 3)	4.11	4.63.E-04
51296	SLC15A3	solute carrier family 15 (oligopeptide transporter), member 3	4.07	5.16.E-04
55013	CCDC109B	coiled-coil domain containing 109B	4.07	5.22.E-04
3162	HMOX1	heme oxygenase 1	4.06	5.33.E-04
4316	MMP7	matrix metallopeptidase 7	4.06	5.37.E-04
5341	PLEK	pleckstrin	4.04	5.76.E-04
10287	RGS19	regulator of G-protein signaling 19	4.04	5.95.E-04
143503	OR51E1	olfactory receptor, family 51, subfamily E, member 1	-4.03	6.01.E-04
54675	CRLS1	cardiolipin synthase 1	-4.03	6.14.E-04
10491	CRTAP	cartilage associated protein	4.02	6.29.E-04
29887	SNX10	sorting nexin 10	3.99	7.09.E-04
4126	MANBA	mannosidase, beta A, lysosomal	3.98	7.23.E-04
2207	FCER1G	Fc fragment of IgE, high affinity I, receptor for; gamma polypeptide	3.98	7.24.E-04
5777	PTPN6	protein tyrosine phosphatase, non-receptor type 6	3.96	7.53.E-04
6696	SPP1	secreted phosphoprotein 1	3.96	7.53.E-04
283431	GAS2L3	growth arrest-specific 2 like 3	3.95	7.56.E-04
9046	DOK2	docking protein 2, 56kDa	3.95	7.76.E-04
2896	GRN	granulin	3.94	7.95.E-04
113	ADCY7	adenylate cyclase 7	3.94	8.00.E-04
3357	HTR2B	5-hydroxytryptamine (serotonin) receptor 2B, G protein-coupled	3.92	8.24.E-04
8673	VAMP8	vesicle-associated membrane protein 8	3.91	8.61.E-04
7305	TYROBP	TYRO protein tyrosine kinase binding protein	3.90	8.79.E-04
32	ACACB	acetyl-CoA carboxylase beta	-3.89	8.93.E-04
64174	DPEP2	dipeptidase 2	3.89	8.98.E-04
3071	NCKAP1L	NCK-associated protein 1-like	3.89	9.02.E-04
79085	SLC25A23	solute carrier family 25 (mitochondrial carrier; phosphate carrier), member 23	-3.88	9.33.E-04
5329	PLAUR	plasminogen activator, urokinase receptor	3.88	9.44.E-04
80704	SLC19A3	solute carrier family 19 (thiamine transporter), member 3	-3.87	9.59.E-04
8476	CDC42BPA	CDC42 binding protein kinase alpha (DMPK-like)	-3.87	9.84.E-04
3059	HCLS1	hematopoietic cell-specific Lyn substrate 1	3.85	1.03.E-03
23406	COTL1	coactosin-like F-actin binding protein 1	3.83	1.11.E-03
53834	FGFRL1	fibroblast growth factor receptor-like 1	-3.81	1.17.E-03
1203	CLN5	ceroid-lipofuscinosis, neuronal 5	3.80	1.21.E-03
10653	SPINT2	serine peptidase inhibitor, Kunitz type, 2	3.78	1.32.E-03
3912	LAMB1	laminin, beta 1	-3.78	1.33.E-03
5641	LGMN	legumain	3.78	1.34.E-03
89846	FGD3	FYVE, RhoGEF and PH domain containing 3	3.77	1.35.E-03
8460	TPST1	tyrosylprotein sulfotransferase 1	-3.76	1.43.E-03
7533	YWHAH	tyrosine 3-monooxygenase/tryptophan 5-monooxygenase activation protein, eta	3.73	1.52.E-03
130399	ACVR1C	activin A receptor, type IC	-3.73	1.52.E-03
6352	CCL5	chemokine (C-C motif) ligand 5	3.71	1.61.E-03
9334	B4GALT5	UDP-Gal:betaGlcNAc beta 1,4- galactosyltransferase, polypeptide 5	3.71	1.61.E-03
2246	FGF1	fibroblast growth factor 1 (acidic)	3.71	1.62.E-03
9308	CD83	CD83 molecule	3.70	1.68.E-03
10870	HCST	hematopoietic cell signal transducer	3.67	1.79.E-03
51284	TLR7	toll-like receptor 7	3.65	1.88.E-03
23462	HEY1	hes-related family bHLH transcription factor with YRPW motif 1	-3.65	1.87.E-03
5880	RAC2	ras-related C3 botulinum toxin substrate 2 (rho family, small GTP binding protein Rac2)	3.64	1.96.E-03
23743	BHMT2	betaine--homocysteine S-methyltransferase 2	3.63	1.99.E-03
2519	FUCA2	fucosidase, alpha-L- 2, plasma	3.62	2.07.E-03
695	BTK	Bruton agammaglobulinemia tyrosine kinase	3.62	2.08.E-03
83442	SH3BGRL3	SH3 domain binding glutamate-rich protein like 3	3.60	2.18.E-03
586	BCAT1	branched chain amino-acid transaminase 1, cytosolic	3.57	2.32.E-03
4689	NCF4	neutrophil cytosolic factor 4, 40kDa	3.57	2.37.E-03
1043	CD52	CD52 molecule	3.55	2.52.E-03

7852	CXCR4	chemokine (C-X-C motif) receptor 4	3.54	2.62.E-03
54491	FAM105A	family with sequence similarity 105, member A	3.53	2.67.E-03
4239	MFAP4	microfibrillar-associated protein 4	3.52	2.78.E-03
366	AQP9	aquaporin 9	3.52	2.79.E-03
4256	MGP	matrix Gla protein	3.51	2.84.E-03
7805	LAPTM5	lysosomal protein transmembrane 5	3.50	2.87.E-03
3099	HK2	hexokinase 2	-3.50	2.88.E-03
64333	ARHGAP9	Rho GTPase activating protein 9	3.48	3.11.E-03
728	C5AR1	complement component 5a receptor 1	3.46	3.23.E-03
8165	AKAP1	A kinase (PRKA) anchor protein 1	-3.46	3.24.E-03
9124	PDLIM1	PDZ and LIM domain 1	3.46	3.26.E-03
64641	EBF2	early B-cell factor 2	-3.45	3.38.E-03
9935	MAFB	v-maf avian musculoaponeurotic fibrosarcoma oncogene homolog B	3.44	3.47.E-03
51338	MS4A4A	membrane-spanning 4-domains, subfamily A, member 4A	3.42	3.65.E-03
11112	HIBADH	3-hydroxyisobutyrate dehydrogenase	-3.41	3.75.E-03
9770	RASSF2	Ras association (RalGDS/AF-6) domain family member 2	3.41	3.75.E-03
1629	DBT	dihydrolipoamide branched chain transacylase E2	-3.41	3.76.E-03
126364	LRRC25	leucine rich repeat containing 25	3.37	4.17.E-03
3643	INSR	insulin receptor	-3.37	4.17.E-03
1520	CTSS	cathepsin S	3.36	4.24.E-03
5096	PCCB	propionyl CoA carboxylase, beta polypeptide	-3.36	4.26.E-03
26031	OSBPL3	oxysterol binding protein-like 3	3.35	4.39.E-03
219972	MPEG1	macrophage expressed 1	3.35	4.40.E-03
683	BST1	bone marrow stromal cell antigen 1	3.32	4.69.E-03
9791	PTDSS1	phosphatidylserine synthase 1	3.32	4.69.E-03
9934	P2RY14	purinergic receptor P2Y, G-protein coupled, 14	3.30	4.93.E-03
4125	MAN2B1	mannosidase, alpha, class 2B, member 1	3.30	4.95.E-03
51085	MLXIPL	MLX interacting protein-like	-3.29	5.07.E-03
6688	SPI1	Spi-1 proto-oncogene	3.29	5.10.E-03
27075	TSPAN13	tetraspanin 13	-3.29	5.17.E-03
5728	PTEN	phosphatase and tensin homolog	-3.28	5.18.E-03
1192	CLIC1	chloride intracellular channel 1	3.27	5.44.E-03
4354	MPP1	membrane protein, palmitoylated 1, 55kDa	3.25	5.72.E-03
533	ATP6V0B	ATPase, H+ transporting, lysosomal 21kDa, V0 subunit b	3.25	5.73.E-03
4916	NTRK3	neurotrophic tyrosine kinase, receptor, type 3	-3.24	5.84.E-03
5264	PHYH	phytanoyl-CoA 2-hydroxylase	-3.23	5.96.E-03
115330	GPR146	G protein-coupled receptor 146	-3.22	6.22.E-03
6926	TBX3	T-box 3	-3.21	6.24.E-03
473	RERE	arginine-glutamic acid dipeptide (RE) repeats	-3.21	6.28.E-03
51311	TLR8	toll-like receptor 8	3.21	6.35.E-03
7409	VAV1	vav 1 guanine nucleotide exchange factor	3.20	6.44.E-03
6258	RXRG	retinoid X receptor, gamma	-3.19	6.60.E-03
116362	RBP7	retinol binding protein 7, cellular	-3.19	6.66.E-03
6581	SLC22A3	solute carrier family 22 (organic cation transporter), member 3	-3.17	7.00.E-03
890	CCNA2	cyclin A2	3.16	7.17.E-03
2328	FMO3	flavin containing monooxygenase 3	3.16	7.17.E-03
526	ATP6V1B2	ATPase, H+ transporting, lysosomal 56/58kDa, V1 subunit B2	3.16	7.18.E-03
4129	MAOB	monoamine oxidase B	-3.15	7.36.E-03
1201	CLN3	ceroid-lipofuscinosis, neuronal 3	3.15	7.48.E-03
22899	ARHGEF15	Rho guanine nucleotide exchange factor (GEF) 15	-3.15	7.48.E-03
29108	PYCARD	PYD and CARD domain containing	3.13	7.74.E-03
23368	PPP1R13B	protein phosphatase 1, regulatory subunit 13B	-3.13	7.86.E-03
84988	PPP1R16A	protein phosphatase 1, regulatory subunit 16A	-3.12	7.95.E-03
2268	FGR	FGR proto-oncogene, Src family tyrosine kinase	3.12	8.05.E-03
146894	CD300LG	CD300 molecule-like family member g	-3.12	8.09.E-03
8884	SLC5A6	solute carrier family 5 (sodium/multivitamin and iodide cotransporter), member 6	-3.11	8.15.E-03
761	CA3	carbonic anhydrase III	-3.11	8.26.E-03
57685	CACHD1	cache domain containing 1	-3.10	8.37.E-03
64960	MRPS15	mitochondrial ribosomal protein S15	-3.10	8.40.E-03
9055	PRC1	protein regulator of cytokinesis 1	3.10	8.41.E-03
1978	EIF4EBP1	eukaryotic translation initiation factor 4E binding protein 1	-3.10	8.43.E-03
6256	RXRA	retinoid X receptor, alpha	-3.10	8.42.E-03
1173	AP2M1	adaptor-related protein complex 2, mu 1 subunit	3.10	8.41.E-03
1164	CKS2	CDC28 protein kinase regulatory subunit 2	3.09	8.64.E-03
5207	PFKFB1	6-phosphofructo-2-kinase/fructose-2,6-biphosphatase 1	-3.07	9.05.E-03
5140	PDE3B	phosphodiesterase 3B, cGMP-inhibited	-3.06	9.29.E-03
7791	ZYX	zyxin	3.06	9.44.E-03
5446	PON3	paraoxonase 3	-3.05	9.50.E-03
10457	GPNMB	glycoprotein (transmembrane) nmb	3.05	9.55.E-03

23643	LY96	lymphocyte antigen 96	3.05	9.54.E-03
23646	PLD3	phospholipase D family, member 3	3.05	9.66.E-03
51135	IRAK4	interleukin-1 receptor-associated kinase 4	3.04	9.79.E-03
6300	MAPK12	mitogen-activated protein kinase 12	-3.04	9.82.E-03
391	RHOG	ras homolog family member G	3.04	9.88.E-03
3759	KCNJ2	potassium channel, inwardly rectifying subfamily J, member 2	3.03	9.93.E-03
